# Core virome shapes adaptation of a phytopathogenic fungus to climate and cropping patterns

**DOI:** 10.1093/ismejo/wrag001

**Published:** 2026-01-12

**Authors:** Kang Zhou, Yue Deng, Chenghuan Zhu, Long Yang, Jing Zhang, Weidong Chen, Nobuhiro Suzuki, Guoqing Li, Mingde Wu

**Affiliations:** State Key Laboratory of Agricultural Microbiology, Huazhong Agricultural University, Wuhan 430070, Hubei Province, China; Hubei Key Laboratory of Plant Pathology, Huazhong Agricultural University, Wuhan 430070, Hubei Province, China; State Key Laboratory of Agricultural Microbiology, Huazhong Agricultural University, Wuhan 430070, Hubei Province, China; Hubei Key Laboratory of Plant Pathology, Huazhong Agricultural University, Wuhan 430070, Hubei Province, China; State Key Laboratory of Agricultural Microbiology, Huazhong Agricultural University, Wuhan 430070, Hubei Province, China; Hubei Key Laboratory of Plant Pathology, Huazhong Agricultural University, Wuhan 430070, Hubei Province, China; State Key Laboratory of Agricultural Microbiology, Huazhong Agricultural University, Wuhan 430070, Hubei Province, China; Hubei Key Laboratory of Plant Pathology, Huazhong Agricultural University, Wuhan 430070, Hubei Province, China; State Key Laboratory of Agricultural Microbiology, Huazhong Agricultural University, Wuhan 430070, Hubei Province, China; Hubei Key Laboratory of Plant Pathology, Huazhong Agricultural University, Wuhan 430070, Hubei Province, China; U.S. Department of Agriculture, Agricultural Research Service, Washington State University, Pullman, WA 99164, United States; Institute of Plant Science and Resources, Okayama University, 2-20-1 Chu-ou, Kurashiki, Okayama 710-0046, Japan; State Key Laboratory of Agricultural Microbiology, Huazhong Agricultural University, Wuhan 430070, Hubei Province, China; Hubei Key Laboratory of Plant Pathology, Huazhong Agricultural University, Wuhan 430070, Hubei Province, China; State Key Laboratory of Agricultural Microbiology, Huazhong Agricultural University, Wuhan 430070, Hubei Province, China; Hubei Key Laboratory of Plant Pathology, Huazhong Agricultural University, Wuhan 430070, Hubei Province, China

**Keywords:** virome, mycovirus, ecological adaptation, blackleg, thermal tolerance, *Brassica napus*

## Abstract

Despite extensive exploration of fungal viromes (mycoviromes), the ecological roles of mycoviruses remain poorly understood. Hence, we investigated the virome of *Leptosphaeria biglobosa* (an important fungal pathogen of rapeseed) from different geographic origins to determine the impacts of external factors on virome composition and their role in fungal ecological adaptation. The viromes of different *L*. *biglobosa* groups were investigated, and viral diversity correlated positively with field disease incidence and host diversity, but negatively with the altitude of the strain collection sites. A positive single-stranded RNA virus, namely, Leptosphaeria biglobosa letobirnavirus 1 (LbLV1), one of the core virome members (predominant viruses that constitute the majority of the viral community), has a significantly high incidence in *L*. *biglobosa* populations in winter rapeseed in southern China but a low incidence in *L*. *biglobosa* populations in spring rapeseed in northern China. Further laboratory and field tests revealed that LbLV1 could increase the ability of *L*. *biglobosa* to oversummer at average temperatures ranging from 23°C to 34°C in the winter rapeseed region of China. Therefore, the variation in LbLV1 incidence between winter and spring rapeseed should be a consequence of LbLV1-mediated adaptation to climate and cropping patterns. Furthermore, one gene, namely *Lbhsp12*, significantly induced by the hypothetical protein of LbLV1, is responsible for LbLV1-mediated thermal tolerance. Our findings indicate that mycovirome composition reflects environmental constraints, and core viruses can drive ecological adaptation by modulating host stress responses.

## Introduction

Virology has historically advanced through studies of viruses that are pathogenic to humans, animals, and plants. However, high-throughput sequencing (HTS) technology has facilitated the discovery of an increasing number of viruses infecting lower eukaryotes and prokaryotes in recent decades. The advancement of HTS revolutionized virology by dramatically broadening our knowledge of viral diversity and evolution and led to the creation of neo-virology [1, 2]. One of the main themes of neo-virology is understanding the as-yet-unexplored “Raison d’etre of non-pathogenic viruses”, in which mycoviruses or fungal viruses are among the key players. Although many mycovirome studies have revealed different virus incidence patterns in different host fungal populations, there is little to no information on their ecological functional roles. Most mycoviruses induce asymptomatic infections, whereas some cause phenotypic alterations, including hypovirulence (reduced virulence) or hypervirulence (enhanced virulence), in their host fungi, which are pathogenic to higher organisms [3–6]. Cryphonectria hypovirus 1 has been successfully used to control chestnut blight in Europe [7], whereas a double-stranded (ds) RNA virus increases the virulence of the mammal-pathogenic fungus *Talaromyces marneffei* [8]. Recently, some fungal viruses have been associated with the conversion of host fungi from pathogenic to endophytic lifestyles [9].

Oilseed rape, commonly known as canola (*B. napus* L.), is an important oil crop worldwide as a source of cooking oil, biofuel, and animal feed; it is second only to soybean in the oil market [10, 11]. Oilseed rape is widely grown in different regions of China and is divided into spring and winter oilseed rape, depending on the growing region [12]. In the winter oilseed rape region, located mainly in the Yangtze River Basin, plants are typically grown in Sichuan, Chongqing, Hubei, Hunan, Jiangxi, Anhui, and Jiangsu Provinces, which account for approximately 86.3% of the Chinese rapeseed yield. The spring oilseed rape region is located mainly in northern China, with a yield of approximately 7.4% [12], where plants grow in Qinghai, Xinjiang, Tibet, and Inner Mongolia. Blackleg, caused by the ascomycetous fungi *Leptosphaeria biglobosa* and *L*. *maculans*, is an economically important disease of oilseed rape and many cruciferous vegetables, with an average loss of 16 billion US dollars per year [13]. Blackleg or phoma stem canker is also widely distributed in all oilseed rape-growing regions of China; however, only one pathogen, *L. biglobosa*, has been detected [14]. To complete its life cycle and persist between seasons, *L*. *biglobosa* undergoes distinct survival phases in different cropping systems. In the winter oilseed rape region, the fungus must survive a four-month over-summering period following harvest (mid to late May), whereas in the spring oilseed rape region, it overwinters for approximately seven months after the September harvest. To understand the virome of *L. biglobosa*, we initiated a large-scale survey of fungal isolates all over China in 2018 and investigated the viral diversity in different *L. biglobosa* groups. During the study, we noticed a large difference in virome composition between fungal populations collected from the winter and spring oilseed rape regions.

In this study, we present evidence suggesting that (i) there is a positive correlation between virus diversity and disease incidence and a negative correlation between virus diversity and altitude; (ii) one core virome member, Leptosphaeria biglobosa letobirnavirus 1 (LbLV1)—named for its host genus *Leptosphaeria* (Le-to), its bipartite genome (bi), and its RNA nature (rna)—is significantly more common in winter rapeseed regions than in spring rapeseed regions; (iii) LbLV1 greatly contributes to the thermal tolerance of *L*. *biglobosa* in winter oilseed rape; and (iv) a fungal gene (*Lbhsp12*) encoding heat-shock protein 12 and a viral gene encoding a hypothetical protein are involved in the observed thermal tolerance of *L*. *biglobosa*.

## Materials and methods

### Fungal strains

**Figure 1:**
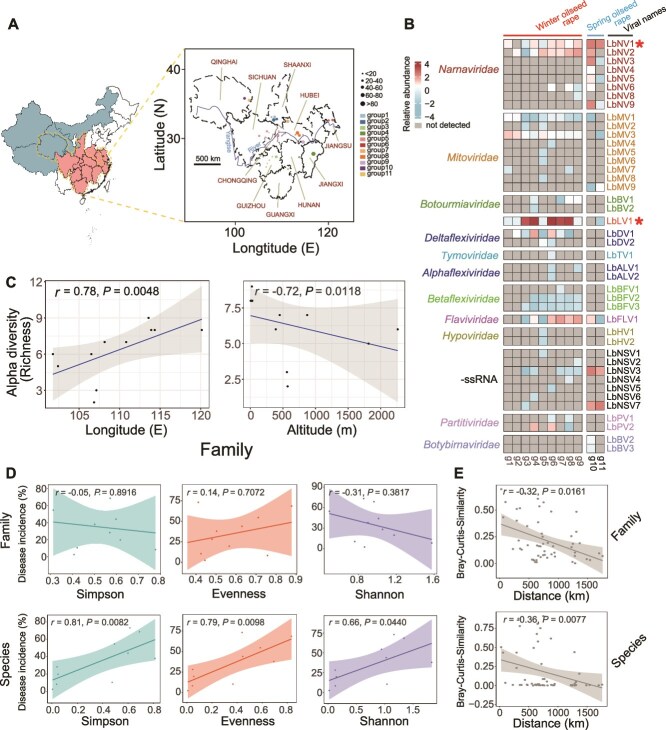
Virome composition and association analysis of viral diversity in different *Leptosphaeria biglobosa* groups. (A) Geographical origin of eleven *L. biglobosa* groups comprising 1103 strains. The positions of the dots with different colors correspond to the locations of the sampling sites. The dot size corresponds to the strain number, and the dot colors represent the different *L. biglobosa* groups. Geographic location of the spring (blue) and winter (red) oilseed rape growing regions in China. (B) Heatmap of virus species and relative abundance between different *L. biglobosa* groups (g1–g9: Strains collected from the winter oilseed rape region; g10–g11: Strains collected from the spring oilseed rape region). The cell color changes gradually from light blue to dark red, indicating increasing viral abundance, whereas a gray cell represents that no viral read was detected, and the two core virome members are indicated by “*.” (C) associations between viral family richness, longitude, and altitude. The *y*-axes refer to the viral richness index, whereas the *x*-axes refer to the longitude (E) and altitude (m). The fitted linear regression line with standardized *r* and *P* values is shown. (D) Associations between the viral Simpson, evenness, and Shannon indices (at the species and family levels) and field disease incidence. The *y*-axes refer to the field disease incidence of blackleg, whereas the *x*-axes refer to corresponding diversity indices. The fitted linear regression line with standardized *r* and *P* values is shown. (E) Distance–decay pattern of the virome in *L. biglobosa* at both the species and family levels. The *x*-axes refer to the spatial distance (km) between two fungal groups, whereas the *y*-axes refer to the viral Bray–Curtis similarity values between different groups. The fitted linear regression line with standardized *r* and *P* values is shown.

A total of 1103 strains were isolated from stalks of oilseed rape showing blackleg symptoms collected from 274 fields across nine provinces in China (Fig. 1A, Table S1). All the strains were grown on potato dextrose agar (PDA) at 20°C and confirmed to be *L*. *biglobosa* “brassicae” via PCR detection with species-specific primers and culture morphology in our previous study [15]. Next, cultures of the purified strains were stored on PDA slants at 4°C or at −80°C with 20% glycerine. For further RNA-sequencing analysis, these strains were divided into 11 groups, namely, groups 1 to 11 (g1–g11), according to geographical location and number of strains. The RNA sample used to establish the RNA-seq library in each group was generated by merging the total RNA of the group strains into a mixed pool.

### High-throughput sequencing and sequence analysis

To extract total RNA, each strain of *L*. *biglobosa* was cultured on a cellophane membrane overlaying a PDA plate for 7 days at 20°C. A mycelial mass of 1 g was collected from each strain and used for total RNA extraction using a TRIzol RNA extraction kit following the manufacturer’s instructions (Takara Bio, Inc., Japan). RNA quality was determined using an Agilent 2100 Bioanalyzer (Agilent Technologies, Germany) and quantified via a NanoDrop 2000 (Thermo Fisher Scientific, Germany) before library construction and sequencing. The RNA samples were stored at −80°C until use. Finally, 11 mixed pools were generated by mixing the RNAs of strains in each group and sending them to GENEWIZ (Suzhou, China) for RNA sequencing. Ribosomal RNA depletion (Ribo-Zero rRNA Removal Kit, Illumina, Inc.), library preparation (~1 μg RNA, TruSeq RNA Sample Preparation Kit, Illumina, Inc.), and HTS in a HiSeq X Ten System (Illumina, Inc.) were accomplished by GENEWIZ. The unqualified reads were filtered out, and then the clean reads were spliced from scratch using Trinity v2.3.3 [16]. Finally, the software Diamond v0.8.22 [17] and the nonredundant protein database in the National Center for Biotechnology Information (NCBI) (https://www.ncbi.nlm.nih.gov/) were used for BLASTX annotation, and the viral sequences were selected. The presence of each viral-like sequence was confirmed via RT–PCR with specific primers designed according to the corresponding sequences from pooled RNA samples representing each of the 11 groups. The database generated from each library was approximately 10–15 GB.

For the phylogenetic analysis, the Muscle program in MEGA X software was used for multiple sequence alignment of RNA-dependent RNA polymerase proteins (RdRps) with reference sequences or building neighbor-joining trees (Table S2) [18], and IQ-tree v1.6.0 [19] software was used to construct the maximum likelihood tree according to the retrieved alignments.

### Host genetic diversity analysis

The genomic DNA of 363 *L. biglobosa* strains from g1–g11 was extracted from mycelia with cetyltrimethylammonium bromide (CTAB) as previously described [15]. PCR detection was performed using three simple sequence repeat (SSR) primer pairs (Table S3). The amplified products were sent to Tianyihuayu Gene Technology (Wuhan, China) for sequencing using an ABI3730 sequencer (Thermo Fisher, Germany) and processed with GeneMarker v2.0 [20] to determine the size of the target fragment. The software PowerMarker v3.25 [21] was used to calculate the number of alleles observed (*Na*), the number of effective alleles (*Ne*), the Shannon Index (*I*), and Nei’s (1973) gene diversity index (*H*). Popgene v1.31 [22] software was used to calculate Nei’s genetic distance. A UPGMA tree was constructed for the g1–g11 populations on the basis of genetic distance.

### Viral diversity and its association analysis

According to the disease incidence data from the field survey in our earlier work [14], the disease incidence of each group was estimated using the average blackleg incidence of each sampling site. Each sampling point’s latitude, longitude, and altitude (m) data were determined via a global positioning system. The average values of the latitude, longitude, and altitude of the sampling points within the group were used for the association analysis. The geosphere v1.5–14 [23] R package was used to calculate the spatial distance matrix between groups according to latitude and longitude.

Salmon v1.9.0 [24] software (−quant parameter) was used to map sequence reads to their viral contigs. Virus relative abundance was then calculated as viral reads in per million reads divided by the length of the contigs (kb) and total reads in each library [25, 26]. All analyses were visualized in R (v4.1.0). The Vegan v2.6.2 [27] package was used to calculate the richness, Shannon, and evenness indices of the virome. Viral beta diversity was estimated using the Bray–Curtis similarity matrix in R (v4.1.0). Hmisc v4.7.0 [28] was used to evaluate the correlation and significance value between the diversity of the virome and other factors, such as the diversity of the host. Pairwise viral community similarities and geographic distance matrices were used for the correlation analysis (method: Spearman) in R (v4.1.0) to measure the distance–decay relationship (DDR).

### Virus particle purification and transmission electron microscope observation

A mycelial plug of *L. biglobosa* strain NN8–42 was grown at 20°C in shake medium at 150 rpm/min in the dark for 14 days in potato dextrose broth (PDB). Fungal mycelia were harvested by filtration with three layers of lens tissue. After harvest, 30 g of mycelia were ground to a fine powder in liquid nitrogen, and the resulting powder was subjected to extraction. Briefly, the powder was mixed with 100 mM phosphate buffer (PB; 8.0 mM Na_2_HPO_4_, 2.0 mM NaH_2_PO_4_, pH 7.0) and centrifuged at 12 096 × g at 4°C for 30 min to remove cellular debris. The supernatant was further ultracentrifuged (Optima LE-80 K; Beckman Coulter, Inc.) at 110000 × g at 4°C for 1 h to collect the sediment. The sediment was resuspended in 100 mM PB buffer and then overlaid on a centrifuge tube containing sucrose solutions with a concentration gradient ranging from 10%–50% (wt/vol) and centrifuged at 70 000 × g at 4°C for 2 h. After ultracentrifugation, each fraction was carefully collected, suspended in 0.05 M sodium phosphate buffer (pH 7.0), and individually tested for viral RNA by agarose gel electrophoresis. The purified virus particles were stained with phosphotungstic acid solution [20 g/liter (wt/vol), pH 7.4] and observed under a TEM (Hitachi HT7800/HT7700).

### Viral genomic RNA analysis and full-length cDNA cloning

A purified virus-like particle suspension (200 μl) was collected from each fraction after sucrose gradient centrifugation and treated with phenol/chloroform/isoamyl alcohol (25:24:1) (pH 5.2) to remove viral proteins. The nucleic acids were precipitated with ethanol, dissolved in DEPC-treated water, and analyzed by agarose gel electrophoresis. Briefly, 200 ng of total RNA aliquots were digested with 2 U of DNase I or S1 nuclease (TaKaRa) at 37°C for 1 h. Northern blotting of RNA1 and RNA2 was performed by lysing the viral particles to extract viral RNA as previously described [15].

The ssRNAs extracted from the viral particles were used for full-length cDNA cloning. The 5′- and 3′-terminal sequences of each ssRNA were cloned by ligating an RNA adaptor (Table S3) at the 5′- and 3′-termini and then reverse transcribed via sequence-specific primers. The cDNA strands were then used as templates for PCR amplification of the 5′- and 3′-terminal sequences with corresponding sequence-specific primers for each ssRNA segment. The intermediate cDNAs were amplified via RT–PCR with sequence-specific primer pairs. The cloning of the full-length sequence of the ssRNA was repeated three times. All these amplicons were detected by agarose gel electrophoresis, gel purified, cloned, inserted into *Escherichia coli* Trans1-T1, and sequenced.

### Virus transfection, transmission, and elimination

Protoplasts were prepared from actively growing mycelia (PDA, 20°C for 4 days) of *L. biglobosa* strain W10 as previously described [15]. Protoplasts (10^5^ cells/ml, 100 μl) were used for viral particle transfection via PEG 6000 as described by Deng *et al.* [15]. Following transfection, the protoplast suspensions were spread onto TB3 plates and grown at 20°C for 4 days. The resulting colonies were transferred to fresh PDA plates and subcultured three times before the detection of LbLV1 (Table S4).

*L. biglobosa* strains EB9–21 (LbLV1+) and NN8–42 (LbLV1+) were inoculated on PDA plates at 20°C for 10–14 days to determine the vertical transmission of LbLV1 through conidia. The conidia of each strain were washed with sterile water and filtered through triple-layer filter paper to obtain a conidial suspension. The conidial suspensions (10^3^, 10^4^, and 10^5^ conidia/ml, 50 μl) were spread on PDA plates and cultured at 20°C for 24 hours. Single germinated conidia (48 conidia per strain) were subsequently removed under a microscope and cultured on PDA plates at 20°C. Each single conidium strain was observed for colony morphology, and the presence of LbLV1 was detected via RT–PCR. LbLV1-free single conidium strains N42–16 and E21–13 were used for further biological property tests (Table S4).

### Transcriptome analysis and real-time semiquantitative PCR

Seven-day-old mycelia of *L. biglobosa* strains W10 and W10–9, grown at 20°C, were collected, and total RNA was extracted as described above. Mycelia collected from one plate served as a replicate, and each treatment had three replicates (Table S4). The RNA samples were sequenced on a HiSeq X Ten System (Illumina) at Novogene, Beijing, China. The raw reads were processed with Trimmomatic (v0.30) to remove adapters, PCR primers, fragments thereof, and bases with a quality score of less than 20. HISAT2 v2.2.0 [29] and HTSeq v2.0 [30] were used to map the clean reads from *L. biglobosa* to the reference sequence of *L. biglobosa* strain G12–14 (GCA_900465125.1). DESeq v1.32 [31], which employs a model based on the negative binomial distribution, was used to perform differential expression analysis. After the Benjamini and Hochberg approach was used to control the false discovery rate (FDR), *L. biglobosa* differentially expressed genes (DEGs) between strains W10 and W10–9 were identified according to the thresholds of |log2 Fold Change| ≥ 1 and *FDR* ≤ 0.05. DEG annotation and Gene Ontology (GO) functional enrichment analysis were performed with Blast2GO [32], the latter with a significant *P* value of less than 0.05. Kyoto Encyclopedia of Genes and Genomes (KEGG) annotation and enrichment were performed using the clusterProfiler Bioconductor package [33].

Several DEGs were selected for RT–qPCR to further confirm the reliability of the transcriptome analysis. The total RNA of strains W10 and W10–9 was extracted from mycelia cultured under the same conditions described above. cDNA synthesis was done via EasyScript One-Step gDNA Removal and cDNA Synthesis SuperMix (TransGen Biotech, China) with random primers according to the manufacturer’s instructions. RT–qPCR amplification was performed in a CFX96 Real-Time PCR Detection System (Bio-Rad) with iTaq universal SYBR Green Supermix (Bio-Rad). The actin gene of *L*. *biglobosa* (GenBank Acc. No. MH459178) was used as an internal control. All sequences of primers used for RT–qPCR are listed in Table S3.

### Sequence analysis of *Lbhsp12* and fungal transformation

A CD search was used to identify the conserved domains present in a protein sequence (https://www.ncbi.nlm.nih.gov/Structure/cdd/wrpsb.cgi). The sequences were aligned using the CLUSTALX program (version 2.1). The software PSIPRED (http://bioinf.cs.ucl.ac.uk/psipred/) was used for secondary structure prediction of proteins, and SWISS-MODEL (https://swissmodel.ExPASy.org/interactive) was used for 3-dimensional protein structure prediction. Genetic transformation, namely, *Lbhsp12*, was performed to verify the function of Hsp12 in *L. biglobosa*. The entire coding region of *Lbhsp12* (~300 bp) was amplified via PCR from the cDNA library of the total RNA of strain W10 with sequence-specific primers (Table S3). To generate the pKNTG-rp27-Hsp12-O overexpression plasmid, the amplified *Lbhsp12* coding region was inserted into the HindIII-linearized pKNTG-rp27 vector [34, 35] via homologous recombination using the ClonExpress II One Step Cloning Kit (Vazyme Biotech Co., Ltd.). The overexpression vector was subsequently cloned and inserted into *E. coli* Trans1-T1 and verified by sequencing and restriction enzyme digestion with *Hind* III. Finally, pKNTG-rp27-Hsp12-O was introduced into the protoplast (strain W10) via PEG3350-mediated transformation, positive transformants were selected, and the expression level of *Lbhsp12* was measured using RT–qPCR. The growth rates of two transformants (hsp12–6 and hsp12–7) were calculated at different temperatures and selected for microscopic analysis of hyphal structures. The ORF2-encoded hypothetical protein expression vector was constructed as described above.

### Biological property tests of the fungal strains

Colony morphology and mycelial growth rates were determined by inoculating 4-day-old mycelial plugs on PDA in triplicate at 15°C, 20°C, and 30°C and culturing them in the dark for 3–6 days. The stress tests were performed by inoculating mycelial plugs of *L. biglobosa* strains on PDA supplemented with 1 M D-sorbitol, 10 mM hydrogen peroxide, 0.1 mg/ml SDS, or 1 M sucrose and culturing them in the dark for 3–6 days. The mycelial growth rates of the fungal strains were determined as described previously [15]. The relative suppression rates of *L*. *biglobosa* under different treatments were calculated via the following formula: inhibition rate (%) = (AGR_ck_ − AGR_T_)/AGR_ck_ × 100, where AGR_ck_ represents the average mycelial growth rate on PDA, and AGR_T_ represents the average mycelial growth rate under the different stresses. The virulence of each strain was determined by inoculating mycelial plugs on living cotyledons of the oilseed rape (*B. napus* L.) cultivar “Zhongshuang No. 9”. The leaf lesions were measured and photographed at 6 days postinoculation (dpi).

### Assessment of fungal strain survival

To assess the survival of different *L. biglobosa* strains under thermal tolerance, the *L. biglobosa*-colonized stems of oilseed rape were placed in an incubator to simulate field conditions in the summer and in a field in Wuhan for over-summering. First, sterilized oilseed rape stems (16 cm) were inoculated with *L. biglobosa* strains W10 (LbLV1-) and W10–9 (LbLV1+), after which they were cultured at 20°C for 14 days until fungal colonies occupied the entire stem. For the laboratory test, six air-dried fungal colonized stems of each strain were placed in an incubator in the dark at 15°C (time: 20:00–5:00), 20°C (time: 5:00–10:00), 35°C (time: 10:00–17:00), and 20°C (time: 17:00–20:00) for 90 days. For the field test, six fungus-colonized stems were placed in the experimental field of Huazhong Agricultural University in Wuhan, China, from June 15 to August 15, 2022. For the laboratory test of overwintering, six air-dried fungal colonized stems for each strain were placed in a refrigerator at −20°C for 60 days.

After over-summering or overwintering, 14 plant tissues (3 mm × 3 mm) per stem were placed on PDA plates (seven plant tissues per plate) to observe the mycelial growth of *L. biglobosa* as an indicator of fungal survival. The survival rate of *L. biglobosa* in plant tissues from each stem served as a replicate, with six replicates per strain. To determine the survival rate of *L. biglobosa* conidia, 10 pycnidia on each stem were picked and transferred into a tube containing 100 μl PDB. After being squeezed with a stick, the conidia were released into the PDB and cultured at 20°C for 24 h. The germination rate of the conidia was determined by calculating the number of geminated conidia per 100 conidia at 24 h and 48 h. The survival rate of *L. biglobosa* conidia from each stem served as a replicate, with six replicates per strain. The germ tube length was determined by measuring the tube length of 50 randomly selected conidia for each strain.

### Quantification and statistical analysis

Descriptive statistics were determined, and χ^2^ tests, t tests, and analysis of variance were performed via R (v4.1.0), and *P* < 0.05 was considered to indicate significance. Figures were plotted with ggplot2 (v3.3.6).

## Results

### General viral composition of the *L. biglobosa* populations

After removing low-quality reads, clean reads ranging from 14 286 560 to 50 429 345 (raw reads: 14 836 013 to 56 406 106) (paired-end) were obtained from the libraries of 11 *L. biglobosa* groups (Fig. 1A). These reads were assembled de novo into large contigs. We analyzed the overall taxonomic profiles of the mycoviruses in the *L. biglobosa* population. The viromes across regions were predominantly positive-sense single-stranded RNA (+ssRNA) viruses, comprising up to 73.8% of all 42 putative viruses, whereas double-stranded RNA (dsRNA) viruses accounted for only 9.5%, and negative-stranded RNA (−ssRNA) genomes accounted for 16.7%. The viruses in the family *Narnaviridae* had the highest relative weights in those locations, representing up to 5.6% of the total viral loads (Fig. 1B; Table S5). As the sampling sites and strain numbers were not the same across the different groups, the effects of the two factors on viral composition were also evaluated, and the correlation analysis revealed that both factors had no significant effect on viral composition in each group (Fig. S1a).

Regarding the detection frequency of viruses, 18 viruses were detected only once in 11 groups, and 10 were detected in more than six groups. Two viruses, LbLV1 and Leptosphaeria biglobosa narnavirus 1 (LbNV1), accounted for 88.6% and 2.5% of the total viral abundance, respectively, whereas the remaining viruses accounted for only 8.9% of the total viral abundance. Thus, we defined LbLV1 and LbNV1 as the core virome members in the Chinese *L*. *biglobosa* population. Furthermore, only nine viruses were detected in *L*. *biglobosa* from both the winter and spring oilseed rape regions, indicating substantial differences in viral composition between them (Fig. 1B; Table S5).

### Characterization of viral families and species

In this study, 39 contigs appeared to represent 31 putative +ssRNA viruses (Table S6). Among them are 19 tentative members of the phylum *Lenarviricota*, including seven multisegmented narna-like splipalmiviruses (the proposed family Splipalmiviridae) [36, 37], one multisegmented narnaviruses, nine viruses belonging to the family *Mitoviridae*, and two belonging to the family *Botourmiaviridae* (Fig. S2 and S3). Detailed information is listed in Table S6. Phylogenetic tree was constructed using the neighbor-joining method for most of these sequences, revealing that they all belonged to *Mitoviridae*, *Narnaviridae*, or *Botourmiaviridae* (Fig. S4). In addition, we identified eight viruses belonging to the order *Tymovirales,* including two deltaflexiviruses, three betaflexiviruses, two alphaflexiviruses, and one tymo-like virus. The results of phylogenetic trees, conserved motifs and Blastp also supported that these viruses clustered with members of the order *Tymovirales* (Fig. S5a and 5B; Table S6). Two hypoviruses with partial genomes share 76% and 58% amino acid identifies with Setosphaeria turcica hypovirus 1 and Bipolaris oryzae hypovirus 1. The phylogenetic trees suggested that Leptosphaeria biglobosa hypovirus 1 (LbHV1) and LbHV2 belonged the gunus *Betahypovirus* and *Alphahypovirus*, respectively (Fig. S6). Additionally, one mycovirus namely Leptosphaeria biglobosa flavi-like virus 1 (LbFLV1) was clustered with members of the family *Flaviviridae* (Fig. S7). There were four dsRNA viruses, two belonging to the family *Partitiviridae* and two to the genus *Botybirnavirus* (Table S6). The results of blastp or phylogenetic trees also supported the classification results (Fig. S8; Table S6). Finally, five of seven –ssRNA viruses shared RdRp identities (29% to 78%) with members in the families *Mymonaviridae*, *Aspiviridae*, and *Discoviridae*. The phylogenetic analysis also revealed that they belong to the families *Mymonaviridae*, *Aspiviridae*, and *Discoviridae* (Fig. S9; Table S6). In addition, phylogenetic analysis revealed that some viruses formed independent branches, indicating that they may represent novel viral families (Fig. S9).

### Factors associated with viral diversity in *L. biglobosa* populations

To discern factors influencing viral α-diversity, we used a nonlinear model to estimate the relationships among latitude, the diversity of *L*. *biglobosa*, the incidence of blackleg disease, and viral diversity. The results revealed that viral richness was significantly positively correlated with longitude (*r* = 0.78, *P* < 0.01) but negatively correlated with altitude (*r* = −0.72, *P* < 0.05) and latitude (*r* = −0.62, *P* < 0.05) at the family level (Fig. 1C; Fig. S1b; Table S7). There is also a negative relationship between viral richness and altitude at the species level (*P* < 0.05) (Fig. S1b). Moreover, we calculated the genetic diversity index and population genetic distances of *L*. *biglobosa* based on the SSR markers (Fig. S10; Fig. S11a). The viral richness was significantly positively correlated with the diversity index (*Ne* and *H*) of *L*. *biglobosa* at the family level (*P* < 0.05) but was only correlated with the *Ne* index at the species level (*P* < 0.05) (Fig. S11b; Table S7), suggesting that a more diverse fungal population may harbor more diverse mycoviruses. In addition, the field disease incidence of blackleg was significantly correlated with the α-diversity (Evenness, Shannon, and Simpson indices) of the virome at the species level (*P* < 0.05) (Fig. 1D; Table S7).

The DDR is a comprehensive measurement of biogeography for communities from all domains of life; thus, we tested whether it also applies to fungal viral communities. The results revealed significant declines in mycoviral community similarity with increasing geographic distance at both the family and species levels (*P* < 0.05) (Fig. 1E; Tables S7 and S8). Finally, an eco-evolutionary relationship was observed according to host genetic distance and the Bray–Curtis similarity of the virome, suggesting that the virome composition could be closely related to host population evolution (Fig. S1c; Table S8 and S9).

### Asymmetric distribution and molecular characterization of the core virome member LbLV1

**Figure 2:**
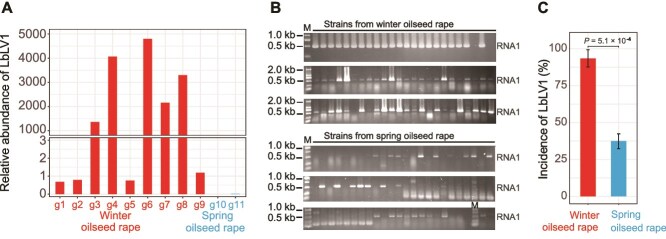
Natural incidence of Leptosphaeria biglobosa letobirnavirus 1 (LbLV1) in *L. biglobosa* populations from spring and winter oilseed rape regions. (A) Relative abundance of LbLV1 in different *L. biglobosa* groups, where g1–g9 were *L. biglobosa* populations from the winter oilseed growing region, and g10–g11 were *L. biglobosa* populations from the spring oilseed growing region. (B) RT–PCR detection of RNA1 of LbLV1 in *L. biglobosa* strains from spring and winter oilseed rape regions. Please note that the target band is approximately 500 bp in length. (C) Incidence of LbLV1 in *L. biglobosa* populations from spring and winter oilseed rape regions.

As mentioned above, the virus detection patterns of the analyzed fungal populations were associated with disease incidence, latitude, the diversity of *L*. *biglobosa*, and rapeseed cropping types. Additionally, samples taken from 9 locations in the winter oilseed rape region presented high LbLV1 loads (Fig. 1A and 2A, Table S5). The virome profiles revealed that the average relative abundance of LbLV1 in the spring oilseed rape region (0.009737) was significantly lower than that in the winter oilseed rape region (1743.51) (Table S5). To further confirm the asymmetric distribution of LbLV1 in different oilseed rape regions, *L*. *biglobosa* populations from the two regions were subjected to RT–PCR. The results revealed that the incidence of the *L*. *biglobosa* population carrying LbLV1 in the winter oilseed rape region (93.1%) was significantly greater than that in the spring oilseed rape region (37.7%) (Fig. 2B and C).

**Figure 3:**
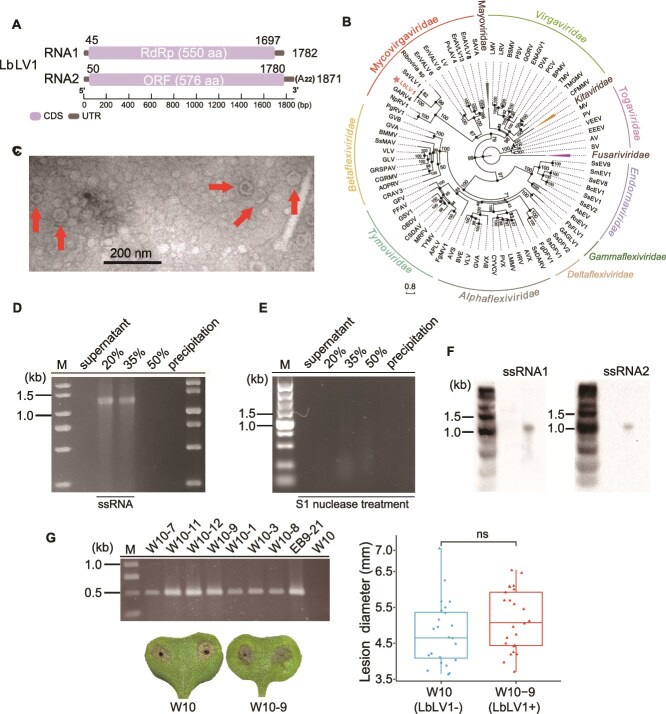
Characterization of Leptosphaeria biglobosa letobirnavirus 1 (LbLV1). (A) Schematic diagram of the genetic organization of LbLV1. RNA1 is 1782 nt long and comprises one large ORF, designated ORF1, which encodes a putative RNA-dependent RNA polymerase (RdRp). RNA2 is 1871 nt long and comprises one large ORF, designated ORF2, which encodes a hypothetical protein. (B) Phylogenetic analysis of the RNA-dependent RNA polymerase (RdRp) region of LbLV1 and selected RNA viruses of *Alphaflexiviridae*, *Betaflexiviridae*, *Deltaflexiviridae*, *virgaviridae*, *Endornaviridae*, *Gammaflexiviridae*, *Togaviridae*, *Tymoviridae*, and *Mycovirgaviridae* (the proposed family). Amino acid sequences of RdRps were aligned with muscle, and the phylogeny was generated via the maximum likelihood method in IQ-tree. The numbers at the nodes indicate the bootstrap values out of 1000 repetitions. (C) TEM images of the virus particles (~30 nm) of LbLV1. Arrowheads indicate the viral particles. (D) Agarose gel (1%) electrophoresis analysis of the viral RNA extracted from purified LbLV1 virus particles. Lanes: Supernatant, 20%, 35%, 50%, and precipitation represent the different fractions in graded centrifuges of sucrose solutions. Please note that the apparently smaller-than-expected RNA sizes result from using double-stranded DNA markers. Viral RNAs are single-stranded and therefore migrate faster than double-stranded DNA molecules of equivalent length. (E) Agarose gel (1%) electrophoresis analysis of the viral RNA after digestion with the S1 nuclease. (F) Northern blotting detection of the viral RNA extracted from purified LbLV1 virus particles. (G) RT–PCR detection of LbLV1 RNA1 in strain W10 (LbLV1-) and its derivative strains (LbLV1+), of which strains W10–1, W10–3, W10–8, and W10–9 were generated through LbLV1 transfection, whereas strains W10–7, W10–11, and W10–12 were generated through horizontal transmission of viruses. Strain EB9–21 served as a positive control. Pathogenicity assay (23°C, 7 days) and lesion diameter of *L*. *biglobosa* strains W10 and W10–9 on cotyledons of oilseed rape (ns, not significantly different, *P* > 0.05). The symbols “+” and “–” indicate the presence and absence of LbLV1, respectively.

We hypothesize that LbLV1 assisted in the over-summering of *L*. *biglobosa* after rapeseed harvest, as over-summering is essential for the survival and epidemic of *L*. *biglobosa* in the winter oilseed rape region. It was anticipated that one of the core viruses of the virome in Chinese *L*. *biglobosa* populations, LbLV1, contributed to the observed association, as LbLV1 was abundantly and frequently detected in many of the fungal populations collected from winter rapeseed regions. To test this hypothesis, after molecular characterization, we examined the effects of LbLV1 on fungal hosts under laboratory conditions mimicking field conditions. The full-length genome of LbLV1 possesses two ssRNA strands, namely, RNA1 (1782 nt in length, GenBank Acc. No. OQ650196) and RNA2 (1871 nt in length, GenBank Acc. No. OQ650197). The sequence analysis of RNA1 revealed that it possesses an ORF (ORF1) putatively encoding the viral RdRp (550 amino acid residues) with a predicted molecular mass of 62 kDa (Fig. S12a). In addition, RNA2 possesses an ORF (ORF2) encoding a hypothetical protein (HP, 576 amino acid residues) with a predicted molecular mass of 64 kDa (Fig. S12b). The 5′-untranslated region of the coding strand of RNA1 was 62 nt, whereas that of RNA2 was 70 nt. The nucleotide sequences at the 5′-termini of RNA1 and RNA2 shared a sequence identity of 64%. A poly-A tail (22 nt) was detected at the 3′-terminal sequence of RNA2 (Fig. 3A). BLASTP searches revealed that the two putative proteins encoded by LbLV1 are identical to the two proteins encoded by Sclerotinia sclerotium virga-like virus 1, with sequence (aa) identities of 97% for RNA1 and 96% for RNA2. Moreover, the ORF1-encoded protein also showed similarities to the RdRps of grapevine-associated RNA virus 4 (97%) and Erysiphe necator associated abispo virus 8 (97%) (Table S6). The phylogenetic analysis conducted on viral RdRps revealed that LbLV1 initially formed an independent clade with Grapevine-associated RNA virus 4 and Sclerotinia sclerotiorum virga-like virus 1, exhibiting a bootstrap value of 99%. Subsequently, it clustered with members of the virga-like viruses, predominantly identified in fungi, forming a larger clade with a bootstrap value of 67% (Fig. 3B). Electron microscopy revealed that the negatively stained viral particles purified from strain NN8–42 were isometric, with a diameter of ~30 nm (Fig. 3C). The viral RNA extracted from the viral particles was detected as a band at ~1.5 kb in a 1% agarose gel, which could be degraded with S1 nuclease (Fig. 3D and E), suggesting its ssRNA property. In addition, the identity of LbLV1 genomic RNA was confirmed by both RT–PCR and northern blotting (Fig. 3F).

### LbLV1 enhanced stress tolerance in *L*. *biglobosa in vitro*

**Figure 4:**
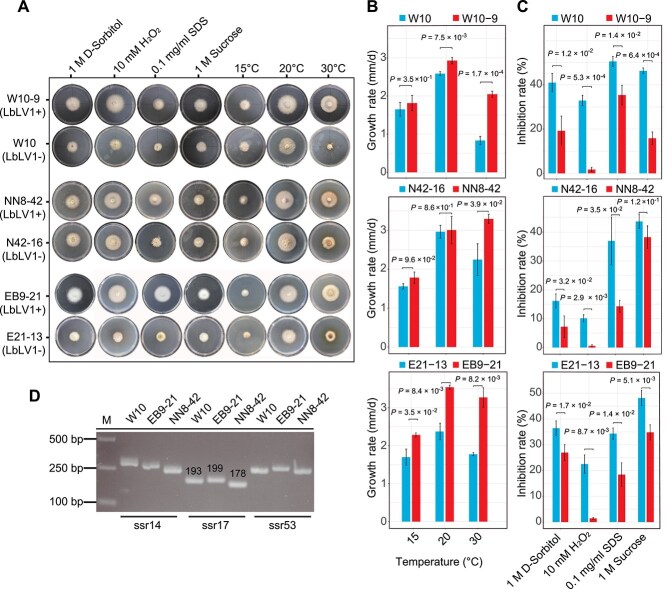
Response of *Leptosphaeria biglobosa* strains to abiotic stresses. (A) Seven-day-old colony morphology of different *L*. *biglobosa* strains under four abiotic stresses (D-Sorbital, H_2_O_2_, SDS and sucrose) at 20°C and on PDA plates at 15°C, 20°C and 30°C. The symbols “+” and “–” indicate the presence and absence of LbLV1, respectively. (B) Radial mycelial growth rates of different *L. biglobosa* strains at 15°C, 20°C, and 30°C. The *x*-axes refer to different temperatures, whereas the *y*-axes refer to the mycelial growth rate (mm/d), with standardized *P* values. (C) The inhibition of radial mycelial growth of different *L. biglobosa* strains under four abiotic stresses. The *x*-axes refer to different abiotic stresses, whereas the *y*-axes refer to the relative suppression rate (%), with standardized *P* values. (D) SSR analysis of strains W10, NN8–42, and EB9–21. Ssr14, ss17, and ssr53 are three different SSR sequences. The numbers on the bands generated by ssr17 indicate the lengths of the nucleotides. The three strains presented different genetic backgrounds according to the SSR markers.

To investigate the role of LbLV1 role in *L*. *biglobosa* biology, LbLV1 viral particles were introduced into an LbLV1-free strain (strain W10) to prepare an isogenic pair of LbLV1-free and LbLV1-infected strains (Fig. 3G; Table S4). The transmission of LbLV1 through both hyphal anastomosis and artificial transfection was successful. Among the four derivative strains, strain W10–9 was selected for further testing because it consistently exhibited a strong and stable PCR amplification signal, indicating a high and stable viral load. The introduction of LbLV1 had no significant effect on the virulence of *L*. *biglobosa*, as the average lesion diameter on living cotyledons of oilseed rape caused by strain W10 (4.76 mm) was similar to that caused by strain W10–9 (5.16 mm) (Fig. 3G). To investigate the effects of LbLV1 on other biological properties of *L*. *biglobosa*, strain W10–9 was compared with its progenitor W10 for its growth rates at different temperatures (15°C, 20°C, and 30°C) or under different abiotic stresses (1 M sorbitol, 1 M sucrose, 0.1 g/L SDS, and 10 mM hydrogen peroxide) (Fig. 4A). Compared with that of W10 (2.6 mm/day, 20°C; 0.83 mm/day, 30°C), the mycelial growth of W10–9 (2.9 mm/day, 20°C; 2.0 mm/day, 30°C) was greater at both 20°C and 30°C, especially at 30°C (Fig. 4A and B). Additionally, the relative inhibition rates of strain W10–9 were significantly lower than those of W10 under all abiotic stress conditions, indicating the significantly increased tolerance of strain W10–9 to abiotic stress (Fig. 4C). Moreover, similar phenomena were also observed in the other two *L*. *biglobosa* strains (EB9–21 and NN8–42), which have different genetic backgrounds characterized by distinct SSR markers (Fig. 4D), both carrying LbLV1. These strains exhibited significantly enhanced mycelial growth at 30°C and increased stress tolerance compared to their LbLV1-free derivatives, E21–13 and N42–16 (Fig. 4A–C). These results indicate that LbLV1 confers abiotic stress tolerance to *L. biglobosa* strains with different genetic backgrounds.

### Contribution of LbLV1 to *L*. *biglobosa* over-summering under laboratory and field conditions

**Figure 5:**
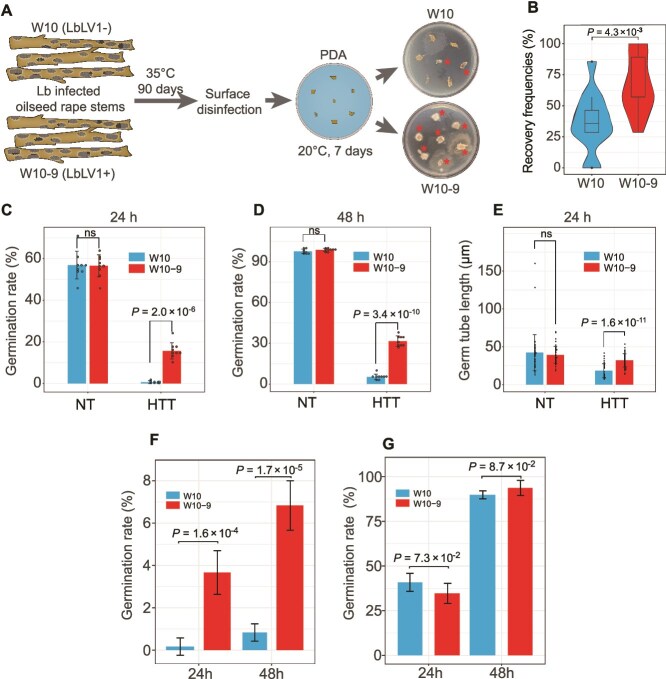
Effect of Leptosphaeria biglobosa letobirnavirus 1 (LbLV1) on over-summering under indoor and field conditions. (A) Experimental process of the indoor over-summering test and recovery of colonies of strains W10 and W10–9 on PDA isolated from diseased stubbles of oilseed rape. (B) Recovery frequencies of *L. biglobosa* strains W10 and W10–9 from stubbles of oilseed rape. The colonies of *L. biglobosa* are indicated by “*.” conidial germination rates of *L. biglobo*sa strains W10 and W10–9 on PDA at 20°C at 24 h (C) and 48 h (D) and their corresponding germ tube lengths at 24 h (E), with standardized *P* values, and ns represents *P* > 0.05. HTT and NT represent indoor over-summering (high-temperature, 35°C) treatment and nontreatment (20°C), respectively. Conidial germination rates (20°C) at 24 h and 48 h on PDA after field over-summering (F) or indoor overwintering treatment (G). Strain W10 was LbLV1 free (LbLV1–), whereas strain W10–9 was infected with LbLV1 (LbLV1+).

We simulated over-summering conditions in an incubator and tested the survival of *L*. *biglobosa* under these conditions. After being incubated for 3 months, the percentage of *L. biglobosa* reisolated from W10–9-inoculated oilseed rape stems (LbLV1-infected, 68%) was significantly greater than that from W10-inoculated oilseed rape stems (LbLV1-free, 37%) (Fig. 5A and B). Moreover, compared with those of W10-inoculated stems, the germination rates (24 and 48 h) and germ tube lengths of conidia from W10–9-inoculated stems also greatly increased (Fig. 5C–E, Fig. S13). In addition, we confirmed the presence of LbLV1 in derivative strains reisolated from W10–9-inoculated stems. In contrast, no significant differences in the germination rate (24 and 48 h) or germ tube length were observed between strains W10 and W10–9 without treatment (Fig. 5C–E, Fig. S13). Similarly, the field over-summering tests revealed that LbLV1 significantly increased the overall survival of *L. biglobosa*, consistent with the laboratory results (Fig. 5F, Fig. S13). However, no significant differences in the germination rate (24 and 48 h) were observed between strains W10 and W10–9 after the indoor overwintering treatment (Fig. 5G, Fig. S13). Finally, LbLV1 could be efficiently transmitted to either asexual (87.5%) or sexual (75%) offspring of *L. biglobosa* through conidium or ascospore (single-ascospore strains collected from 2017–2019), respectively (Fig. S14a and b). These results suggest that LbLV1 enhances the potential of the host fungus to oversummer but not overwinter.

### Gene expression profile associated with LbLV1-induced stress tolerance in *L*. *biglobosa*

**Figure 6:**
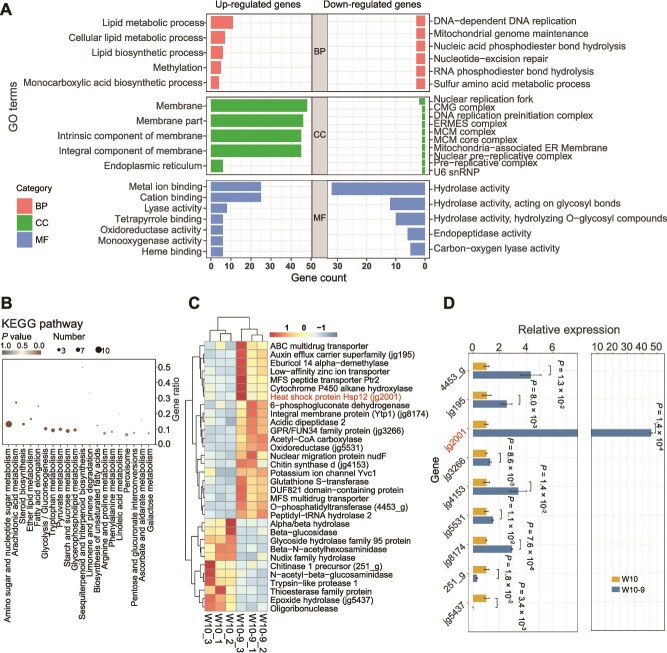
Differential gene expression in *L. biglobosa* induced by Leptosphaeria biglobosa letobirnavirus 1 (LbLV1) infection. (A) Top 38 GO analysis of genes upregulated and downregulated by LbLV1. The colors in the outermost circle represent the relative enrichment of genes for three biological processes represented by MF (molecular function), CC (cellular component), and BP (biological process). The inner circle has various colors, lengths, and line widths; accordingly, the number of DEGs enriched in specific biological processes is shown. (B) Top 20 KEGG pathways enriched among genes upregulated by LbLV1. (C) Heatmap of gene expression related to abiotic stress. The genes labeled with gene IDs in parentheses were verified by RT–qPCR. (D) Verification of the expression of partial genes related to abiotic stress via RT–qPCR.

To further investigate the underlying mechanisms responsible for LbLV1-induced stress tolerance in *L*. *biglobosa*, RNA-seq was used to test the changes in gene expression patterns in strain W10–9 compared with those in strain W10. The results showed that LbLV1 can manipulate the gene expression pattern of *L. biglobosa*. Among the DEGs, 601 were upregulated, and 614 were downregulated at least 2-fold. The GO enrichment analysis revealed that more upregulated genes in the LbLV1-infected strain W10–9 were enriched in cellular components of membranes, including genes that encode components of integral components of membranes, intrinsic components of membranes, membrane parts, lipid metabolic processes, and lipid biosynthetic processes. These DEGs encode key proteins that play critical roles in growth and resistance to abiotic stress. Moreover, the expression levels of several other genes related to the stress response, such as small heat shock proteins (HSPs), glutathione transferases, and transporters, were also significantly increased. In contrast, most downregulated genes were enriched in hydrolase activity (Fig. 6A and C; Table S10).

A total of 71 KEGG pathways were enriched in the upregulated DEGs, including amino sugar and nucleotide sugar metabolism (10 DEGs), tryptophan metabolism (6 DEGs), starch and sucrose metabolism (6 DEGs), steroid biosynthesis (4 DEGs), and glycolysis/gluconeogenesis (5 DEGs). The downregulated DEGs were enriched in 75 KEGG pathways, including amino sugar and nucleotide sugar metabolism (12 DEGs), cysteine and methionine metabolism (6 DEGs), fructose and mannose metabolism (5 DEGs), tyrosine metabolism (5 DEGs), cyanoamino acid metabolism (4 DEGs), and other glycan degradation (5 DEGs). Two genes, *Erg1p* (encoding squalence epoxidase) and *Erg3p* (encoding C-5 sterol desaturase), demonstrated upregulated expression in strain W10–9, indicating increased sterol biosynthesis. Furthermore, upregulated expression of catalase genes was observed, indicating that LbLV1 infection promoted the hydrolysis of hydrogen peroxide in *L. biglobosa* (Fig. 6B; Table S10). In addition, the expression of some of these upregulated genes was confirmed via RT–qPCR (Fig. 6D).

### Overexpression of *Lbhsp12* and LbLV1 HP increased the thermal tolerance of *L. biglobosa*

**Figure 7:**
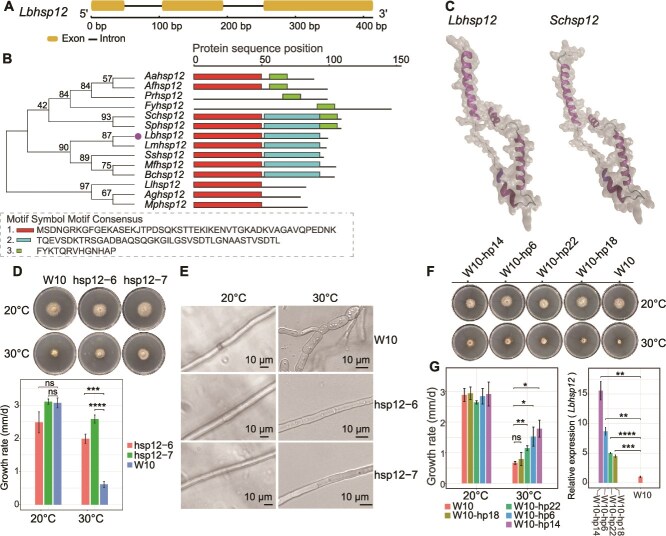
Molecular characterization of Leptosphaeria biglobosa letobirnavirus 1 (LbLV1)-mediated thermal tolerance. (A) Gene structure of *Lbhsp12*; the yellow rectangle represents the exon region, whereas the black line represents the intron region. (B) Phylogenetic analysis and conserved motifs of *Lbhsp12* in different fungal species. Red, blue, and green represent the three conserved motifs. (C) Predicted protein structures of *Lbhsp12* of *L. biglobosa* and *Schsp12* of *Saccharomyces cerevisiae*. (D) Colony morphology (upper, 20°C and 30°C, 5 days) and growth rates (lower) of *L. biglobosa* strain W10 and its two *Lbhsp12*-overexpressing transformants (hsp12–6 and hsp12–7) on PDA (*** and ****, representing *P* < 0.001 and 0.0001, respectively; ns represents *P* > 0.05). (E) Hyphal morphology (20°C and 30°C, 7 days) of strain W10 and its two *Lbhsp12*-overexpressing transformants (hsp12–6 and hsp12–7) on PDA. (F) Colony morphology (20°C and 30°C, 5 days) of *L. biglobosa* strain W10 and its four transformants (W10-hp6, W10-hp14, W10-hp18, W10-hp22) with expression of the LbLV1 ORF2-encoded protein on PDA. (G) Growth rates (at 20°C and 30°C, 5 days) and relative expression of *LbHsp12* (20°C, 5 days) in *L. biglobosa* strain W10 and its four transformants (W10-hp6, W10-hp14, W10-hp18, W10-hp22) on PDA (**, *** and ****, representing *P* < 0.01, *P* < 0.001, and 0.0001, respectively; ns represents *P* > 0.05).

Among the upregulated genes, the expression of one HSP gene, designated *Lbhsp12*, the homolog (41% aa identity) of *Schsp12*, which confers thermal tolerance in *Saccharomyces cerevisiae* (Fig. 7A and B), was upregulated approximately 46-fold according to RT–qPCR analysis (Fig. 6D). Phylogenetic and conserved domain analyses revealed that Hsp12 genes are highly conserved in ascomycetes. Furthermore, structure prediction (root mean square deviation (RMSD) = 0.11) revealed four α-helices in the two HSP genes (*Lbhsp12* and *Schsp12*), suggesting that they have very similar structures (Fig. 7C). To test whether *Lbhsp12* contributes to LbLV1-induced thermal tolerance, *Lbhsp12* was overexpressed in strain W10 (Fig. S15a), resulting in the generation of strains hsp12–6 and hsp12–7. No significant difference in mycelial growth at 20°C was detected between strain W10 and two hsp12-overexpressing transformants (strains hsp12–6 and hsp12–7). However, strains hsp12–6 and hsp12–7 grew significantly faster on PDA than did strain W10 at 30°C. Colonies of strain W10 were small with irregular margins, whereas of strains hsp12–6 and hsp12–7 grew normally (Fig. 7D). In addition, microscopic observation revealed that the hyphal cells of strain W10 became swollen at 30°C, whereas the hyphal cell morphology of strains hsp12–6 and hsp12–7 was still normal at 30°C (Fig. 7E).

cDNA of the LbLV1 ORF2 HP coding domain was transformed into strain W10, and its expression in different transformants was confirmed via RT–PCR (Fig. S15b). The mycelial growth rates of the four transformants (W10-hp18, W10-hp22, W10-hp6, and W10-hp14) were similar to that of strain W10 at 20°C (*P* > 0.05). However, at 30°C, three transformants grew significantly faster (1.2–1.8 mm/d) than did strain W10 (0.67 mm/d) (*P* < 0.05), and the expression of *Lbhsp12* in all four transformants was also triggered by 4.5- to 15.6-fold in comparison with that in strain W10 (Fig. 7F and G).

## Discussion

Plant fungal diseases have long been considered related to three components represented by the disease triangle: susceptible plants, virulent pathogens, and favorable environmental conditions [38]. Among the critical environmental factors, a suitable temperature is essential for the epidemic of plant fungal diseases. With the recent development of sequencing technology, most attention has focused on the molecular and genetic basis of plant–pathogen interactions. Additional indirect factors, such as the fungal virome, affecting plant disease epidemics under field conditions, have received considerably less attention. Although many mycoviral sequences from phytopathogenic fungi have been reported recently [39–41], their ecological role in the epidemic of plant diseases remains unclear. In this study, a relatively low number of viruses were identified in *L*. *biglobosa* populations. This finding aligns with our previous research, which showed that fewer viruses were detected in *L*. *biglobosa* isolates compared to *B. cinerea* isolates, despite both groups having the same number of strains and originating from similar sources [15]. Therefore, the relatively low viral diversity may be influenced by the biological characteristics of the host fungus, such as its reproductive mode in nature and its host plant range. Additionally, we found that one core virome member, LbLV1, of an important fungal pathogen (*L. biglobosa*) of rapeseed could increase the thermal tolerance of its host fungus, shaping the adaptation of *L. biglobosa* to the cropping pattern in the winter oilseed rape region of China. This conclusion is supported by meta-analyses and individual biological investigations under both laboratory and field conditions.

LbLV1 was detected more frequently in *L*. *biglobosa* populations from the winter oilseed rape region compared to those from the spring oilseed rape region, as confirmed by HTS and RT–PCR. Winter oilseed rape is primarily cultivated in southern China, particularly in the Yangtze River Basin, and is harvested from mid to late May. In contrast, spring oilseed rape, grown in northern China, is harvested in September. After harvest, *L*. *biglobosa* populations on spring oilseed rape overwinter for approximately seven months, with average temperatures ranging from −14°C to 19°C. In comparison, those on winter oilseed rape oversummer for about four months, experiencing temperatures between 23°C and 34°C. The significant temperature differences post-harvest, along with the higher incidence of LbLV1 in the winter oilseed rape region, suggest that LbLV1 may positively influence the over-summering of *L*. *biglobosa*.

**Figure 8:**
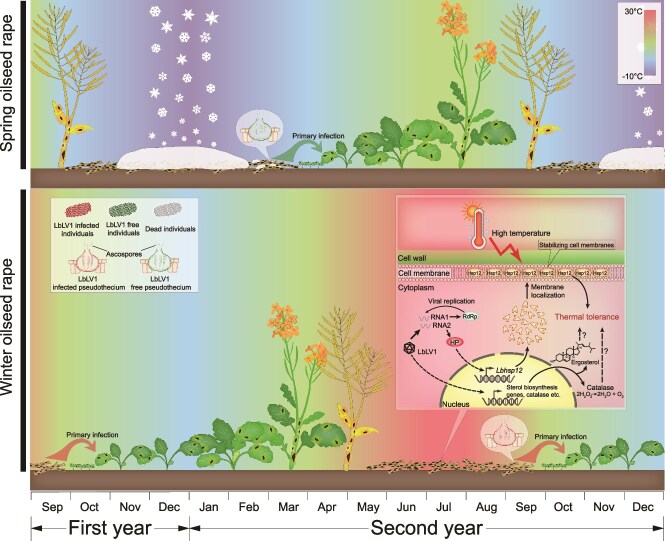
Leptosphaeria biglobosa letobirnavirus 1 (LbLV1) shapes the adaptation of *L*. *biglobosa* to the climate of the winter oilseed rape region and its underlying mechanisms. The times at which oilseed rape is planted and harvested differ substantially between spring and winter oilseed rape-growing regions. The spring oilseed rape is planted in April and harvested in September, whereas the winter oilseed rape is planted in October and harvested in May of the second year. Thus, *L. biglobosa* populations on plant remnants need to overwinter in the spring oilseed rape region but oversummer in the winter oilseed rape region. The monthly average temperatures in the two regions are indicated by colors in the figure’s background; dark blue represents low temperatures, whereas red indicates high temperatures. Compared with LbLV1-free individuals (green color), *L. biglobosa* individuals carrying LbLV1 (red color) are more likely to survive after over-summering. Consequently, the incidence of LbLV1 in the winter oilseed rape region is much greater than that in the spring oilseed rape region. In infected hyphal cells, the hypothetical protein encoded by LbLV1 RNA2 can increase the expression of *Lbhsp12* approximately 45-fold. The Lbhsp12 protein is translocated to the cell membrane, where it can stabilize the membrane structure at high temperatures. Moreover, LbLV1 infection can also increase the expression of genes related to sterol biosynthesis and catalases, which may also contribute to the thermal tolerance of *L. biglobosa* individuals harboring LbLV1.

The speculation that LbLV1 may facilitate the over-summering of *L*. *biglobosa* is supported by both laboratory and field tests. First, LbLV1 enhances the fungal tolerance to thermal stress in strains with varying genetic backgrounds. Secondly, the results from both laboratory and field over-summering tests fully met our expectations. The fungus was more readily reisolated from the W10–9 (LbLV1+) infected oilseed rape than from the W10 (LbLV1–) infected oilseed rape (Fig. 5B), accompanied by a higher germination rate and longer germ tube length in strain W10–9. These data strongly support the conclusion that LbLV1 can assist *L*. *biglobosa* in surviving during over-summering (Fig. 8). Additionally, the survival rates of the LbLV1-free and LbLV1-infected *L*. *biglobosa* strains did not significantly differ between the indoor overwintering treatment groups (−20°C, 60 days) (Fig. 5G). Therefore, our hypothesis is that the high incidence of LbLV1 in the winter oilseed rape region is a consequence of long-term virus–host coevolution and natural selection. The results strongly support that LbLV1 confers tolerance to the fungus to survive at high temperatures, and suggest that LbLV1 is essential for the successful over-summering of *L. biglobosa* in the winter oilseed rape region but may be inconsequential in the spring oilseed rape region (Fig. 8). Therefore, our hypothesis is that LbLV1, a member of the core virome of *L*. *biglobosa*, shapes the adaptation of *L*. *biglobosa* to the cropping pattern of winter oilseed rape and to the climate in the Yangtze River Basin. Similarly, the fungal endophyte *Curvularia protuberata* is infected by the Curvularia thermal tolerance virus, which enhances the thermal tolerance of panic grass (*Dichanthelium lanuginosum*) [42].

Plant viruses can increase the tolerance of their hosts under extreme conditions [43, 44]; however, the underlying mechanism remains unclear. To further investigate the underlying mechanisms responsible for LbLV1-mediated thermal tolerance, RNA-seq was used to compare the transcriptomes of LbLV1-infected and LbLV1-free strains. The results revealed that LbLV1 induced the upregulation of the expression of many genes related to the abiotic stress response, including transporters, fatty acid and chitin synthesis genes, and oxidoreductases (Table S10). Among the genes with upregulated expression, a small HSP, *LbHsp12*, was highly induced by LbLV1 infection. This group of proteins is conserved among different fungi and has been reported to be associated with fungal tolerance to many abiotic stresses, including hydrogen peroxide and heat [45, 46]. The overexpression of *LbHsp12* significantly increased the thermal tolerance of *LbHsp12* transformants. Moreover, the expression of the HP of LbLV1 in *L*. *biglobosa* could increase the thermal tolerance of the transformants to varying degrees, accompanied by the upregulation of *LbHsp12* expression. We speculate that this HP could be the viral capsid protein. However, we did not observe distinct bands corresponding to the capsid protein suitable for mass spectrometry analysis. Therefore, further investigation is required to clarify this issue. Furthermore, the expression level of *Lbhsp12* was positively correlated with the growth rate of *L*. *biglobosa* at 30°C, and a lower level of *Lbhsp12* overexpression (4.5-fold, strain W10-hp18) was not able to significantly increase growth at 30°C. These data suggest that LbLV1-mediated thermal tolerance in *L*. *biglobosa* is triggered by HP, inducing the overexpression of *LbHsp12* (Fig. 7). The function of *Hsp12* is associated with plasma membrane stabilization in *Candida albicans* [47]. In *Cryptococcus gattii*, disruption of *HSP12.1* results in increased sensitivity to the cell membrane stressor SDS [48]. In *Ustilago maydis*, *Hsp12* interacts with lipid membranes, thereby enhancing the stability of lipid vesicles [49]. In *S. cerevisiae*, *Hsp12* enhances thermal tolerance by stabilizing the cell membrane [46], and a similar protein structure (four α-helices) was also predicted in *Lbhsp12*. Therefore, we propose that *Lbhsp12* may also localize to cell membranes under heat stress, increasing thermal tolerance through membrane stabilization (Figs. 7 and Fig. 8).

With the development of HTS, many viral sequences have been identified in animals, plants, bacteria, and fungi [39–41, 50–53]. These viral data greatly broaden our knowledge of viral diversity and evolution, but they also raise the question: “What are the ecological functions of the mycovirome?”. It has long been believed that most mycoviral infections are asymptomatic, and the diverse mycoviruses identified by HTS further enforced this conception. However, the present study suggests that asymptomatic mycoviruses play a crucial role in fungal ecological adaptation. Furthermore, the fungal virome composition was also correlated with many other factors, including altitude, disease incidence, and host genetic diversity in the present study. Although viral richness was also positively correlated with longitude at the family level, this observation is due mainly to altitude, as one feature of the Chinese topography is that the altitude is high in the west and low in the east. Species diversity, including that of plants [54, 55] and fungi [56, 57], decreases with increasing altitude due to the extreme environmental conditions at high altitudes. We suppose that lower fungal diversity in high-altitude oilseed rape-growing regions, such as Qinghai, limits the acquisition of viruses from other fungal species of *L*. *biglobosa* through interspecies direct horizontal transmission via mycelial contact [58–60] and/or indirect transmission, which is likely mediated by other organisms [61]. In addition, the three viral diversity indices (Simpson, Evenness, and Shannon) showed a significant positive correlation with field disease incidence at both the species and family levels. Our hypothesis is that high disease incidence increases the possibility of virome exchange within *L*. *biglobosa* populations and other fungal groups because high disease incidence often corresponds a larger fungal population and higher fungal density, which may also increase the diversification of the harbored viruses. Finally, viral diversity is positively correlated with the genetic diversity of their hosts, and similar host groups tend to have similar viromes. Moreover, a distance-decay pattern was observed in the viral diversity of different *L*. *biglobosa* groups, which may be a consequence of the spread of *L*. *biglobosa* populations carrying these viruses in natural fields. These results indicate that the viruses are in close association with their hosts and that long-term coexistence or coevolution will likely persist between the viruses and *L*. *biglobosa* populations. This finding is consistent with their limited transmission ability and benign infections [62]. Our observations further support our conclusion that the relationship between mycoviruses and fungal hosts is likely to be symbiotic rather than parasitic, and the virome of fungi, as a type of endohhyphal microbiome [63], may resemble the gut microbiome of humans. Despite the extensive diversity of mycoviruses that has been uncovered, our current understanding of mycoviral ecology remains limited [64]. In the present study, LbLV1, a core virome member, plays an essential role during the over-summering of its host fungus by enhancing host thermal tolerance, which may further increase the epidemic effect of *L*. *biglobosa* on winter oilseed rape.

Published simulations of crop disease risk indicate that, over the projected timeframe, the likelihood of increased crop disease risk (60%) surpasses that of decreased (31%) or stable risk (9%), primarily due to global warming [65]. Studies reporting a decline in disease incidence often attribute this trend to supra-optimal temperature conditions caused by climate warming, which exceed the optimal range for disease development [65]. However, these studies rarely consider the potential thermal adaptation of plant pathogens. In mammals, the “super fungus” *Candida auris* has developed in response to global warming events and continues to pose a hazard to public health worldwide [66]. Similarly, our findings demonstrate that fungal vigor and thermal tolerance can be significantly enhanced through the acquisition of a mycovirus—an adaptation mechanism that may be more feasible than mutation and horizontal gene transfer, the traditional pathways. Therefore, our results suggest that climate warming may exacerbate disease occurrence to a greater extent than previously estimated. In addition to *L. biglobosa*, *S*. *sclerotiorum*, and *E*. *necator*, LbLV1 has also been detected in *Monilinia* spp. at a relatively high incidence (unpublished data), indicating the widespread of this virus. Therefore, we believe that this is not an isolated case and that the mycovirome plays an important role in the fungal life cycle. The function of the virome in plant pathogenic fungi is worth exploring because it will further deepen our understanding of epidemics of plant diseases as well as disease management.

## Supplementary Material

Supplemental_Materials_ZKMS_25_1_R2_wrag001-R

## Data Availability

The sequence reads after QC generated from the 17 libraries in this study have been deposited in the NCBI Sequence Read Archive (SRA) database under the BioProject accession numbers PRJNA874489 and PRJNA872307. The genome sequences of all the viruses generated in this study have also been deposited in GenBank and assigned accession numbers OP441667—OP441717, OQ650196, OQ650197, OP392999, and OP393000. All the data are available in the main text or the supplementary materials.
