# Supplementary material for: Core virome shapes adaptation of a phytopathogenic fungus to climate and cropping patterns
Source: ISME J. 2026 Jan 12;20(1):wrag001. doi: 10.1093/ismejo/wrag001 (PMC12904283; doi:10.1093/ismejo/wrag001)
Supplement: Supplemental_Materials_ZKMS_25_1_R2_wrag001-R [file supplemental_materials_zkms_25_1_r2_wrag001-r.pdf]

Supplementary Figures and Tables

Core virome shapes adaptation of a phytopathogenic fungus to climate and cropping patterns

Kang Zhou[1,2], Yue Deng[1,2], Chenghuan Zhu[1,2], Long Yang[1,2], Jing Zhang[1,2], Weidong Chen[3], Nobuhiro Suzuki[4], Guoqing Li[1,2], Mingde Wu[1,2*]

1 State Key Laboratory of Agricultural Microbiology, Huazhong Agricultural University, Wuhan 430070, China.

2 Hubei Key Laboratory of Plant Pathology, Huazhong Agricultural University, Wuhan 430070, China

3 U.S. Department of Agriculture, Agricultural Research Service, Washington State University, Pullman, WA 99164, USA.

4 Institute of Plant Science and Resources, Okayama University, 2-20-1 Chu-ou, Kurashiki, Okayama 710-0046, Japan.

*Corresponding author: Dr. Mingde Wu, E-mail: mingde@mail.hzau.edu.cn.

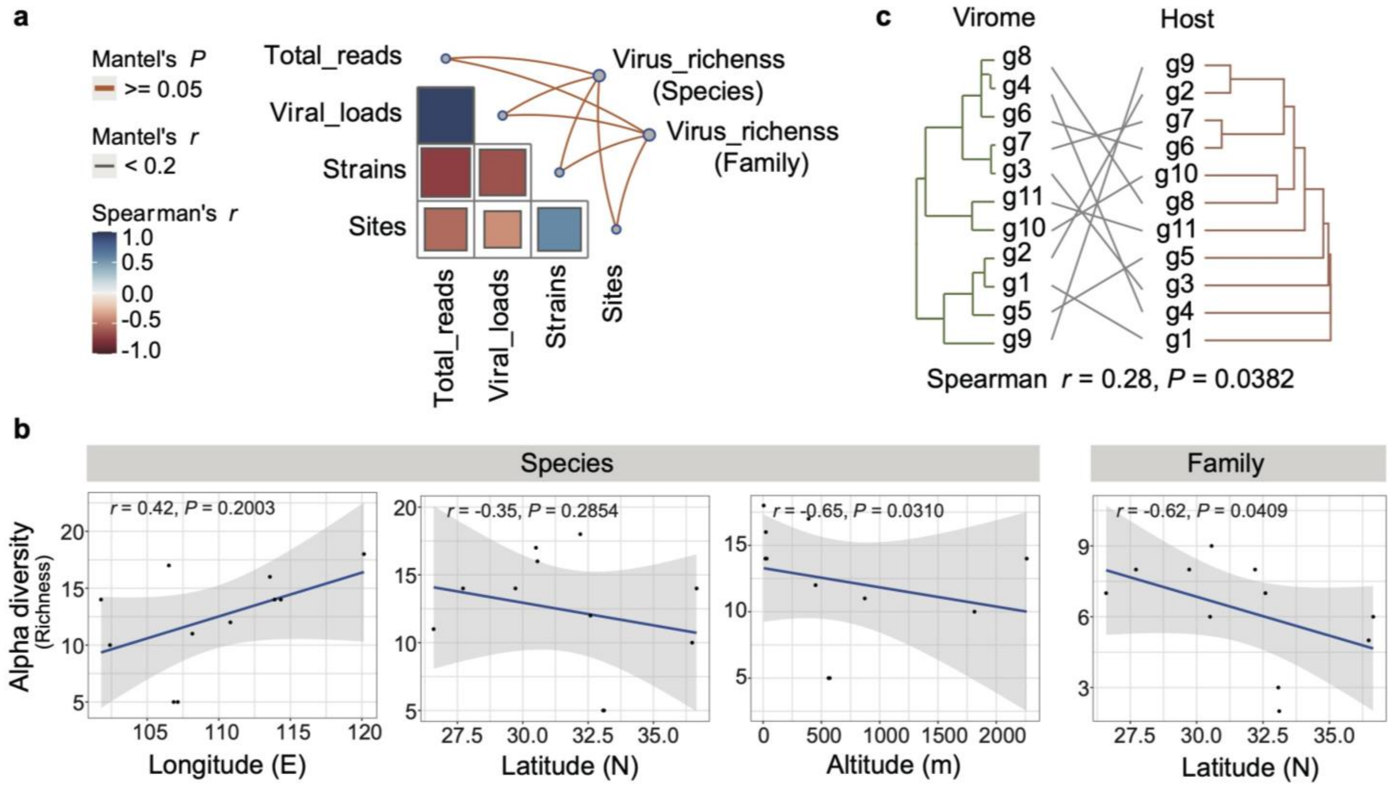

Fig. S1. Association analysis between viral diversity, host genetic diversity, longitude, and latitude.

(a) Association analysis between strain numbers, sampling sites, total reads, and total viral reads, and their associations with the richness indices of viral species and viral family. The correlations between sampling sites, the strain numbers of each group, the total number of viral reads per group, and the total library reads were indicated in a heatmap. The cell colors indicate the “Spearman's r” value. Virus species and family represent the richness indexes of virus species and family in each group, respectively. Each line indicates an association between them, the orange color indicates *P* value is larger than 0.05 based on the association analysis and the thickness of line indicates the “Mantel's r” value is less than 0.2.

(b) Associations between the viral species richness and the longitude as well as the latitude and altitude, and the association between the viral family richness and the latitude. The y axes refer to the viral richness index, while the x axes refer to the longitude (E), latitude (N), and altitude (m), respectively. The fitted linear regression line is shown, with standardized *r* and *P* values.

(c) Phylosymbiosis between the *Leptosphaeria biglobosa* groups and their viromes. The clustering analysis of virome was based on the viral relative abundance matrix with the calculation of bray-curtis distances and unweighted averaging to construct clusters. The phylogeny of *L. biglobosa* populations was based on the genetic distances between populations with the simple sequence repeat analysis, and the phylogenetic tree was constructed based on unweighted averaging.

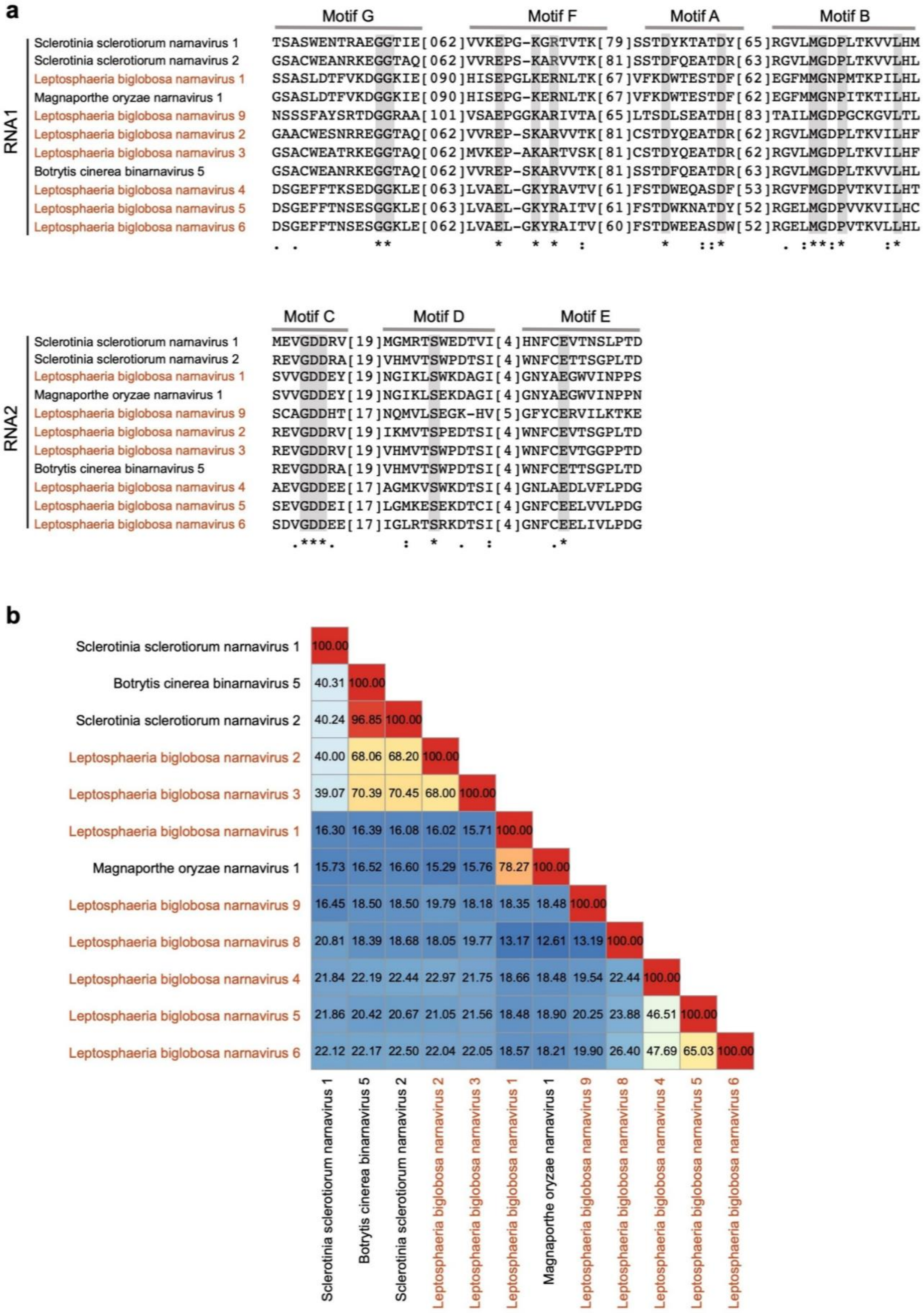

Figure S2. Conserved motifs and similarity matrix of narna-like viruses. (a) Conserved amino acid sequence motifs of the putative RNA-dependent RNA polymerases of Leptosphaeria biglobosa narnavirus 1 (LbNV1), LbNV2, LbNV3, LbNV4, LbNV5, LbNV6, LbNV9, Sclerotinia sclerotiorum narnavirus 1, Sclerotinia sclerotiorum narnavirus 2, Magnaporthe oryzae narnavirus 1 and Botrytis cinerea binarnavirus 5. "*" indicates identical amino acid residues; and "." indicates low chemically similar amino acid residues. (b) The matrix shows the percent identity of the RdRp proteins in the members of *Narnaviridae* family.

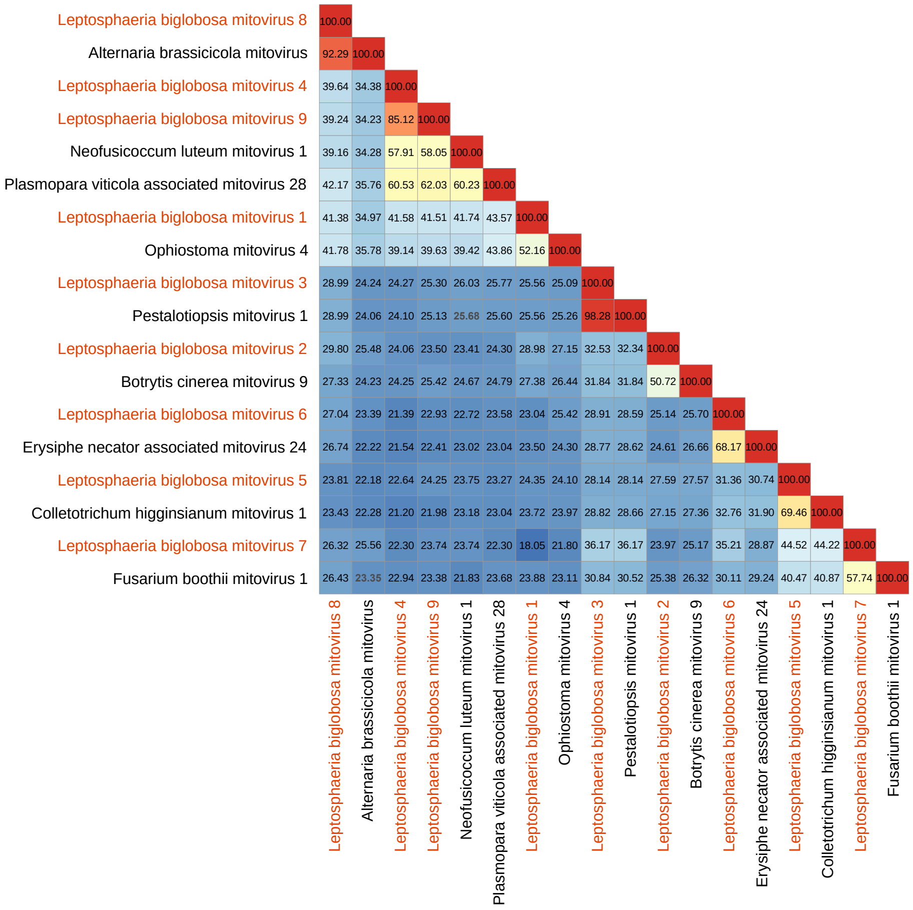

Figure S3. The matrix shows the percent identity of the RdRp proteins in the members of *Mitoviridae* family.

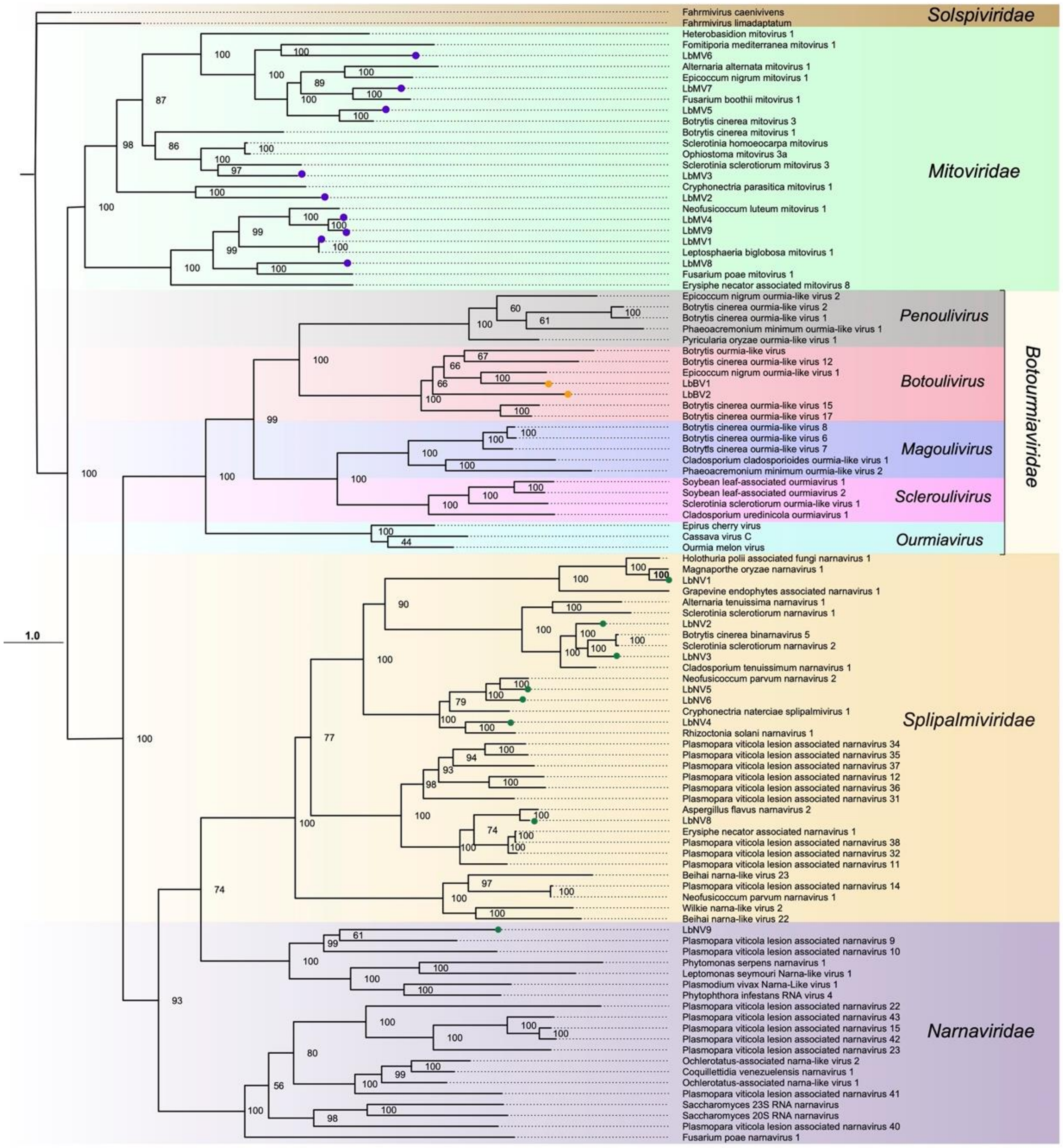

Figure S4. **The phylogenetic tree of mycoviruses within the families *Narnaviridae*, *Botourmiaviridae*, *Mitoviridae* and *Splipalmiviridae*.** All amino acid sequences of RNA-dependent RNA polymerases (RdRps) were aligned with Muscle and then phylogeny was derived using maximum likelihood (ML) in IQ-tree (bootstrap analysis of 1000 replicates, Best-fit model: VT+F+I+R7). The narna-like splipalmiviruses and narnaviruses is indicated in green circle. The botourmiaviruses and mitoviruses are indicated in orange and purple circles, respectively.

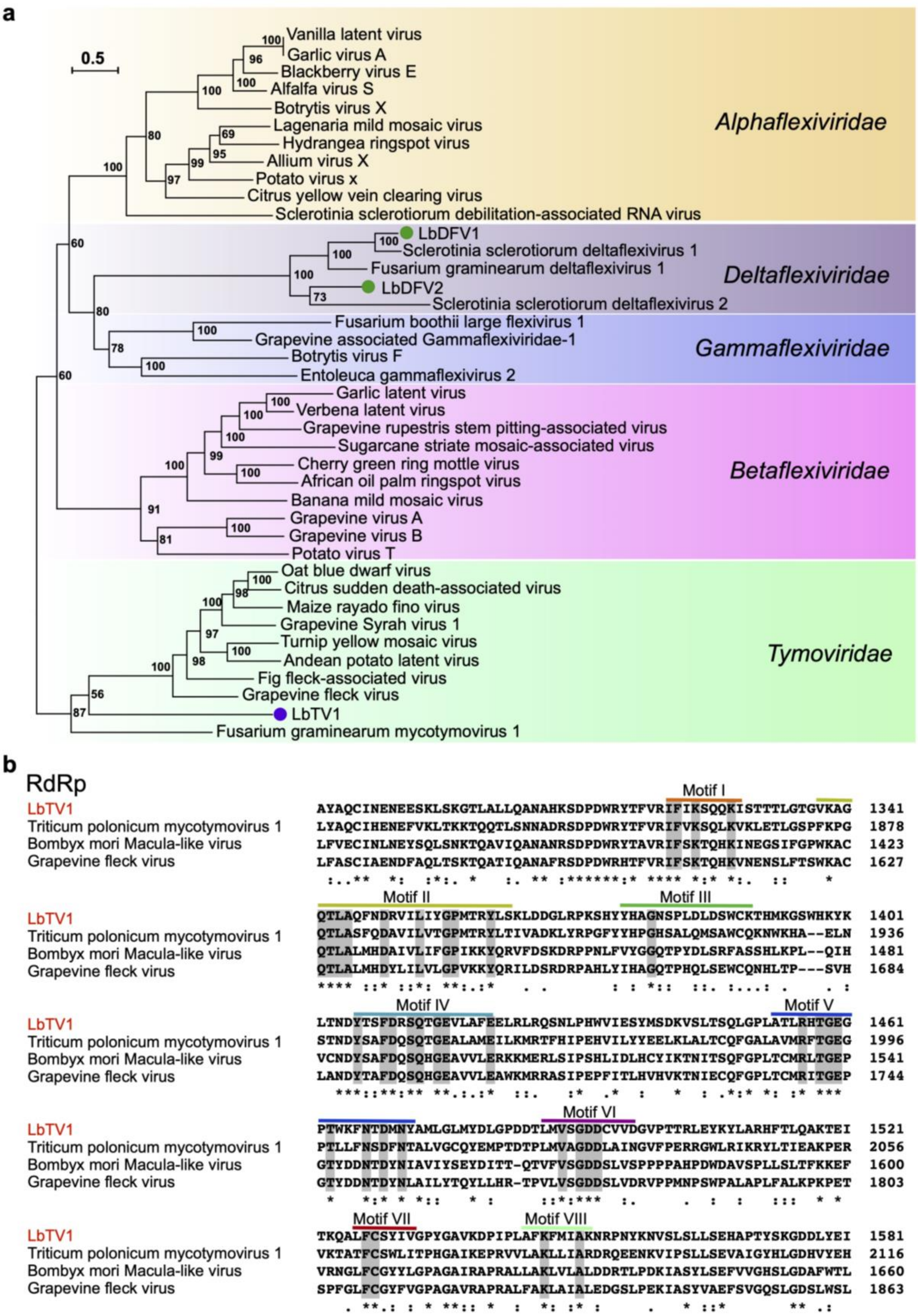

Figure S5. **The phylogenetic analysis of mycoviruses belonged to the order *Tymovirales* and conserved motifs analysis of Leptosphaeria biglobosa tymo-like virus 1 (LbTV1).** (a) The NJ phylogenetic tree was constructed based on multiple alignments of the amino acid sequences of the polyproteins of members of the order *Tymovirales*. The Leptosphaeria biglobosa deltaflexivirus 1 (LbDFV1) and LbDFV2 are indicated in green circles. The LbTV1 is indicated in purple circle. The number next to each branch represents the bootstrap support based on 1000 replicates. (b) Multiple sequence alignments of the conserved RdRp domain of LbTV1 and members of the family *Tymoviridae*. The eight conserved motifs in the RdRp are indicated above the sequence. "*" indicates identical amino acid residues; and "." indicates low chemically similar amino acid residues.

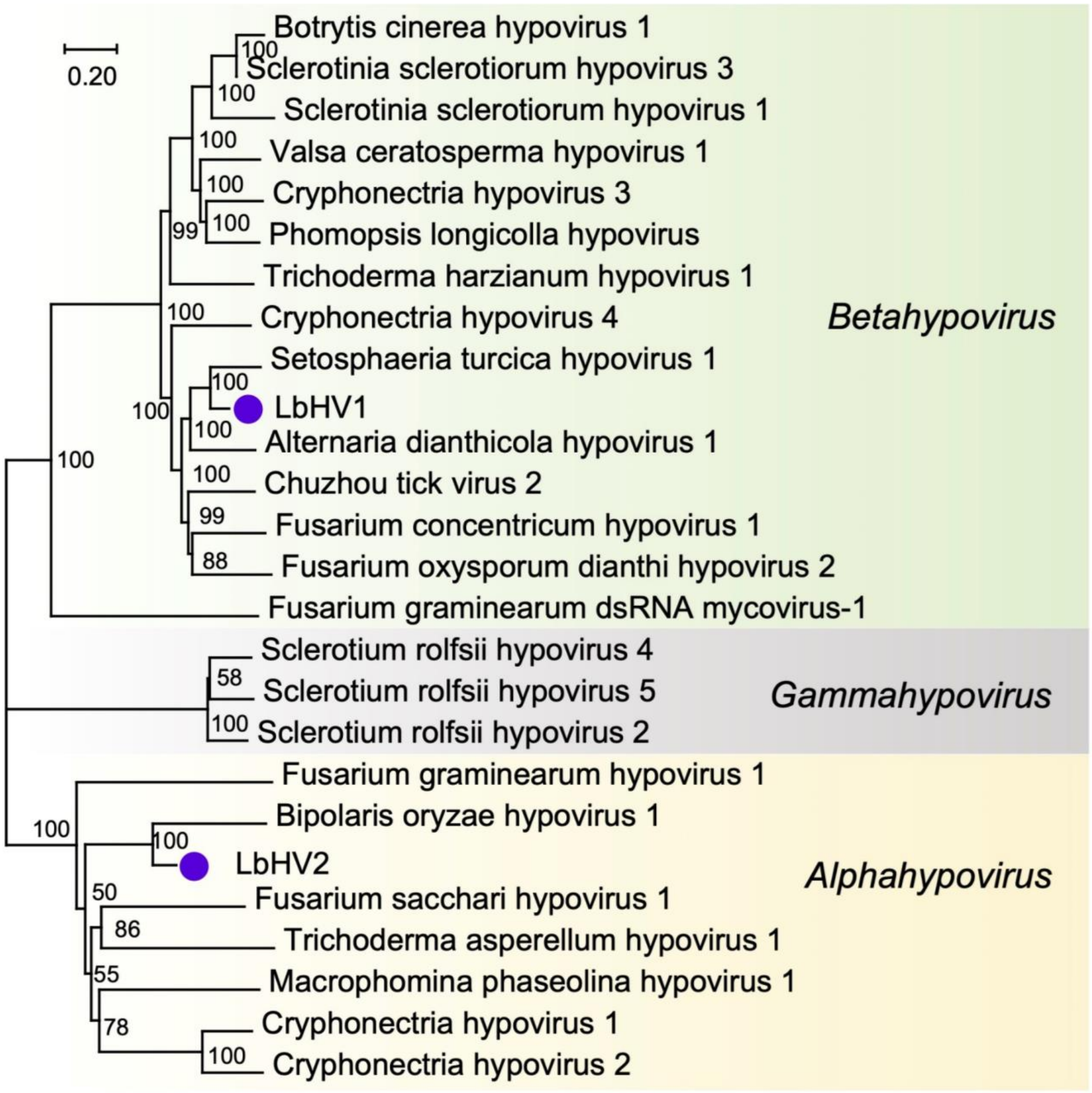

Figure S6. The neighbor-joining phylogenetic tree was constructed based on multiple alignments of the amino acid sequences of the polyproteins of members of the family *Hypoviridae*. The Leptosphaeria biglobosa hypovirus 1 (LbHV1) and LbHV2 are indicated in purple circles. The number next to each branch represents the bootstrap support based on 1000 replicates.

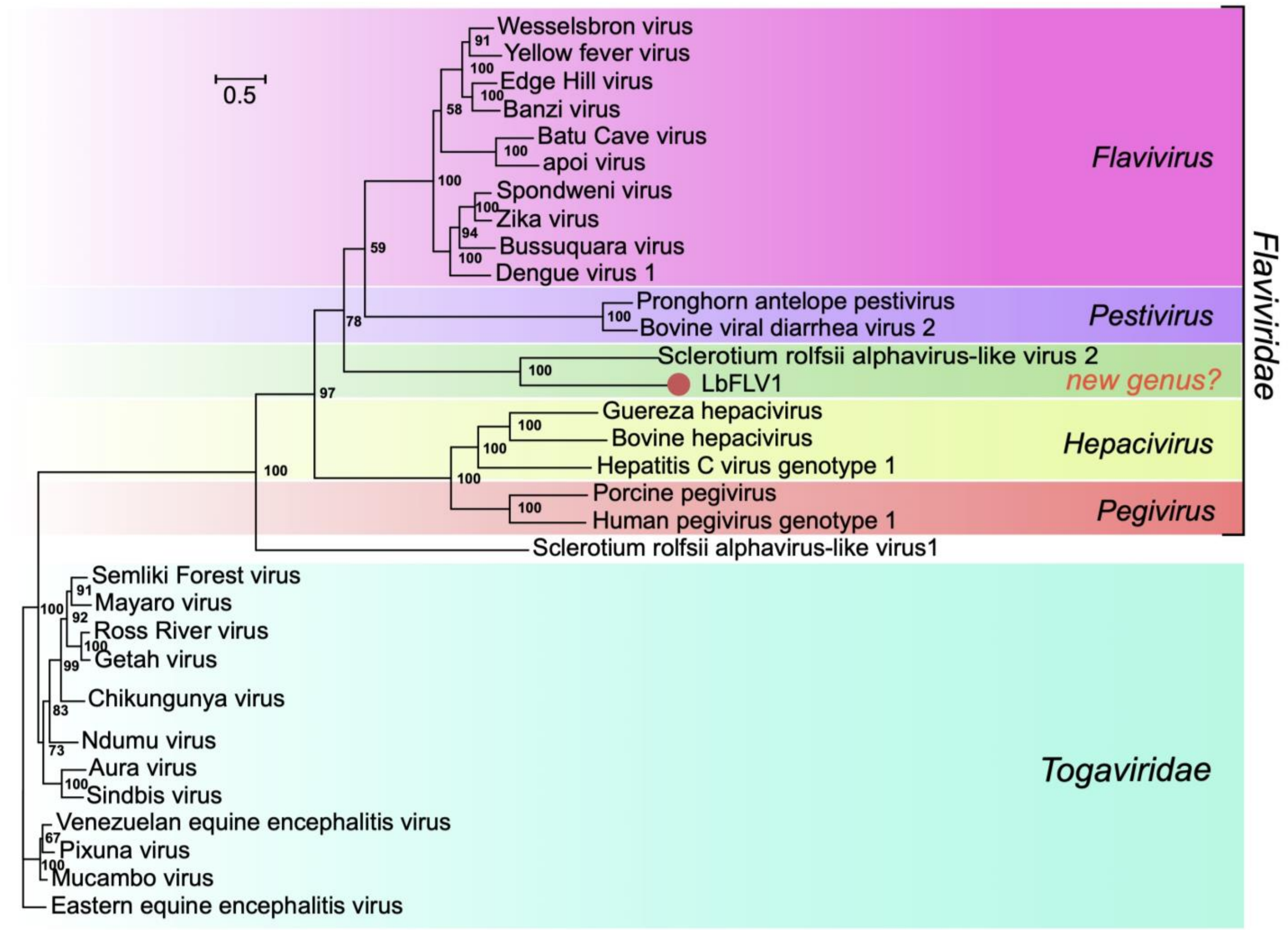

Figure S7. The phylogenetic tree of novel mycovirus within the family *Flaviviridae*. All amino acid sequences of RNA-dependent RNA polymerases (RdRps) were aligned with Muscle and then phylogeny was derived using neighbor-joining (NJ) in MEGA X (bootstrap analysis of 1000 replicates). The Leptosphaeria biglobosa flavi-like virus 1 (LbFLV1) is indicated in red circle.

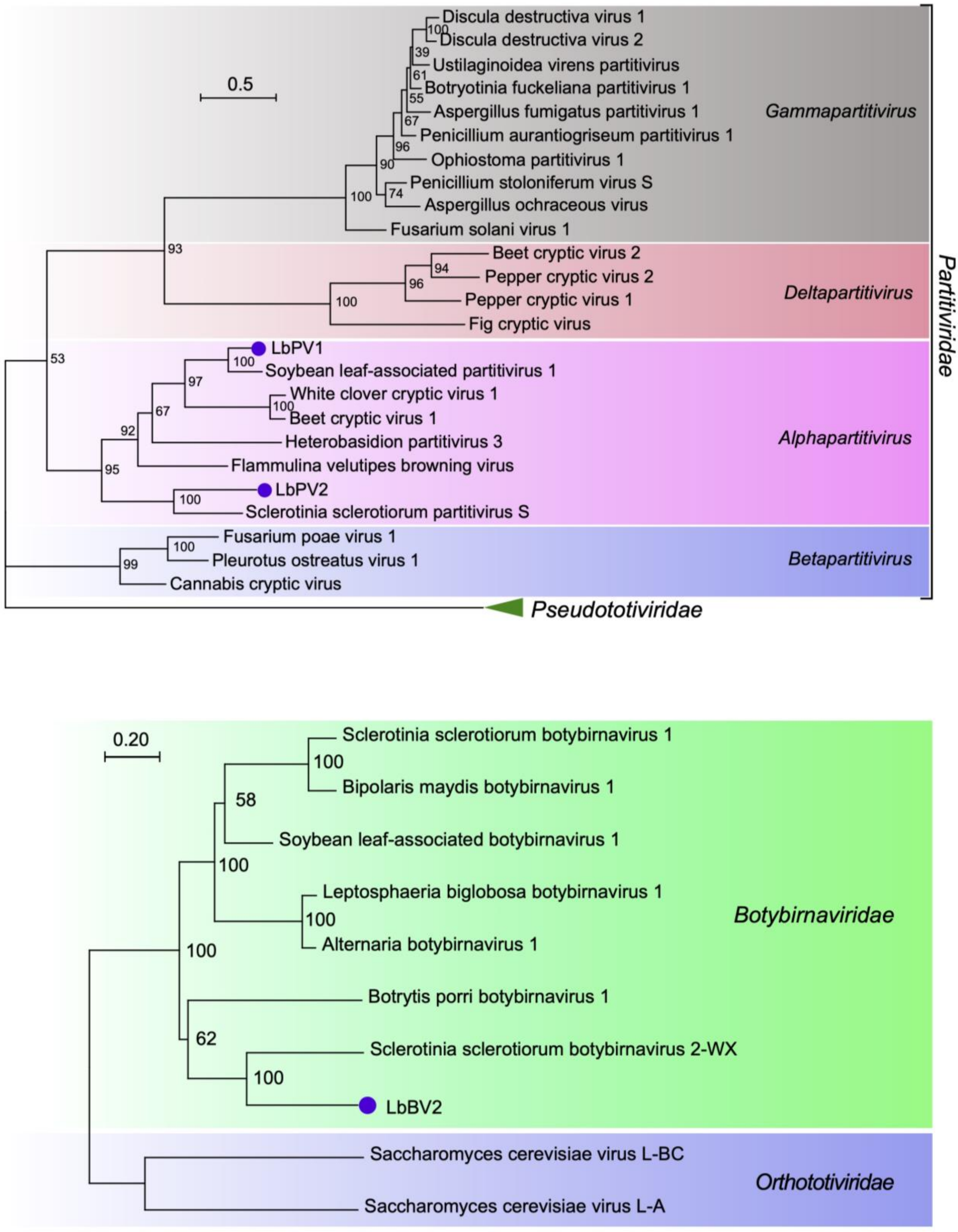

Figure S8. **Phylogenetic trees of mycoviruses within the families *partitiviridae* and *Botybirnaviridae*.** All amino acid sequences of RNA-dependent RNA polymerases (RdRps) were aligned with Muscle and then phylogeny was derived using neighbor-joining (NJ) in MEGA X (bootstrap analysis of 1000 replicates). The Leptosphaeria biglobosa partitivirus 1 (LbPV1), LbPV2 and Leptosphaeria biglobosa botybirnavirus 2 (LbBV2) are indicated in purple circles.

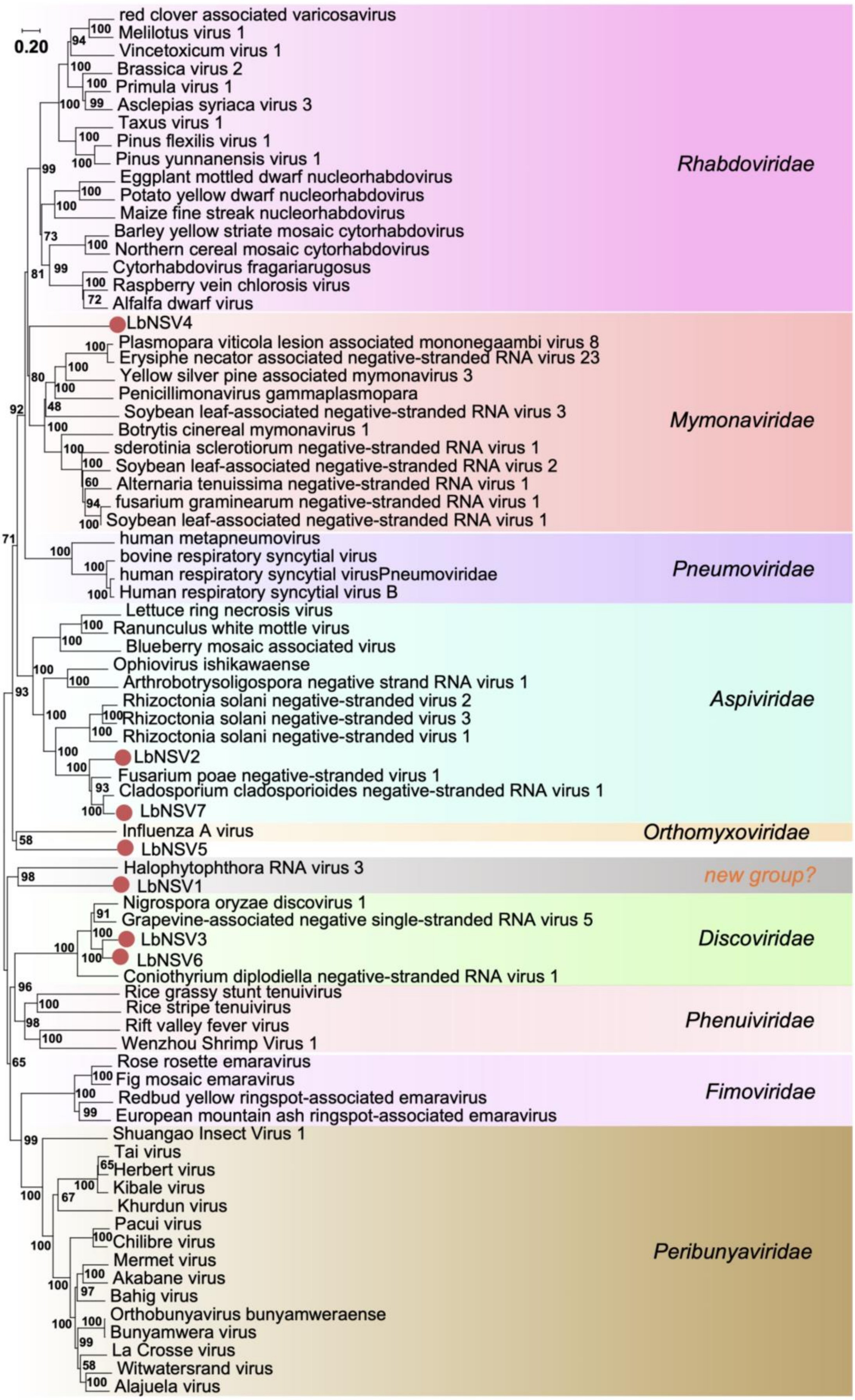

Figure S9. Phylogenetic analysis of the seven novel -ssRNA mycoviruses from the *Leptosphaeria biglobosa* populations. Amino acid sequences of RNA-dependent RNA polymerases (RdRps) were aligned with Muscle and then each phylogenetic tree was estimated using a neighbor-joining method based on conserved viral RdRp (bootstrap analysis of 1000 repeatitions). The scale bar indicates 0.2 amino acid substitutions per site. The viruses newly identified here are marked by with red circles.

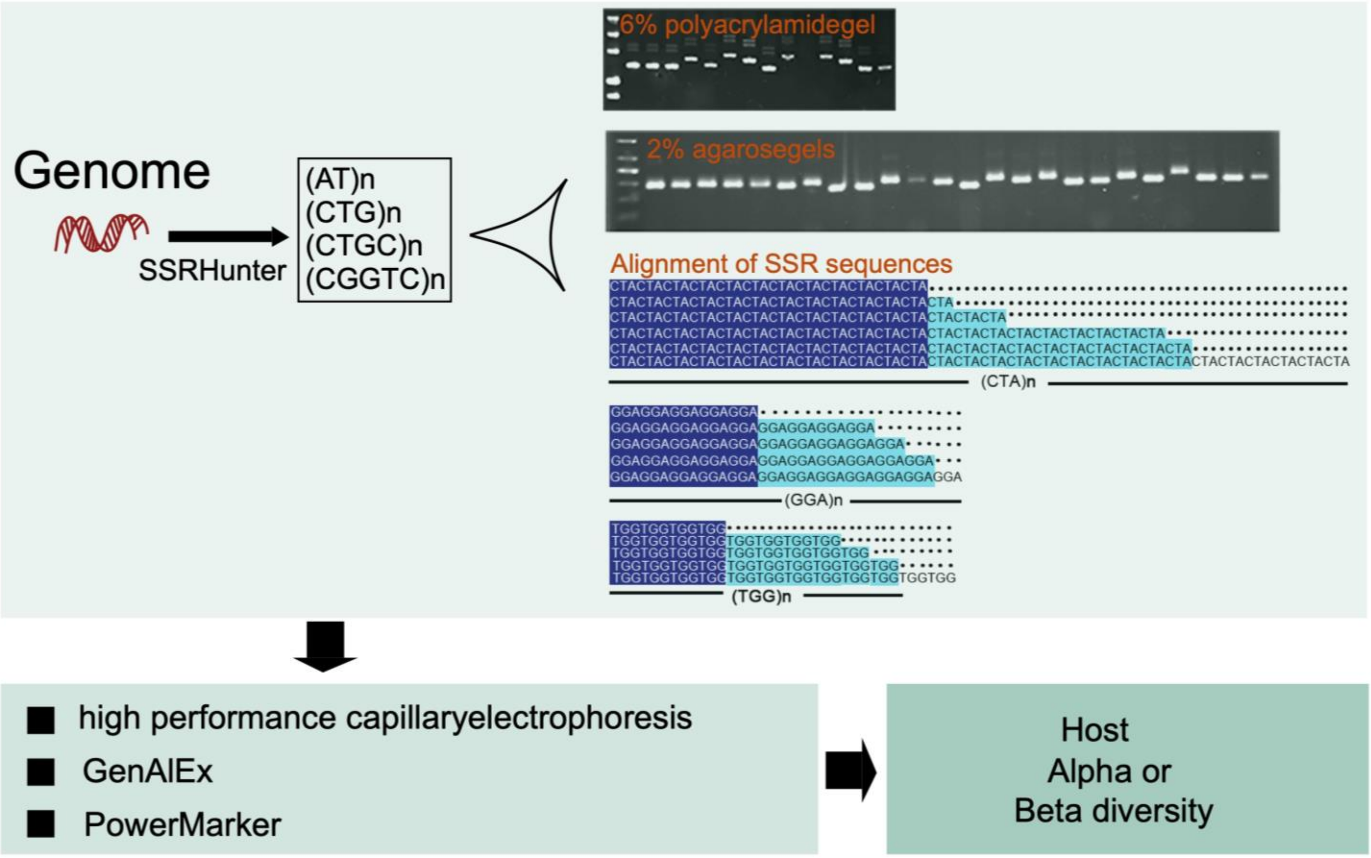

Figure S10. The process of host genetic diversity analysis based on simple sequence repeat markers (SSR).

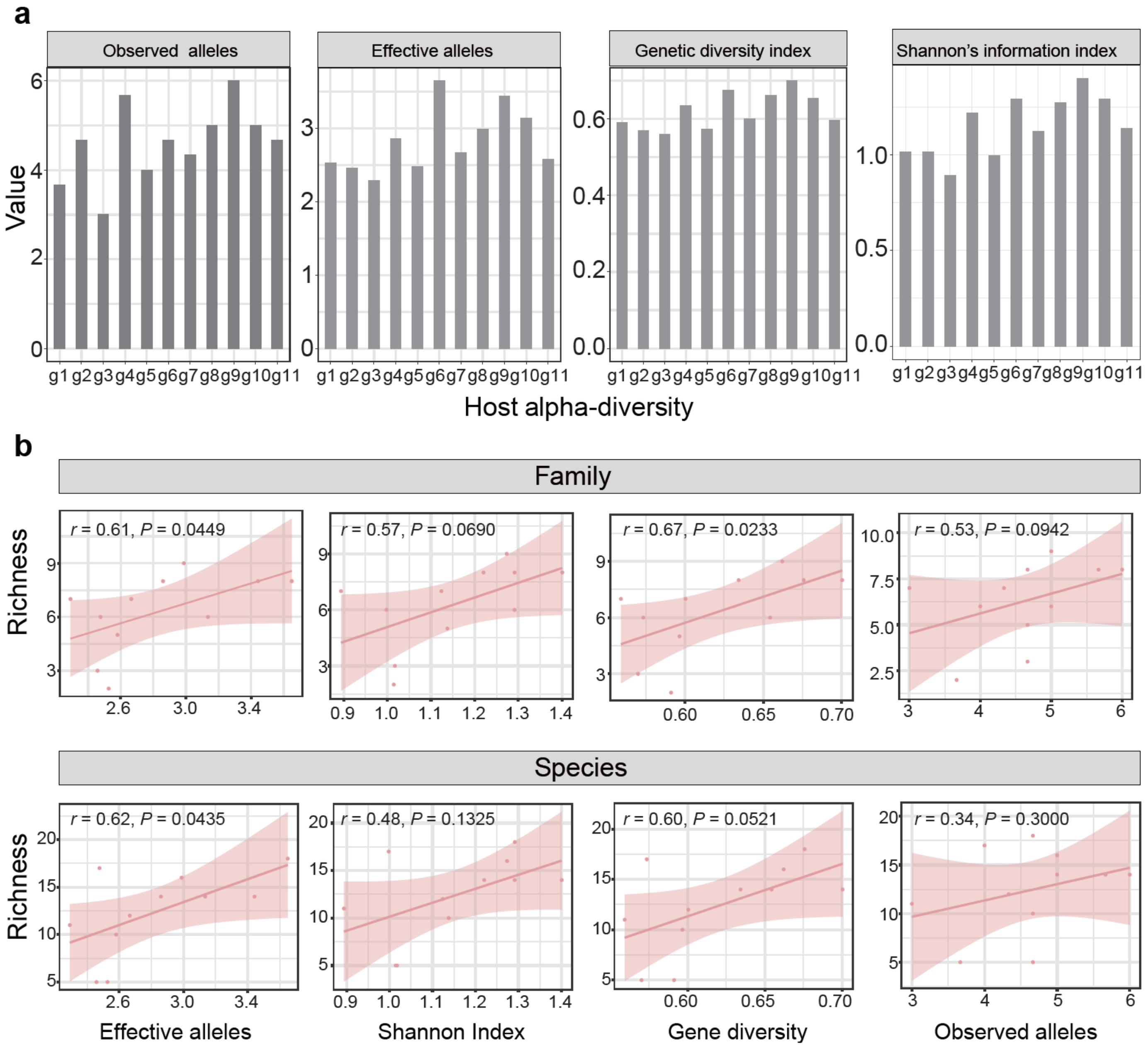

Figure S11. Association analysis between viral diversity and the genetic diversity of *Leptosphaeria biglobosa* strains.

(a) The similarity analysis of genetic diversity between different *L. biglobosa* groups.

(b) The association analysis between viral richness index (species and family level) and host genetic diversity. The fitted linear regression line is shown, with standardized r and *P* values.

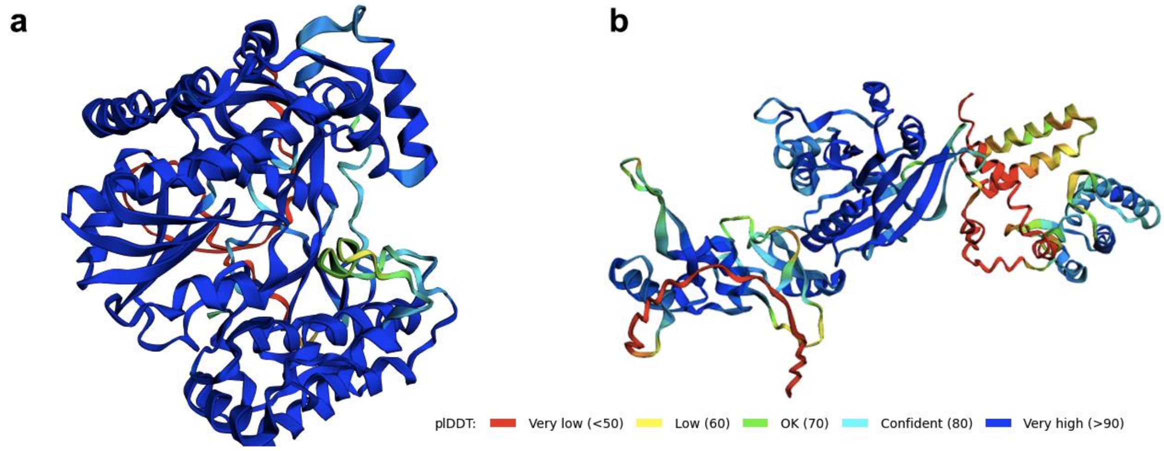

Figure S12. RNA-dependent RNA polymerase (a) and putative capsid protein (HP) (b) structures of LbLV1 predicted by AlphaFold2, with pLDDT confidence scores shown per residue.

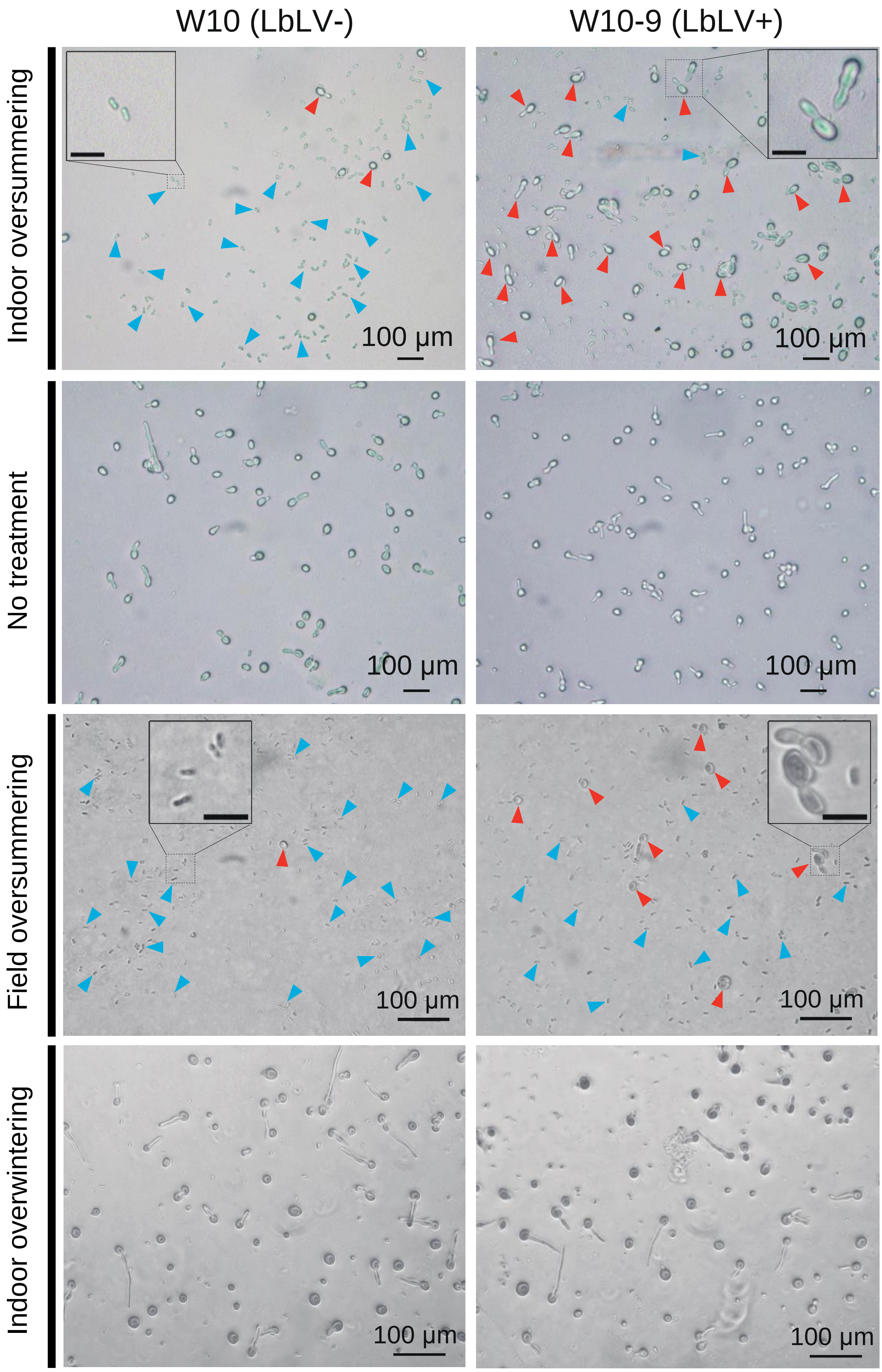

Figure S13. Conidial germination of *Leptosphaeria biglobosa* on potato dextrose agar (PDA, 20°C, 24 h) after indoor and field oversummering treatments, and indoor overwintering treatment. Red and blue arrowheads indicate the living and dead conidia after indoor and field oversummering treatments, respectively. Please note that the living conidia were swollen and some geminated, whereas the dead conidia were small without germination. Most conidia were living under indoor overwintering treatment or no treatment, and no significant differences were observed (Fig. 5). Several conidia from indoor and field oversummering treatments were enlarged in the square frames, the bar represents 50 μm, and please note that most living conidia were geminated with larger sizes compared with the dead conidia.

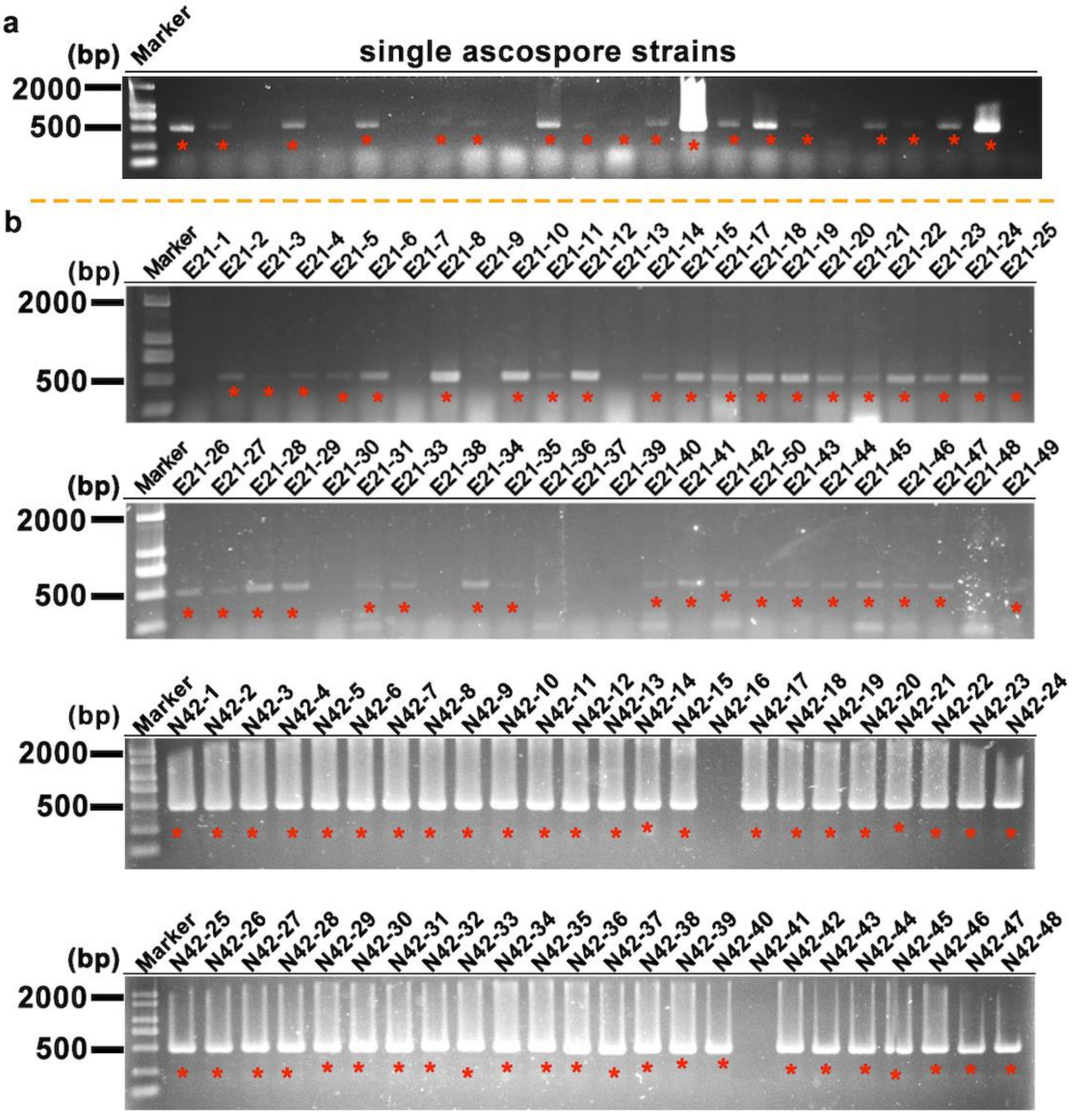

Figure S14. Vertical transmission of Leptosphaeria biglobosa letobirnavirus 1 (LbLV1) through conidia and ascospores.

(a) RT-PCR detection of LbLV1 in single ascospore strains of *L*. *biglobosa* populations in the field.

(b) RT-PCR detection of LbLV1 in single conidial strains of the strains EB9-21 and NN8-42. The target bands are marked with a red asterisk.

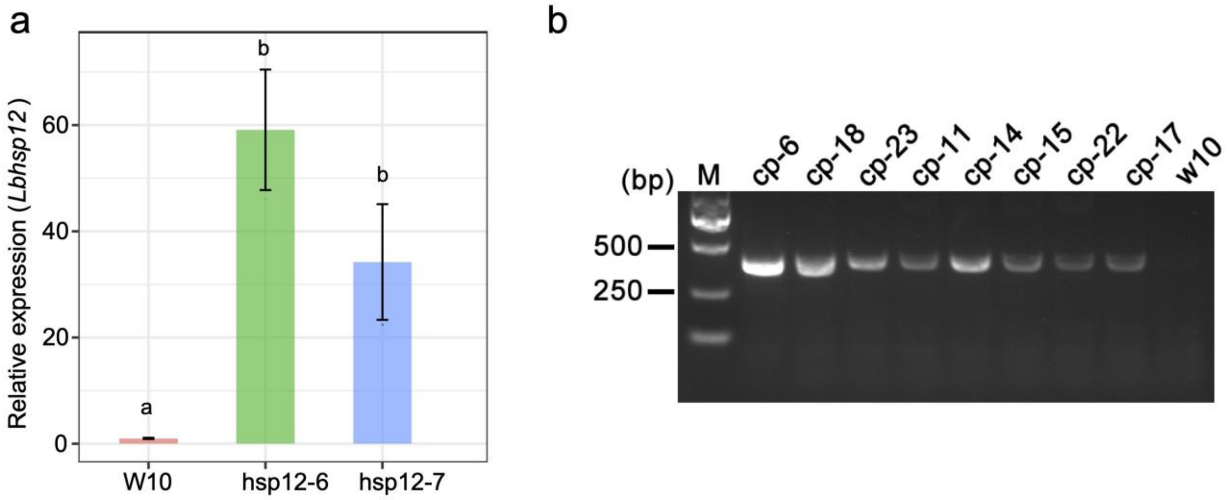

Figure S15. Relative expression levels of *Lbhsp12* in strains W10, hsp12-6, and hsp12-7 (a) and verification of the putative capsid protein (HP) of LbLV1 expression in different strains by RT-PCR (b). Bars labeled with the same letter within a figure are not significantly different ($P > 0.05$) according to the least significant difference test.

Table S1 The information of strains used for virome analysis

Group name	The number of strains	Geographic locations	Types of oilseed rape production area
g1	99	Hantai District and Mian County, Hanzhong City, Shaanxi Province, China	winter
g2	102	Nanzheng District and Chenggu County, Hanzhong City, Shaanxi Province, China	winter
g3	114	Sinan County, Maitan County, Kaiyang County and Quanzhou, Guizhou Province, China	winter
g4	97	Nanchang, Jiangxi province and Hengyang city, Hunan province, China	winter
g5	122	Chengdu, Guang'an, Nanchong, Sichuan Province and Chongqing City, China	winter
g6	111	Nantong and Yangzhou, Jiangsu Province, China	winter
g7	113	Shiyan City, Hubei Province, China	winter
g8	174	Wuhan, Tianmen, Qianjiang and Xiantao, Hubei Province, China	winter
g9	70	Chibi, Hubei Province, China	winter
g10	57	Xining City, Qinghai Province, China	spring
g11	44	Haidong City, Qinghai Province, China	spring

Table S2 Reference sequences used for the construction of the phylogenetic tree

**Virus name or short name**	**Accession number**	**Family**
Soybean leaf-associated ourmiavirus 1	NC_043521	*Botourmiaviridae*
Soybean leaf-associated ourmiavirus 2	NC_043522	*Botourmiaviridae*
Sclerotinia sclerotiorum ourmia-like virus 1	KP900928	*Botourmiaviridae*
Plasmopara viticola associated ourmia-like virus 44	MN532631	*Botourmiaviridae*
Colletotrichum gloeosporioides ourmia-like virus 1	MK542706	*Botourmiaviridae*
Magnaporthe oryzae ourmia-like virus	NC_043683	*Botourmiaviridae*
Penicillium citrinum ourmia-like virus 1	MG887747	*Botourmiaviridae*
Rhizoctonia solani ourmia-like virus 1	KP900922.1	*Botourmiaviridae*
Pyricularia oryzae ourmia-like virus 1	LC413501	*Botourmiaviridae*
Entoleuca ourmia-like virus 1	MF375889	*Botourmiaviridae*
Phaeoacremonium minimum ourmia-like virus 3	MK584848	*Botourmiaviridae*
Plasmopara viticola associated ourmia-like virus 81	MN532668	*Botourmiaviridae*
Phoma matteucciicola ourmia-like virus 1	QIP68359	*Botourmiaviridae*
Rhizoctonia solani ourmia-like virus 1	ALD89131	*Botourmiaviridae*
Botrytis ourmia-like virus	MT119674	*Botourmiaviridae*
Sclerotinia sclerotiorum ourmia-like virus 2	KP900929	*Botourmiaviridae*
Cassava virus C	NC_013113	*Botourmiaviridae*
Ourmia melon virus	NC_011070	*Botourmiaviridae*
Fusarium poae narnavirus 1	NC_030865	*Narnaviridae*
Saccharomyces 23S RNA narnavirus	NC_004050	*Narnaviridae*
Saccharomyces 20S RNA narnavirus	NC_004051	*Narnaviridae*
Blechmonas luni narnavirus 1	NC_040829	*Narnaviridae*
Blechomonas wendygibsoni narnavirus 1	NC_040641	*Narnaviridae*
Leptosphaeria biglobosa mitovirus 1	NC_040819	*Mitoviridae*
Neofusicoccum luteum mitovirus 1	NC_035114	*Mitoviridae*
Fusarium poae mitovirus 1	NC_030861	*Mitoviridae*
Cryphonectria parasitica mitovirus 1	NC_004046	*Mitoviridae*
Cryphonectria parasitica mitovirus 1-NB631	NP_660174	*Mitoviridae*
Ophiostoma mitovirus 1a	CAJ32466	*Mitoviridae*
Cryphonectria cubensis mitovirus 2a	AAR01973	*Mitoviridae*
Gigaspora margarita mitovirus 1	NC_040702	*Mitoviridae*
Clitocybe odora virus	NC_017003	*Mitoviridae*
Heterobasidion mitovirus 1	KJ873059	*Mitoviridae*
Sclerotinia homoeocarpa mitovirus	AY172454	*Mitoviridae*
Ophiostoma mitovirus 3a	NC_004049	*Mitoviridae*
Sclerotinia sclerotiorum mitovirus 3	NC_028475	*Mitoviridae*
Botrytis cinerea mitovirus 1	NC_011372	*Mitoviridae*
Tuber aestivum mitovirus	NC_015629	*Mitoviridae*
Fomitiporia mediterranea mitovirus 1	MK584830	*Mitoviridae*
Alternaria alternata mitovirus 1	MK584829	*Mitoviridae*
Epicoccum nigrum mitovirus 1	MK584828	*Mitoviridae*

Fusarium boothii mitovirus 1	LC425114	*Mitoviridae*
Botrytis cinerea mitovirus 3	NC_028472	*Mitoviridae*
Alfalfa virus S| AVS	NC_034622	*Alphaflexiviridae*
Blackberry virus E| BVE	NC_015706	*Alphaflexiviridae*
Garlic virus A| GVA	NC_003375	*Alphaflexiviridae*
Vanilla latent virus| VLV	NC_035204	*Alphaflexiviridae*
Botrytis virus X| BVX	NC_005132	*Alphaflexiviridae*
Citrus yellow vein clearing virus| CYVCV	NC_026592	*Alphaflexiviridae*
Potato virus x| PVX	NC_011620	*Alphaflexiviridae*
Hydrangea ringspot virus| HRV	NC_006943	*Alphaflexiviridae*
Allium virus X| AVX	NC_012211	*Alphaflexiviridae*
Lagenaria mild mosaic virus| LMMV	NC_043079	*Alphaflexiviridae*
Sclerotinia sclerotiorum debilitation-associated RNA virus| SsDARV	NC_007415	*Alphaflexiviridae*
Grapevine virus A| GVA	NC_003604	*Betaflexiviridae*
Grapevine virus B| GVB	NC_003602	*Betaflexiviridae*
Potato virus T| PVT	NC_011062	*Betaflexiviridae*
Camellia ringspot associated virus 3| CRAV3	MK050796	*Betaflexiviridae*
Banana mild mosaic virus| BMMV	NC_002729	*Betaflexiviridae*
Sugarcane striate mosaic-associated virus| SSMAV	NC_003870	*Betaflexiviridae*
African oil palm ringspot virus| AOPRV	NC_012519	*Betaflexiviridae*
Cherry green ring mottle virus| CGMV	NC_001946	*Betaflexiviridae*
Grapevine rupestris stem pitting-associated virus| GRSTAV	NC_001948	*Betaflexiviridae*
Verbena latent virus| VLV	NC_043085	*Betaflexiviridae*
Garlic latent virus| GLV	NC_003557	*Betaflexiviridae*
Fusarium boothii large flexivirus 1| FbFLV1	LC425116	*Gammaflexiviridae*
Grapevine associated Gammaflexiviridae-1| GAGLV1	HM852917	*Gammaflexiviridae*
Botrytis virus F| BVF	NC_002604	*Gammaflexiviridae*
Entoleuca gammaflexivirus 2| EGLV2	MF375884	*Gammaflexiviridae*
Fusarium graminearum mycotymovirus 1| FgMV1	NC_040784	*Tymoviridae*
Grapevine fleck virus| GLV	NC_003347	*Tymoviridae*
Fig fleck-associated virus| FFAV	NC_015229	*Tymoviridae*
Turnip yellow mosaic virus| TYMV	NC_004063	*Tymoviridae*
Andean potato latent virus| APLV	NC_020470	*Tymoviridae*
Grapevine Syrah virus 1| GSV1	NC_012484	*Tymoviridae*
Maize rayado fino virus| MRFV	NC_002786	*Tymoviridae*
Citrus sudden death-associated virus| CSDAV	NC_006950	*Tymoviridae*
Oat blue dwarf virus| OBDV	NC_001793	*Tymoviridae*
Rosellinia necatrix fusarivirus 1| RnFV1	NC_024485.1	*Fusariviridae*
Sindbis virus| SV	NC_001547.1	*Togaviridae*
Aura virus| AV	NC_003900.1	*Togaviridae*
Eastern equine encephalitis virus| EEEV	AY722102.1	*Togaviridae*
Mucambo virus| MV	MF993533.1	*Togaviridae*

Pixuna virus| PV	NC_038673.1	*Togaviridae*
Venezuelan equine encephalitis virus| VEEV	NC_075022.1	*Togaviridae*
Sclerotinia sclerotiorum virga-like virus 1| SsVLV1	MT646383.1	*Mycovirgaviridae*
Grapevine-associated RNA virus 4| GARV4	MW648525	*Mycovirgaviridae*
Sclerotinia sclerotiorum endornavirus 1| SsEV1	NC_021706.1	*Endornaviridae*
Sclerotinia sclerotiorum endornavirus 2| SsEV2	KU299046.1	*Endornaviridae*
Botrytis cinerea betaendornavirus 1| BcEV1	KU923747.1	*Endornaviridae*
Sclerotinia sclerotiorum endornavirus 8| SsEV8	MT646356	*Endornaviridae*
Sclerotinia minor endornavirus 1| SmEV1	NC_040631.1	*Endornaviridae*
Sclerotinia sclerotiorum endornavirus 9| SsEV9	MT646421	*Endornaviridae*
Rosellinia necatrix endornavirus 1| RnEV1	NC_030938.1	*Endornaviridae*
Alternaria brassicicola endornavirus| AbEV	NC_026136.1	*Endornaviridae*
Riboviria sp.	MN032949	*Virgaviridae*
Ligustrum mosaic virus| LMV	MW752157.1	*Virgaviridae*
Poa semilatent virus| PSV	MK377386.1	*Virgaviridae*
Lychnis ringspot virus| LRV	MG913809.1	*Virgaviridae*
Barley stripe mosaic virus| BSMV	MK377386.1	*Virgaviridae*
Drakaea virus A| DVA	NC_043398.1	*Virgaviridae*
Gentian ovary ringspot virus| GORV	NC_024501.1	*Virgaviridae*
Erysiphe necator associated gora-like virus 1| EnAGV	MN630187.1	*Virgaviridae*
Peanut clump virus| PCV	NC_003672.1	*Virgaviridae*
Citrus leprosis virus C| CLVC	MW574405	*Kitaviridae*
Raspberry bushy dwarf virus| RBDV	NC_003739.1	*Mayoviridae*
Neofusicoccum parvum RNA virus 1| NpRV1	MK584849	*Mycovirgaviridae*
Penicillium glabrum RNA virus 1| PgRV1	MK584850.1	*Mycovirgaviridae*
Tomato mosaic virus | TMV	AJ417701.1	*Virgaviridae*
Bell pepper mottle virus| BPMV	MK770674.1	*Virgaviridae*
Tobacco mild green mosaic virus| TMGMV	PP623115.1	*Virgaviridae*
Cucumber fruit mottle mosaic virus| CFMMV	MT989352.1	*Virgaviridae*
Erysiphe necator associated virga-like virus 5| EnAVLV5	MN627449	*Mycovirgaviridae*
Erysiphe necator associated virga-like virus 6| EnAVLV6	MN627455.1	*Mycovirgaviridae*
Plasmopara viticola lesion associated vivivirus 4| PvLAV4	MT338022.1	*Mycovirgaviridae*
Luckshill virus| LV	MF893250	*Virgaviridae*
Erysiphe necator associated virga-like virus 13| EnAVLV13	MN627446	*Mycovirgaviridae*
Erysiphe necator associated virga-like virus 8| EnAVLV8	MN627467	*Mycovirgaviridae*
Sisal-associated virgavirus A|SAVA	MZ329757	*Virgaviridae*
Rhizoctonia solani flexivirus 1|RsFLV1	NC_030655	*unclassified*
Sclerotinia sclerotiorum deltaflexivirus 2|SsDFV2	NC_040649	*Deltaflexiviridae*
Fusarium graminearum deltaflexivirus 1|FgDFV1	NC_030654	*Deltaflexiviridae*

Sclerotinia sclerotiorum deltaflexivirus 1| SsDFV1	NC_038977	*Deltaflexiviridae*
Fusarium graminearum negative-stranded RNA virus 1	MF276904	*Mymonaviridae*
Soybean leaf-associated negative-stranded RNA virus 1	KT598225	*Mymonaviridae*
Alternaria tenuissima negative-stranded RNA virus 1	MK584852	*Mymonaviridae*
Soybean leaf-associated negative-stranded RNA virus 2	KT598227	*Mymonaviridae*
Rose rosette emaravirus	YP_004327589	*Mymonaviridae*
Fig mosaic emaravirus	YP_009237269	*Mymonaviridae*
sderotinia sclerotiorum negative-stranded RNA virus 1	NC_025383	*Mymonaviridae*
sderotinia sclerotiorum negative-stranded RNA virus 3	NC_026732	*Mymonaviridae*
Botrytis cinereal mymonavirus 1	MH648611	*Mymonaviridae*
Kiln barn virus	MF893248	*Mymonaviridae*
Penicillium adametzioides negative-stranded RNA virus 1	MK584858	*Mymonaviridae*
Soybean leaf-associated negative-stranded RNA virus 4	KT598229	*Mymonaviridae*
Soybean leaf-associated negative-stranded RNA virus 3	KT598228	*Mymonaviridae*
sderotinia sclerotiorum negative-stranded RNA virus 2	KP900931	*Mymonaviridae*
sderotinia sclerotiorum negative-stranded RNA virus 4	NC_043483	*Mymonaviridae*
Barley yellow striate mosaic cytorhabdovirus	NC_028244	*Rhabdoviridae*
Northern cereal mosaic cytorhabdovirus	NC_002251	*Rhabdoviridae*
Maize fine streak nucleorhabdovirus	NC_005974	*Rhabdoviridae*
Eggplant mottled dwarf nucleorhabdovirus	NC_025389	*Rhabdoviridae*
Potato yellow dwarf nucleorhabdovirus	NC_016136	*Rhabdoviridae*
Mumps rubulavirus	NC_002200	*Paramyxoviridae*
Nipah henipavirus	NC_002728	*Paramyxoviridae*
Canine morbillivirus	NC_001921	*Paramyxoviridae*
Measles morbillivirus	NC_001498	*Paramyxoviridae*
Lettuce ring necrosis virus	NC_006054	*Aspiviridae*
Ranunculus white mottle virus	NC_043389	*Aspiviridae*
Blueberry mosaic associated virus	NC_036635	*Aspiviridae*
Cladosporium cladosporioides negative-stranded RNA virus 1	MK584856	unclassified
Fusarium poae negative-stranded virus 1	NC_030871	unclassified
Rhizoctonia solani negative-stranded virus 1	KP900919	unclassified
Rhizoctonia solani negative-stranded virus 2	KP900920	unclassified
Rhizoctonia solani negative-stranded virus 3	KP900903	unclassified
Botrytis cinerea negative-stranded RNA virus 1	NC_028466	unclassified
Macrophomina phaseolina negative-stranded RNA virus	KP900899	unclassified
Rice grassy stunt tenuivirus	NC_002328	*Phenuiviridae*
Rice stripe tenuivirus	NC_003776	*Phenuiviridae*
Rift valley fever virus	NC_014395	*Phenuiviridae*
Wenzhou Shrimp Virus 1	NC_031292	*Phenuiviridae*
Alternaria tenuissima negative-stranded RNA virus 2 AtNsRV2	MK584855	unclassified

Sclerotinia sclerotiorum negative-stranded RNA virus 5	MF444283	unclassified
Coniothyrium diplodiella negative-stranded RNA virus 1	MN532680	unclassified
Rhizoctonia solani negative-stranded virus 4	KP900923	unclassified
Cladosporium cladosporioides negative-stranded RNA virus 2 CcNsRV2	MK584857	unclassified
Hantaan virus	NC_005222	*Hantaviridae*
Groundnut ringspot virus	NC_043503	*Tospoviridae*
Tomato chlorotic spot virus	NC_035484	*Tospoviridae*
Tomato spotted wilt tospovirus	NC_002052	*Tospoviridae*
Groundnut bud necrosis virus	MK875278	*Tospoviridae*
La Crosse virus	NC_004110	*Peribunyaviridae*
Bunyamwera virus	NC_001927	*Peribunyaviridae*
Akabane virus	NC_009895	*Peribunyaviridae*
Rose rosette emaravirus	NC_034981	*Fimoviridae*
Fig mosaic emaravirus	NC_029568	*Fimoviridae*
Redbud yellow ringspot-associated emaravirus	NC_038856	*Fimoviridae*
European mountain ash ringspot-associated emaravirus	NC_013105	*Fimoviridae*
Penicillium aurantiogriseum partitivirus 1	NC_028499	*Gammapartitivirus*
Botryotinia fuckeliana partitivirus 1	NC_010350	*Gammapartitivirus*
Discula destructiva virus 2	NC_003711	*Gammapartitivirus*
Discula destructiva virus 1	NC_002800	*Gammapartitivirus*
Ustilaginoidea virens partitivirus	KJ868799	*Gammapartitivirus*
Aspergillus fumigatus partitivirus 1	LR746165	*Gammapartitivirus*
Ophiostoma partitivirus 1	NC_038918	*Gammapartitivirus*
Penicillium stoloniferum virus S	NC_005977	*Gammapartitivirus*
Aspergillus ochraceous virus	NC_043397	*Gammapartitivirus*
Fusarium solani virus 1	NC_003886	*Gammapartitivirus*
Diatom colony associated dsRNA virus 14	AP014907	*unclassified*
Fig cryptic virus	YP_004429258	*Deltapartitivirus*
Pepper cryptic virus 1	NC_037096	*Deltapartitivirus*
Pepper cryptic virus 2	NC_034159	*Deltapartitivirus*
Beet cryptic virus 2	YP_009508068	*Deltapartitivirus*
Beet cryptic virus 1	YP_002308574	*Deltapartitivirus*
Beet cryptic virus 3	YP_009665971	*Deltapartitivirus*
Pleurotus ostreatus virus 1	NC_006961	*Betapartitivirus*
Fusarium poae virus 1	NC_003884	*Betapartitivirus*
Cannabis cryptic virus	NC_031134	*Betapartitivirus*
Sclerotinia sclerotiorum partitivirus S	NC_013015	*Alphapartitivirus*
Heterobasidion partitivirus 3	NC_038836	*Alphapartitivirus*
Flammulina velutipes browning virus	NC_038826	*Alphapartitivirus*
Soybean leaf-associated partitivirus 1	KT598242	*Alphapartitivirus*
Epirus cherry virus	YP_002019754	*Alphapartitivirus*
White clover cryptic virus 1	NC_006276	*Alphapartitivirus*

Sclerotinia nivalis victorivirus 1	NC_030392	*Totiviridae*

**Gene sequences used for phylogenetic analysis of hsp12**	
**Name**	**Accession number**
Aspergillus affinis hsp12	XP_052953160.1
Saccharomyces cerevisiae hsp12	NP_116640.1
Aspergillus fumigatus hsp12	XP_751123.1
Penicillium roqueforti hsp12	XP_038926393.1
Funaria hygrometrica hsp12	CAC81965.1
Saccharomyces paradoxus hsp12	XP_033766052.1
Sclerotinia sclerotiorum 1980 UF-70 hsp12	XP_001593580.1
Monilinia fructicola hsp12	KAA8569608.1
Botrytis cinerea hsp12	XP_024550959.1
Armillaria gallica hsp12	PBK96964.1
Mycena polygramma hsp12	KAJ7685375.1
Lentinula lateritia hsp12	KAJ3807387.1
Leptosphaeria maculans hsp12	XP_003836375.1

Table S3 Primers and the adaptor used in the present study

**Primers used for cDNA cloning of LbLV1**		
Primer Name	Sequence (5'→ 3')	Position[1]
R1-5out-f[2]	GCAAGGACTGGAGCATAGGAAGGAC	208-232
R1-5out-r	TTTCTTCCCCTCGTCGCTACA	234-254
R1-5in-f	TTACCTACTTAAAGGGAAATAGACGTG	346-372
R1-5in-r	TCAAGCGACGAGACTTGAATGTC	156-178
R1-3out-f	TGGTGAGTGACGAAGAGTTCCG	1543-1564
R1-3out-r	CACAAGCCGGTAAGCAGTCTTTT	277-299
R1-3in-f	CATCCGGCAGTTGCTAATCTCTC	1659-1681
R1-3in-r	CCTTCCTATGCTCCAGTCCTTGC	208-230
R2-5out-f	GCAAGGACTGGAGCATAGGAAGGAC	208-232
R2-5out-r	GGTCTTTTATCCCTGCGTCCG	246-266
R2-5in-f	TTACCTACTTAAAGGGAAATAGACGTG	346-372
R2-5in-r	CTTAACCTCCCAGTGAAGGAGGT	189-211
R2-3out-f	GACAAGTTGATGTTCGACCAGAC	1565-1587
R2-3out-r	CACAAGCCGGTAAGCAGTCTTTT	277-299
R2-3in-f	GGTTGCAGCTGACTCCAAGAATG	1681-1703
R2-3in-r	CCTTCCTATGCTCCAGTCCTTGC	208-230
lblv1com-R1-f	TGTAGACTGAGAACCAACCAACTC	1-24
lblv1com-R1-r	CCCCCCCCCTTCAGACCACTC	1762-1782
lblv1com-R2-f	TGTAGACTGAGAAAATTAAACCAAC	1-25
lblv1com-R2 -r	TTTTTTTTTTTTTTTTTTTTTTGATGTGCCGGGAGGC	1835-1871
**RT-PCR detection of different mycoviruses and dsRNAs**		
Primer Name	Sequence (5'→ 3')	Position[1]
LbLV1(RNA1)-f	TCAACTTTCCTATCCCGAAGG	1227-1247
LbLV1(RNA1)-r	ATCCCTACAGCACCACTAGGAGT	1704-1726
LbLV1(RNA2)-f	GACCAAGATCGCCAGCAGGC	571-590
LbLV1(RNA2)-r	ACGCTGTCGTCGCTCATGCACC	941-962
LbHV1-f	CTAGTTTTGATCTTGGAGACCG	270-291
LbHV1-r	TACGGGTGTCTATCTGCAGGTACC	720-743
LbBV2-f	GATCAGACGATGAGCACCTTGT	133-154
LbBV2-r	GGCATAGTTCCATTCACCCTG	564-584
LbTV1-f	TGCACCAAAAAACCTGGGCTCG	135-156
LbTV1-r	GAGCTGGGGGTAAGCATGTGTG	584-605
LbMV1-f	GAAACAAAACAATTATTTAATAT	273-295
LbMV1-r	GTTTGAATATACTGGTTTCGATAAT	717-741
LbMV3-f	CGAGTCAGTAGGATTCATTAAT	270-291
LbMV3-r	AGGGAGGGACCTTAAAATCTTGAATA	1055-1080
**Primers used for qPCR and overexpression vector construction**		
Primer Name	Sequence (5'→ 3')	Position[1]
jg3266-f_qpcr	CTCCCTACGACTATGGTGGTAAC	119-141
jg3266-r_qpcr	CCATACGCCAATGCGACAAC	340-359

jg2001-f_qpcr	TGACAACCTCCGCAAGGGTC	6-25
jg2001-r_qpcr	GAGGCTATCCTGGACGGAGC	227-246
jg4153-f_qpcr	CACCAAAGGTCGTCATCATCAT	467-488
jg4153-r_qpcr	ACGACAGCTGCGGTAGATGAC	679-699
jg5531-f_qpcr	CTTCGGAGAGATGATCGTTCAGT	582-604
jg5531-r_qpcr	GCAGATATCTCTCGTAAGCGGGC	789-811
251_g-f_qpcr	CGACCTGGCCCAGCTCCGC	60-78
251_g-r_qpcr	TGACTCCATAATTAACCTGGC	266-286
jg5437-f_qpcr	TCATAAGTCCCTTCCAAGCGA	59-79
jg5437-r_qpcr	GCAACCAGTTTGGCGAGGTA	256-275
4453_g-f_qpcr	TGGCATAATGTCCGTCATGTCC	174-195
4453_g-r_qpcr	AATGTGGTCGATGGGAGTGC	413-432
jg8174-f_qpcr	GGCAATTCACCGACAACATCC	290-310
jg8174-r_qpcr	TCAGCTCTGCAGAAGCCAAG	502-521
jg195-f_qpcr	TCTTCAAACTTCCGGCATGG	296-315
jg195-r_qpcr	CCTGGTTTCAATAGTCTCGGAC	503-524
lb_actin-f_qpcr	GAAGATGACCCAGATCGTCTTCG	351-373
lb_actin-r_qpcr	TGAGATCACGACCAGCCATGTC	535-556
lblv1-f_qpcr	GCTAGGTGGATGGCAAAATTTG	500-521
lblv1-r_qpcr	AGGTCCGGTGACGGCATTG	685-704

Vector and fusion PCR primers	
AV1_cp-f	TCGAGGTCGACGGTATCGATAAGCTT-ATGTCAGACACCTCATCTGTCAC
AV1_cp-r	TCCTCGCCCTTGCTCACCATAAGCTTA-AGGGTATTCAGCACGTTCG
jg2001_PKRGT-f	TCGAGGTCGACGGTATCGATAAGCTT-ATGTCTGACAACCTCCGCAAG
jg2001_PKRGT-r	TCCTCGCCCTTGCTCACCATAAGCTT-CTGCTTGCTGTTACCAGACAG
PKRGT-F(vector)	AGTCAGTCTCCCTTGCGGTT
PKRGT-R(vector)	AACTTCAGGGTCAGCTTGCC

to probe LbLV1

Primer Name	Sequence (5’→ 3’)	Position[1]
F-1 (RNA1)	TCAACTTTCCTATCCCGAAGG	1227-1247
R-1 (RNA1)	ATCCCTACAGCACCACTAGGAGT	1704-1726
F-1 (RNA2)	GACCAAGATCGCCAGCAGGC	571-590
R-1 (RNA2)	GGTGCATGAGCGACGACAGCGT	941-962

SSR primers

Primer Name	Primer sequence (5’→ 3’)	Repeat Motif
ssr14-f	GCCGTAGAAATAGGCCTTCC	CTA
ssr14-r	CAACGGAACCCTTCCAACTA	
ssr17-f	GACGACTGCACGACAACATC	GGA
ssr17-r	ACTCGCCTACCAACATGGAC	
ssr53-f	GTATGGGTGTTGATTTGGGG	TGG

The adaptor used for cDNA cloning of LbLV1

name	adaptor sequence (5’→ 3’)	Position[3]	reference
BcFV1-adaptor	GTGTGACAAATCAACCCTGAAATAG GGTTGACGACTCGACACGACAACAG	1-549	Hao, F., Ding, T., Wu, M.D., Zhang, J., Yang, L., Chen, W., and Li, G.Q. (2018).

	TTTCTTCTAAGTTGTCATGATTTAGT<br>AAGTCTAAAACTGTTCCACACCGAA<br>TTTCAAAGAAATTTTGAAAAACGAT<br>ACGGAAAGTTCTTGAATTGGACAAG<br>GCTTTTCCTGTCATAAGGCAAGATTG<br>TGCTTGTGTGCGCTCTGACCGAACCG<br>GTGGGCAAGGACTGGAGCATAGGAA<br>GGACCATTTAGTATGGGCTTAGTCGG<br>GCACAAGTGAACCGTGGGGTCCAAA<br>AGACTGCTTACCGGCTTGTGCTTAGG<br>GCTTAGTCCTGAAATTTGCGTAGTAC<br>CCCAGCTTCTGTTCTTACCTACTTAA<br>AGGGAAATAGACGTGGACACTAGAC<br>TACATGGAAAGACATGTTGACACAG<br>AATGAATAGTATTCTACAACCAACA<br>AAACTCAACATTTATGAAGACACCA<br>GACAAACCTGAACAAGTAATTATCC<br>GACACCCATTTAGTAATTTTAGTGAA<br>AGATGTGGTCCATAATTTTCTCAGTC<br>TACTGGCCTGAAGCC		Two novel hypovirulence-associated mycoviruses in the phytopathogenic fungus Botrytis cinerea: Molecular characterization and suppression of infection cushion formation. Viruses 10, 254.

[1] Positions for the PCR primers or the adaptor on the genome of corresponding mycoviruses
[2] “f” means “Forward” and “r” means “Reverse”.
[3] The adaptor is generated from the genome (nucleotide positions 1-549) of Botrytis cinerea fusarivirus 1

Table S4 The information of *Leptosphaeria biglobosa* strains used in biological experiments

Name	origin	LbLV1 detection
NN8-42	Nantong, Jiangsu Province, China	LbLV1+
N42-16	single conidium strain from the strain NN8-42	LbLV1-
EB9-21	Wuhan, Hubei Province, China	LbLV1+
E21-13	single conidium strain from the strain EB9-21	LbLV1-
W10	Wuxue, Hubei Province, China	LbLV1-
W10-9	W10 infected by LbLV1 particle	LbLV1+

Table S5 Relative abundance of viruses in each *Leptosphaeria biglobosa* group at species and family levels

virus species/families	g1	g2	g3	g4	g5	g6	g7	g8	g9	g10	g11
LbNV1	5.864158686	0	0.443353283	8.497977324	0.443828859	9.426764657	12.05758854	2.582242901	14.10340464	240.2705043	156.1949848
LbNV2	0	5.322660964	0.007347237	1.847990491	25.11470075	10.04829732	9.342426229	31.12946888	75.53466064	57.95895845	0
LbNV3	0	0	0	0	0	0	0	0	0	144.860831	0.124378522
LbNV4	0	0	0	0	0	0	0	0	0	4.34641615	0
LbNV5	0	0	0	0	0	0	0	0	0	7.770119933	0.034932814
LbNV6	0	0	0	0	0	3.724512254	0	4.83908061	0.191203923	4.734432697	0
LbNV8	0	0	0	0	0	0	0	0	0.505057813	0	0
LbNV9	0	0	0	0	0	0	0	0	0	151.4539934	0.645400823
LbMV1	3.01542895	2.216521978	0.013123745	0.011582134	0.210205159	0.9567175	0.900206685	0.072653209	0.044290895	0.006903652	0
LbMV2	0	0	0.076804414	0	0.246037762	0	0	0	0	0	0
LbMV3	20.90886014	10.57533943	2.53203386	0.848432047	6.378735253	4.215668612	2.3103376	1.761541129	1.759321918	0	0.007149017
LbMV4	0	0	0	0	0	0.127377154	0	0	0	0	0
LbMV5	0	0	0	0	0.129122375	0	0	0	0	0	0
LbMV6	0	0	0	0	0.106554846	0	0	0	0	0	0
LbMV7	0.348987323	0	0	0	0	0	0	0.173073693	0	0	0
LbMV8	0	0	0	0	0.110382424	0	0	0	0	0	0
LbMV9	0	0	0	0	0	0	0	0	0	0.007751892	3.026597299
LbBV1(ourmia)	0	0	0	0.576366649	0	0	0.011665981	4.105067643	2.973193991	0	0
LbBV2(ourmia)	0	0	0	0.08689501	0	0	0	0	0	0	0
LbLV1	0.686198512	0.790521077	1365.101768	4067.407463	0.755825153	4797.909532	2160.756723	3297.015595	1.190292065	0	0.019473491
LbDFLV1	0	3.866499267	0.112647696	54.7636156	0.007581078	19.39654221	0.679555571	0.003712018	0	0	0
LbDFLV2	0	0	0	0	2.012282839	0.176986121	0	0	0	0	0
LbTV1	0	0	0	0	0	0.624296204	0	0	0	0	0
LbALV1	0	0	0	0	0	0.113239793	0	0	0.100654	0	0
LbALV2	0	0	0	0	0	0.084742361	0	0	0	0	0
LbBFLV1	0	0	0	0	0	0	0.496847225	0	0	0	0
LbBFLV2	0	0	0	0.022566708	0.024092067	0.077669782	0.087698436	0.047185999	0.151019184	0	0
LbBFLV3	0	0	0.17068119	0.013693794	0.058477613	0.047131111	0.093129117	0.071582812	0.183281109	0	0
LbFLV1	0	0	0.009184297	5.888603854	0.004326658	23.58010762	84.25658588	22.5918515	65.60644564	12.10973662	0.004849135
LbNSV1	0	0	0	0	0.118792907	0	0	0	0	0	0
LbNSV2	0	0	0	0	0	0	0	0	0.567815384	0	0
LbNSV3	0	0	0.007098847	0.072047096	0	0	0.069997114	0.08514849	0.077862415	222.9524946	63.91374834
LbNSV4	0	0	0	0	0	0	0	0.528767111	0	0	0
LbNSV5	0	0	0	0	0	0.050477698	0	0	0	0	0
LbNSV6	0	0	0	0.123294233	0	0	0	0	0	0	0

LbNSV7	0	0	0.097208536	0	0	0	0	0	0	79.7941601	241.0254815
LbHV1	0	0	0	0	0.111545705	0	0	0	0	0	0
LbHV2	0	0	0	0	0.065175173	0	0	0	0	0	0
LbPV1	0	0	0	0	0	0.571347627	0	0.48256131	0	0	0
LbPV2	0	0	0	30.36777191	0	18.31374113	0	0.016331709	0	0	0
LbBV2	0	0	0	0	0	0	0	0	0	1.574822831	0
LbBV3	0	0	0	0	0	0	0	0	0	0.852767	0
Narnaviridae	5.864158686	5.322660964	0.45070052	10.34596782	25.55852961	23.19957423	21.40001477	38.55079239	90.33432702	611.3952559	156.999697
Mitoviridae	24.27327641	12.79186141	2.621962019	0.860014181	7.181037819	5.299763266	3.210544285	2.007268031	1.803612813	0.014655544	3.033746316
Botourmiaviridae	0	0	0	0.663261659	0	0	0.011665981	4.105067643	2.973193991	0	0
Deltaflexiviridae	0	3.866499267	0.112647696	54.7636156	2.019863917	19.57352833	0.679555571	0.003712018	0	0	0
Tymoviridae	0	0	0	0	0	0.624296204	0	0	0	0	0
Alphaflexiviridae	0	0	0	0	0	0.197982154	0	0	0.100654	0	0
Betaflexiviridae	0	0	0.17068119	0.036260502	0.08256968	0.124800893	0.677674778	0.118768811	0.334300293	0	0
Flaviviridae	0	0	0.009184297	5.888603854	0.004326658	23.58010762	84.25658588	22.5918515	65.60644564	12.10973662	0.004849135
Aspiviridae	0	0	0.097208536	0	0	0	0	0	0.567815384	79.7941601	241.0254815
Discoviridae	0	0	0.007098847	0.195341329	0	0	0.069997114	0.08514849	0.077862415	222.9524946	63.91374834
Mymonaviridae	0	0	0	0	0	0	0	0.528767111	0	0	0
Hypoviridae	0	0	0	0	0.176720878	0	0	0	0	0	0
Partitiviridae	0	0	0	30.36777191	0	18.88508876	0	0.498893019	0	0	0
Botybirnaviridae	0	0	0	0	0	0	0	0	0	2.427589831	0

Table S6 Viral sequence information obtained from high-throughput sequencing in *Leptosphaeria biglobosa*

ID	GenBank accession number	Contig length	full name	short name	Best match	coverage (%)	aa identity (%)	Genome type	Family/Genus
1	OP392999	2433	Leptosphaeria biglobosa narnavirus 1	LbNV1 RNA1	BCH36656.1 RNA-dependent RNA polymerase [Magnaporthe oryzae narnavirus 1]	95	78	+ssRNA	*Narnaviridae*
	OP393000	2432		LbNV1 RNA2	BCH36655.1 hypothetical protein [Magnaporthe oryzae narnavirus 1]	97	80		
2	OP441708	2360	Leptosphaeria biglobosa narnavirus 2	LbNV2 RNA1	XBY85591.1 RNA-dependent RNA polymerase [Exserohilum turcicum narnavirus 1]	94	81	+ssRNA	*Narnaviridae*
	OP441709	2227		LbNV2 RNA2	XBY85590.1 hypothetical protein [Exserohilum turcicum narnavirus 1]	94	76		
3	OP441710	2356	Leptosphaeria biglobosa narnavirus 3	LbNV3 RNA1	QZE12026.1 RNA-dependent RNA polymerase [Sclerotinia sclerotiorum narnavirus 2]	94	72	+ssRNA	*Narnaviridae*
	OP441711	2222		LbNV3 RNA2	QZE12027.1 hypothetical protein [Sclerotinia sclerotiorum narnavirus 2]	89	70		
4	OP441712	2028	Leptosphaeria biglobosa narnavirus 4	LbNV4 RNA1	WPE03611.1 RdRp [Beauveria bassiana splipalmivirus 1]	90	60	+ssRNA	*Narnaviridae*
	OP441713	2108		LbNV4 RNA2	WPE03612.1 RNA-dependent RNA polymerase [Beauveria bassiana splipalmivirus 1]	91	60		
5	OP441714	2028	Leptosphaeria biglobosa narnavirus 5	LbNV5 RNA1	WNA22220.1 RdRp [Downy mildew lesion associated splipalmivirus 44]	88	75	+ssRNA	*Narnaviridae*
	OP441717	2047		LbNV5 RNA2	WNA22221.1 RdRp [Downy mildew lesion associated splipalmivirus 44]	93	69		
6	OP441715	2051	Leptosphaeria biglobosa narnavirus 6	LbNV6 RNA1	WAK75249.1 RNA-dependent RNA polymerase [Narnaviridae sp.]	92	79	+ssRNA	*Narnaviridae*
	OP441716	2113		LbNV6 RNA2	XFO98523.1 RNA-dependent RNA polymerase [Narnavirus sp.]	90	65		
7	OP441667	1126	Leptosphaeria biglobosa narnavirus 8	LbNV8 RNA1	UAW09568.1 RNA-dependent RNA polymerase [Aspergillus flavus narnavirus 2]	97	84	+ssRNA	*Narnaviridae*
8	OP441668	1866	Leptosphaeria biglobosa narnavirus 9	LbNV9 RNA1	BDB16250.1 RdRp [Aspergillus tennesseensis narnavirus 1]	88	76	+ssRNA	*Narnaviridae*
	OP441669	1663		LbNV9 RNA2	QIR30287.1 RdRp [Plasmopara viticola lesion associated narnavirus 8]	79	70	+ssRNA	*Narnaviridae*
9	OP441670	2568	Leptosphaeria biglobosa mitovirus 1	LbMV1	YP_009553599.1 RNA-dependent RNA polymerase [Leptosphaeria biglobosa mitovirus 1]	88	100	+ssRNA	*Mitoviridae*
10	OP441671	2194	Leptosphaeria biglobosa mitovirus 2	LbMV2	CEZ26304.1 RNA dependent RNA polymerase [Grapevine associated narnavirus-1]	95	51	+ssRNA	*Mitoviridae*
11	OP441672	2489	Leptosphaeria biglobosa mitovirus 3	LbMV3	QTH80198.1 RNA-dependent RNA polymerase [Pestalotiopsis mitovirus 1]	84	98	+ssRNA	*Mitoviridae*

12	OP441673	2464	Leptosphaeria biglobosa mitovirus 4	LbMV4	UPW42182.1 putative RNA dependent RNA polymerase [Zhejiang mito-like virus 11]	88	63	+ssRNA	*Mitoviridae*
13	OP441674	2951	Leptosphaeria biglobosa mitovirus 5	LbMV5	QDH90004.1 RNA-dependent RNA polymerase [Mitovirus sp.]	77	100	+ssRNA	*Mitoviridae*
14	OP441675	3050	Leptosphaeria biglobosa mitovirus 6	LbMV6	XCN34970.1 RdRp [Alternaria alternata mitovirus 2]	82	80	+ssRNA	*Mitoviridae*
15	OP441676	539	Leptosphaeria biglobosa mitovirus 7	LbMV7	QXN75381.1 RNA-dependent RNA polymerase [Grapevine-associated mitovirus 6]	95	54	+ssRNA	*Mitoviridae*
16	OP441677	1745	Leptosphaeria biglobosa mitovirus 8	LbMV8	AKN79252.1 RdRp [Alternaria brassicicola mitovirus]	87	92	+ssRNA	*Mitoviridae*
17	OP441678	2287	Leptosphaeria biglobosa mitovirus 9	LbMV9	WZH58680.1 RNA-dependent RNA polymerase [Chrocavurn virus]	91	65	+ssRNA	*Mitoviridae*
18	OP441679	2511	Leptosphaeria biglobosa botourmiavirus 1	LbBV1	QDB75003.1 RdRp [Epicoccum nigrum ourmia-like virus 1]	78	53	+ssRNA	*Botourmiaviridae*
19	OP441680	2396	Leptosphaeria biglobosa botourmiavirus virus 2	LbBV2	WZH58586.1 RNA-dependent RNA polymerase [Chrocasuss virus]	73	89	+ssRNA	*Botourmiaviridae*
20	OQ650196	1782	Leptosphaeria biglobosa letobirnavirus 1	LbLV1 RNA1	QUE49128.1 replicase [Sclerotinia sclerotiorum virga-like virus 1]	92	97	+ssRNA	unclassified
	OQ650197	1871		LbLV1 RNA2	QUE49168.1 replicase, partial [Sclerotinia sclerotiorum virga-like virus 1]	87	96	+ssRNA	unclassified
21	OP441686	8409	Leptosphaeria biglobosa deltaflexivirus 1	LbDFV1	QTZ98076.1 RNA-dependent RNA polymerase [Alternaria alternata deltaflexivirus 1]	73	96	+ssRNA	*Deltaflexiviridae*
22	OP441687	7989	Leptosphaeria biglobosa deltaflexivirus 2	LbDFV2	UEP19789.1 polyprotein [Sclerotinia sclerotiorum deltaflexivirus 2-U]	41	46	+ssRNA	*Deltaflexiviridae*
23	OP441685	6507	Leptosphaeria biglobosa tymo-like virus 1	LbTV1	QQG34653.1 replicase [Triticum polonicum mycotymovirus 1]	34	43	+ssRNA	*Tymoviridae*
24	OP441703	1130	Leptosphaeria biglobosa alphaflexivirus 1	LbAFV1	UYL95418.1 RNA-dependent RNA polymerase [Nanning tick virus 2]	62	32	+ssRNA	*Alphaflexiviridae*
25	OP441704	1208	Leptosphaeria biglobosa alphaflexivirus 2	LbAFV2	UDL14008.1 RNA dependent RNA polymerase [Xiangshan tymo-like virus]	32	36	+ssRNA	*Alphaflexiviridae*
26	OP441688	1454	Leptosphaeria biglobosa betaflexivirus 1	LbBFV1	ARO70011.1 replicase [Grapevine rupestris stem pitting-associated virus]	99	99	+ssRNA	*Betaflexiviridae*
27	OP441689	1318	Leptosphaeria biglobosa betaflexivirus 2	LbBFV2	QQG34584.1 MP [Silene betaflexivirus 1]	71	44	+ssRNA	*Betaflexiviridae*
28	OP441690	2172	Leptosphaeria biglobosa betaflexivirus 3	LbBFV3	QEJ80631.1 polyprotein [Camellia ringspot associated virus 3]	83	96	+ssRNA	*Betaflexiviridae*
29	OP441691	7339	Leptosphaeria biglobosa flavi-like virus 1	LbFLV1	AZF86094.1 polyprotein [Sclerotium rolfsii alphavirus-like virus 2]	62	38	+ssRNA	*Flaviviridae*

30	OP441694	10692	Leptosphaeria biglobosa negative single-strand virus 1	LbNSV1	UTR30277.1 RNA-dependent RNA polymerase [Hymenoscyphus fraxineus negative stranded RNA virus 1]	65	28	-ssRNA	unclassified
31	OP441695	7111	Leptosphaeria biglobosa negative single-strand virus 2	LbNSV2	QJX19790.1 RdRp [Plasmopara viticola associated mycoophiovirus 4]	98	62	-ssRNA	*Aspiviridae*
32	OP441696	6390	Leptosphaeria biglobosa negative single-strand virus 3	LbNSV3 RNA1	XKM51328.1 RdRp [Grapevine wood holobiome associated mycobunyavirales-like virus 1]	99	65	-ssRNA	*Discoviridae*
	OP441701	1969		LbNSV3 RNA2	XKM51329.1 nonstructural protein [Grapevine wood holobiome associated mycobunyavirales-like virus 1]	81	33		
	OP441702	1256		LbNSV3 RNA3	XKM51330.1 nucleocapsid [Grapevine wood holobiome associated mycobunyavirales-like virus 1]	67	66		
33	OP441697	1529	Leptosphaeria biglobosa negative single-strand virus 4	LbNSV4	WPR17640.1 RdRp [Yellow silver pine associated mymonavirus 3]	72	29	-ssRNA	*Mymonaviridae*
34	OP441698	2535	Leptosphaeria biglobosa negative single-strand virus 5	LbNSV5	XKM51328.1 RdRp [Grapevine wood holobiome associated mycobunyavirales-like virus 1]	98	61	-ssRNA	unclassified
35	OP441699	4101	Leptosphaeria biglobosa negative single-strand virus 6	LbNSV6	QXN75415.1 RdRp [Grapevine-associated negative single-stranded RNA virus 5]	98	63	-ssRNA	*Discoviridae*
36	OP441700	7974	Leptosphaeria biglobosa negative single-strand virus 7	LbNSV7	QDB75017.1 RdRp [Cladosporium cladosporioides negative-stranded RNA virus 1]	87	78	-ssRNA	*Aspiviridae*
37	OP441692	3416	Leptosphaeria biglobosa hypovirus 1	LbHV1	AZT88613.1 polyprotein [Setosphaeria turcica hypovirus 1]	99	76	+ssRNA	*Hypoviridae*
38	OP441693	2436	Leptosphaeria biglobosa hypovirus 2	LbHV2	QDY81493.1 polyprotein [Bipolaris oryzae hypovirus 1]	99	58	+ssRNA	*Hypoviridae*
39	OP441681	1914	Leptosphaeria biglobosa partitivirus 1	LbPV1(RdRp)	QJW70310.1 RNA-dependent RNA polymerase [Erysiphe necator associated partitivirus 5]	84	93	dsRNA	*Partitiviridae*
	OP441682	1831		LbPV1(CP)	QED43035.1 CP [Podosphaera partitivirus D]	68	65	dsRNA	*Partitiviridae*
40	OP441683	1935	Leptosphaeria biglobosa partitivirus 2	LbPV2(RdRp)	ALM62247.1 RdRp [Soybean leaf-associated partitivirus 2]	91	90	dsRNA	*Partitiviridae*
	OP441684	1922		LbPV2(CP)	ALM62248.1 CP [Soybean leaf-associated partitivirus 2]	76	70	dsRNA	*Partitiviridae*
41	OP441705	5607	Leptosphaeria biglobosa botybirnavirus 2	LbBV2 RNA1	AMT92139.1 cap-pol fusion protein [Sclerotinia sclerotiorum botybirnavirus 2]	80	60	dsRNA	*Botybirnavirus*
	OP441706	4142		LbBV2 RNA2	AMT92140.1 hypothetical protein [Sclerotinia sclerotiorum botybirnavirus 2]	71	43		

42	OP441707	1185	Leptosphaeria biglobosa botybirnavirus 3	LbBV3	QUE49192.1 hypothetical protein [Sclerotinia sclerotiorum botybirnavirus 2-WX]	88	37	dsRNA	*Botybirnavirus*

Table S7 Viral alpha diversity indexes of *Leptosphaeria biglobosa* at species and family levels

**Species level**										
group	Simpson	Richness	Evenness	Shannon	Di	altitude	observed alleles	effective alleles	*H*	*I*
g1	0.49346796	5	0.5851838	0.941816993	54.6	571.6	3.6667	2.5266	0.5911	1.0146
g2	0.6901777	5	0.832863124	1.340441488	68.6	561	4.6667	2.4579	0.57	1.0171
g3	0.005060241	11	0.008431912	0.020218843	2.45	872	3	2.292	0.5591	0.8936
g4	0.04860894	14	0.055432619	0.146289859	19.7	30.34	5.6667	2.8598	0.6342	1.2202
g5	0.47508031	17	0.367119605	1.040128162	10.45	389.95	4	2.4767	0.5734	0.9979
g6	0.037029291	18	0.044567673	0.128817144	8.25	5	4.6667	3.6498	0.6757	1.2915
g7	0.093358532	12	0.094800007	0.235569169	NA	450	4.3333	2.6652	0.6003	1.1239
g8	0.040152089	16	0.046242877	0.12821248	28.21	23.3	5	2.9879	0.6621	1.2734
g9	0.615187649	14	0.440468462	1.162421522	73.14	19.3	6	3.4426	0.7003	1.4007
g10	0.812934773	14	0.692654503	1.827954944	38	2261	5	3.1355	0.6543	1.2913
g11	0.599556776	10	0.445307766	1.025359024	44	1813	4.6667	2.5807	0.5965	1.1376
**Family level**										
group	Simpson	Richness	Shannon	Evenness	Di	altitude	observed alleles	effective alleles	*H*	*I*
g1	0.313437919	2	0.492797056	0.710955869	54.6	571.6	3.6667	2.5266	0.5911	1.0146
g2	0.57175681	3	0.964153632	0.877610456	68.6	561	4.6667	2.4579	0.57	1.0171
g3	0.407739784	7	0.864791627	0.444414984	2.45	872	3	2.292	0.5591	0.8936
g4	0.617808498	8	1.17733129	0.566176671	19.7	30.34	5.6667	2.8598	0.6342	1.2202
g5	0.422048259	6	0.761397661	0.424944126	10.45	389.95	4	2.4767	0.5734	0.9979
g6	0.77746145	8	1.57432687	0.757091189	8.25	5	4.6667	3.6498	0.6757	1.2915

g7	0.377981096	7	0.695139562	0.357231069	NA	450	4.3333	2.6652	0.6003	1.1239
g8	0.569809925	9	1.054749507	0.480037188	28.21	23.3	5	2.9879	0.6621	1.2734
g9	0.523388935	8	0.855869779	0.411586362	73.14	19.3	6	3.4426	0.7003	1.4007
g10	0.501396627	6	0.900933377	0.502820492	38	2261	5	3.1355	0.6543	1.2913
g11	0.598359328	5	1.012935368	0.629372131	44	1813	4.6667	2.5807	0.5965	1.1376

Di: Disease incidence; H: genetic diversity index; I: Shannon Index

Table S8 Bray-curtis matrix of mycovirome at species and family levels

**Species level**											
group	g1	g2	g3	g4	g5	g6	g7	g8	g9	g10	g11
g1	0	0.497042053	0.994748145	0.996472387	0.768620583	0.995234918	0.991519215	0.996893288	0.913793118	0.98776247	0.976238257
g2	0.497042053	0	0.995032606	0.996487241	0.567916195	0.993830864	0.991278113	0.995306708	0.914763212	0.988797145	0.99989084
g3	0.994748145	0.995032606	0	0.506578607	0.994447904	0.562715563	0.24822222	0.422308223	0.995298384	0.999502804	0.9993683
g4	0.996472387	0.996487241	0.506578607	0	0.998119151	0.090008682	0.323568116	0.121846029	0.991245193	0.993601564	0.99628888
g5	0.768620583	0.567916195	0.994447904	0.998119151	0	0.993529814	0.988593562	0.983395585	0.716371793	0.946983239	0.99810428
g6	0.995234918	0.993830864	0.562715563	0.090008682	0.993529814	0	0.383529507	0.19117785	0.981606593	0.987859962	0.996467144
g7	0.991519215	0.991278113	0.24822222	0.323568116	0.988593562	0.383529507	0	0.220337149	0.925833233	0.979006746	0.991111994
g8	0.996893288	0.995306708	0.422308223	0.121846029	0.983395585	0.19117785	0.220337149	0	0.964484287	0.976411003	0.998590857
g9	0.913793118	0.914763212	0.995298384	0.991245193	0.716371793	0.981606593	0.925833233	0.964484287	0	0.845288208	0.954735456
g10	0.98776247	0.988797145	0.999502804	0.993601564	0.946983239	0.987859962	0.979006746	0.976411003	0.845288208	0	0.568454944
g11	0.976238257	0.99989084	0.9993683	0.99628888	0.99810428	0.996467144	0.991111994	0.998590857	0.954735456	0.568454944	0
**Family level**											
group	g1	g2	g3	g4	g5	g6	g7	g8	g9	g10	g11
g1	0	0.304871114	0.817141071	0.899080593	0.599597923	0.816416947	0.870770741	0.840381021	0.9201006	0.987737542	0.964057216
g2	0.304871114	0	0.749685889	0.839344123	0.490437695	0.744612436	0.860715616	0.837879156	0.922447453	0.988771521	0.965679184
g3	0.817141071	0.749685889	0	0.97230694	0.829982262	0.930086703	0.940720561	0.927828197	0.96928031	0.998758055	0.986415458
g4	0.899080593	0.839344123	0.97230694	0	0.807933112	0.428697269	0.832335408	0.785773054	0.865075988	0.968124959	0.959844375
g5	0.599597923	0.490437695	0.829982262	0.807933112	0	0.516140453	0.650764874	0.465645452	0.721076486	0.946919037	0.88561372
g6	0.816416947	0.744612436	0.930086703	0.428697269	0.516140453	0	0.514398764	0.394656123	0.614591729	0.930749476	0.905696567

g7	0.870770741	0.860715616	0.940720561	0.832335408	0.650764874	0.514398764	0	0.483174134	0.34417756	0.935333193	0.914794689
g8	0.840381021	0.837879156	0.927828197	0.785773054	0.465645452	0.394656123	0.483174134	0	0.425797749	0.898192661	0.847608202
g9	0.9201006	0.922447453	0.96928031	0.865075988	0.721076486	0.614591729	0.34417756	0.425797749	0	0.810902992	0.703918127
g10	0.987737542	0.988771521	0.998758055	0.968124959	0.946919037	0.930749476	0.935333193	0.898192661	0.810902992	0	0.568439007
g11	0.964057216	0.965679184	0.986415458	0.959844375	0.88561372	0.905696567	0.914794689	0.847608202	0.703918127	0.568439007	0
**Genetic distance matrix of *L. biglobosa* bassed on SSR methods**											
pop(host)	g1	g2	g3	g4	g5	g6	g7	g8	g9	g10	g11
g1	1	0.6805	0.7149	0.0512	0.0421	0.1477	0.325	0.4592	0.7631	0.9534	1.3577
g2	0.6805	1	0.7056	0.7161	0.6491	0.7605	0.2491	0.2524	0.2732	0.1445	0.2873
g3	0.7149	0.7056	1	0.036	0.0208	0.1793	0.2874	0.393	0.7096	0.9571	1.304
g4	0.0512	0.7161	0.036	1	0.0211	0.1228	0.2743	0.3281	0.5112	0.832	1.013
g5	0.0421	0.6491	0.0208	0.0211	1	0.1836	0.2778	0.3357	0.5953	0.8813	1.1173
g6	0.1477	0.7605	0.1793	0.1228	0.1836	1	0.4651	0.5351	0.5213	0.7006	0.7869
g7	0.325	0.2491	0.2874	0.2743	0.2778	0.4651	1	0.0458	0.1892	0.4236	0.5635
g8	0.4592	0.2524	0.393	0.3281	0.3357	0.5351	0.0458	1	0.086	0.3547	0.429
g9	0.7631	0.2732	0.7096	0.5112	0.5953	0.5213	0.1892	0.086	1	0.1844	0.1907
g10	0.9534	0.1445	0.9571	0.832	0.8813	0.7006	0.4236	0.3547	0.1844	1	0.1138
g11	1.3577	0.2873	1.304	1.013	1.1173	0.7869	0.5635	0.429	0.1907	0.1138	1

Table S9 Spatial distances between different *Leptosphaeria biglobosa* groups

	g1	g2	g3	g4	g5	g6	g7	g8	g9	g10	g11
g1	0	26887.99934	741526.3185	934871.1714	290618.6548	1250505.184	375134.28	696081.7104	768274.5512	608030.9265	552695.0721
g2	26887.99934	0	732038.0816	911582.4019	291011.901	1223834.763	348247.2064	670013.3542	742931.6539	630644.8611	574775.0532
g3	741526.3185	732038.0816	0	584487.7067	478346.4656	1282655.111	699329.1655	657880.0674	627560.7268	1287721.512	1242596.618
g4	934871.1714	911582.4019	584487.7067	0	821588.9688	749209.4513	638143.9608	324201.5341	226243.5275	1542065.997	1485649.853
g5	290618.6548	291011.901	478346.4656	821588.9688	0	1309816.377	468979.119	677533.4128	717198.5939	811106.2773	764775.3793
g6	1250505.184	1223834.763	1282655.111	749209.4513	1309816.377	0	879072.3374	650841.8264	656817.868	1756505.566	1697548.537
g7	375134.28	348247.2064	699329.1655	638143.9608	468979.119	879072.3374	0	345412.1516	433608.5328	942869.1704	883956.4098
g8	696081.7104	670013.3542	657880.0674	324201.5341	677533.4128	650841.8264	345412.1516	0	98566.0672	1285267.869	1226739.887
g9	768274.5512	742931.6539	627560.7268	226243.5275	717198.5939	656817.868	433608.5328	98566.0672	0	1365790.035	1307780.315
g10	608030.9265	630644.8611	1287721.512	1542065.997	811106.2773	1756505.566	942869.1704	1285267.869	1365790.035	0	59466.63105
g11	552695.0721	574775.0532	1242596.618	1485649.853	764775.3793	1697548.537	883956.4098	1226739.887	1307780.315	59466.63105	0

Table S10 KEGG pathway analysis of up-regulated and down-regulated expressed genes induced by LbLV1

**Up-regulated genes**								
Pathway ID	Pathway	level1	level2	List	Total	*P* value	Adjust *P* value	List_gene
zma00520	Amino sugar and nucleotide sugar metabolism	Metabolism	Carbohydrate metabolism	10	75	0.000730607	0.051873102	jg905(K01198);8401_g(K00844);jg1616(K01443);jg450(K01183);jg4459(K01183);jg4971(K00844);jg3394(K00698);jg4153(K00698);jg5255(K00820);jg5171(K00326)
zma00590	Arachidonic acid metabolism	Metabolism	Lipid metabolism	3	11	0.008550187	0.303531646	jg4000(K16342);jg6828(K08726);jg7225(K00079)
zma00100	Steroid biosynthesis	Metabolism	Lipid metabolism	4	30	0.031570972	0.413078558	jg8586(K05917);jg7939(K07750);jg8330(K00227);jg792(K00511)
zma00565	Ether lipid metabolism	Metabolism	Lipid metabolism	3	18	0.034387594	0.413078558	3449_g(K16794);jg4000(K16342);jg2154(K16794)
zma00062	Fatty acid elongation	Metabolism	Lipid metabolism	2	8	0.039263541	0.413078558	jg3959(K01074);jg3743(K10703)
zma00010	Glycolysis / Gluconeogenesis	Metabolism	Carbohydrate metabolism	5	48	0.04351025	0.413078558	8401_g(K00844);jg4635(K01689);jg2386(K00128);jg4971(K00844);jg2437(K00927)
zma00380	Tryptophan metabolism	Metabolism	Amino acid metabolism	6	64	0.0437883	0.413078558	jg9146(K00463);jg2386(K00128);jg9145(K01556);203_g(K03781);jg7221(K01426);jg7064(K01426)
zma00620	Pyruvate metabolism	Metabolism	Carbohydrate metabolism	5	50	0.050529558	0.413078558	565_g(K01512);jg2386(K00128);jg9387(K01512);jg6117(K00925);jg8939(K11262)
zma00500	Starch and sucrose metabolism	Metabolism	Carbohydrate metabolism	6	67	0.052889124	0.413078558	5632_g(K01199);jg5055(K01225);8401_g(K00844);jg4971(K00844);jg2888(K01176);jg5375(K19357)
zma00564	Glycerophospholipid metabolism	Metabolism	Lipid metabolism	5	52	0.058180079	0.413078558	jg2968(K00999);jg4000(K16342);jg7597(K17103);jg8330(K00227);4453_g(K17103)
zma00909	Sesquiterpenoid and triterpenoid biosynthesis	Metabolism	Metabolism of terpenoids and polyketides	1	2	0.079917796	0.515833049	jg792(K00511)
zma00903	Limonene and pinene degradation	Metabolism	Metabolism of terpenoids and polyketides	1	3	0.117472172	0.695043684	jg2386(K00128)
zma01040	Biosynthesis of unsaturated fatty acids	Metabolism	Lipid metabolism	2	16	0.136474558	0.739073346	jg435(K22993);jg3743(K10703)
zma00330	Arginine and proline metabolism	Metabolism	Amino acid metabolism	4	54	0.175847005	0.739073346	jg2386(K00128);jg1473(K01480);jg7221(K01426);jg7064(K01426)
zma00360	Phenylalanine metabolism	Metabolism	Amino acid metabolism	3	36	0.17928897	0.739073346	jg7221(K01426);jg3210(K00457);jg7064(K01426)

zma00591	Linoleic acid metabolism	Metabolism	Lipid metabolism	1	5	0.188088193	0.739073346	jg4000(K16342)
zma04146	Peroxisome	Cellular Processes	Transport and catabolism	5	77	0.202995994	0.739073346	jg4039(K00624);203_g(K03781);jg6828(K08726);jg4688(K11517);jg6372(K13299)
zma00040	Pentose and glucuronate interconversions	Metabolism	Carbohydrate metabolism	3	39	0.210952612	0.739073346	jg499(K01051);jg692(K01184);jg485(K22539)
zma00053	Ascorbate and aldarate metabolism	Metabolism	Carbohydrate metabolism	1	6	0.22126891	0.739073346	jg2386(K00128)
zma00052	Galactose metabolism	Metabolism	Carbohydrate metabolism	3	40	0.221775862	0.739073346	8401_g(K00844);jg4971(K00844);jg5854(K04618)
zma04070	Phosphatidylinositol signaling system	Environmental Information Processing	Signal transduction	2	23	0.240721686	0.739073346	jg2968(K00999);jg7010(K18083)
zma00790	Folate biosynthesis	Metabolism	Metabolism of cofactors and vitamins	2	24	0.256095475	0.739073346	jg4641(K15631);jg7225(K00079)
zma00561	Glycerolipid metabolism	Metabolism	Lipid metabolism	3	44	0.266054547	0.739073346	jg2386(K00128);jg2582(K18097);jg8330(K00227)
zma00770	Pantothenate and CoA biosynthesis	Metabolism	Metabolism of cofactors and vitamins	2	25	0.271490336	0.739073346	jg2386(K00128);jg8800(K01598)
zma00430	Taurine and hypotaurine metabolism	Metabolism	Metabolism of other amino acids	1	8	0.283655943	0.739073346	jg6117(K00925)
zma00592	alpha-Linolenic acid metabolism	Metabolism	Lipid metabolism	1	8	0.283655943	0.739073346	jg4000(K16342)
zma00562	Inositol phosphate metabolism	Metabolism	Carbohydrate metabolism	2	26	0.286879263	0.739073346	jg2968(K00999);jg7010(K18083)
zma00030	Pentose phosphate pathway	Metabolism	Carbohydrate metabolism	2	27	0.302237373	0.739073346	jg8703(K00033);jg119(K00852)
zma03018	RNA degradation	Genetic Information Processing	Folding, sorting and degradation	4	69	0.309498	0.739073346	jg4635(K01689);jg1712(K12586);jg3878(K12580);jg4561(K12604)
zma00750	Vitamin B6 metabolism	Metabolism	Metabolism of cofactors and vitamins	1	9	0.312967725	0.739073346	1476_g(K00275)
zma00480	Glutathione metabolism	Metabolism	Metabolism of other amino acids	3	49	0.322693996	0.739073346	2580_g(K00799);jg8703(K00033);jg6372(K13299)

zma00071	Fatty acid degradation	Metabolism	Lipid metabolism	2	31	0.362931524	0.746668403	jg2386(K00128);jg8051(K00249)
zma00310	Lysine degradation	Metabolism	Amino acid metabolism	2	31	0.362931524	0.746668403	jg2386(K00128);jg2591(K11419)
zma00910	Nitrogen metabolism	Metabolism	Energy metabolism	2	31	0.362931524	0.746668403	jg226(K01725);jg8357(K01673)
zma00740	Riboflavin metabolism	Metabolism	Metabolism of cofactors and vitamins	1	11	0.368075973	0.746668403	jg7541(K01078)
zma04016	MAPK signaling pathway - plant	Environmental Information Processing	Signal transduction	1	12	0.393965837	0.776988179	203_g(K03781)
zma00340	Histidine metabolism	Metabolism	Amino acid metabolism	1	13	0.41880538	0.787753601	jg2386(K00128)
zma00640	Propanoate metabolism	Metabolism	Carbohydrate metabolism	2	35	0.421614603	0.787753601	jg6117(K00925);jg8939(K11262)
zma00630	Glyoxylate and dicarboxylate metabolism	Metabolism	Carbohydrate metabolism	3	59	0.4353043	0.79247706	jg3114(K01569);203_g(K03781);jg4688(K11517)
zma00260	Glycine, serine and threonine metabolism	Metabolism	Amino acid metabolism	3	61	0.457147275	0.811436414	jg4524(K00130);jg7597(K17103);4453_g(K17103)
zma03010	Ribosome	Genetic Information Processing	Translation	6	137	0.490819016	0.821576657	jg3306(K02922);jg1468(K02995);jg3692(K02912);2746_g(K02957);jg933(K02936);jg7379(K02930)
zma03030	DNA replication	Genetic Information Processing	Replication and repair	2	41	0.504175177	0.821576657	jg3189(K10743);jg8132(K10756)
zma00130	Ubiquinone and other terpenoid-quinone biosynthesis	Metabolism	Metabolism of cofactors and vitamins	1	18	0.528667539	0.821576657	jg3210(K00457)
zma00730	Thiamine metabolism	Metabolism	Metabolism of cofactors and vitamins	1	18	0.528667539	0.821576657	jg7541(K01078)
zma00061	Fatty acid biosynthesis	Metabolism	Lipid metabolism	1	19	0.548034659	0.821576657	jg8939(K11262)
zma00410	beta-Alanine metabolism	Metabolism	Metabolism of other amino acids	1	19	0.548034659	0.821576657	jg2386(K00128)
zma03060	Protein export	Genetic Information Processing	Folding, sorting and degradation	1	20	0.566613771	0.821576657	jg4161(K03107)

zma00051	Fructose and mannose metabolism	Metabolism	Carbohydrate metabolism	2	46	0.567003609	0.821576657	8401_g(K00844);jg4971(K00844)
zma00280	Valine, leucine and isoleucine degradation	Metabolism	Amino acid metabolism	2	46	0.567003609	0.821576657	jg2386(K00128);jg8051(K00249)
zma00710	Carbon fixation in photosynthetic organisms	Metabolism	Energy metabolism	1	24	0.633666352	0.872027429	jg2437(K00927)
zma03040	Spliceosome	Genetic Information Processing	Transcription	4	107	0.644623126	0.872027429	jg5051(K12859);jg5685(K12837);jg2867(K12830);6516_g(K12878)
zma00920	Sulfur metabolism	Metabolism	Energy metabolism	1	25	0.648757019	0.872027429	jg6770(K00380)
zma00563	Glycosylphosphatidylinositol (GPI)-anchor biosynthesis	Metabolism	Glycan biosynthesis and metabolism	1	26	0.663232129	0.872027429	jg4259(K05291)
zma02010	ABC transporters	Environmental Information Processing	Membrane transport	1	26	0.663232129	0.872027429	7342_g(K05643)
zma03430	Mismatch repair	Genetic Information Processing	Replication and repair	1	29	0.703207792	0.882467077	jg8132(K10756)
zma04136	Autophagy - other	Cellular Processes	Transport and catabolism	1	29	0.703207792	0.882467077	jg4840(K08333)
zma00230	Purine metabolism	Metabolism	Nucleotide metabolism	2	61	0.720454059	0.882467077	jg8197(K01519);jg3645(K19572)
zma04141	Protein processing in endoplasmic reticulum	Genetic Information Processing	Folding, sorting and degradation	3	90	0.720888598	0.882467077	jg3677(K13525);jg9268(K06689);jg4686(K01230)
zma00513	Various types of N-glycan biosynthesis	Metabolism	Glycan biosynthesis and metabolism	1	35	0.769599503	0.905165836	jg4686(K01230)
zma00510	N-Glycan biosynthesis	Metabolism	Glycan biosynthesis and metabolism	1	36	0.779134658	0.905165836	jg4686(K01230)
zma00250	Alanine, aspartate and glutamate metabolism	Metabolism	Amino acid metabolism	1	37	0.788279062	0.905165836	jg5255(K00820)

zma04120	Ubiquitin mediated proteolysis	Genetic Information Processing	Folding, sorting and degradation	2	72	0.801883525	0.905165836	jg7715(K10259);jg9268(K06689)
zma03022	Basal transcription factors	Genetic Information Processing	Transcription	1	39	0.805458395	0.905165836	jg3576(K03128)
zma00350	Tyrosine metabolism	Metabolism	Amino acid metabolism	1	41	0.821256848	0.905165836	jg3210(K00457)
zma04145	Phagosome	Cellular Processes	Transport and catabolism	1	42	0.828672948	0.905165836	jg2481(K02144)
zma00970	Aminoacyl-tRNA biosynthesis	Genetic Information Processing	Translation	1	44	0.842603487	0.906437085	jg5535(K01873)
zma03420	Nucleotide excision repair	Genetic Information Processing	Replication and repair	1	47	0.861423683	0.912851963	jg8132(K10756)
zma04144	Endocytosis	Cellular Processes	Transport and catabolism	2	91	0.894463365	0.933924985	jg211(K12191);6700_g(K11839)
zma00190	Oxidative phosphorylation	Metabolism	Energy metabolism	2	99	0.919920253	0.946584608	jg2481(K02144);jg6405(K02272)
zma03008	Ribosome biogenesis in eukaryotes	Genetic Information Processing	Translation	1	74	0.956280744	0.969941897	jg5578(K14556)
zma03013	RNA transport	Genetic Information Processing	Translation	1	118	0.993523393	0.993523393	6516_g(K12878)
**Down-regulated genes**								
zma00520	Amino sugar and nucleotide sugar metabolism	Metabolism	Carbohydrate metabolism	12	75	9.73E-05	0.007294236	jg1392(K00698);737_g(K01443);jg5328(K17497);jg6534(K12373);251_g(K01183);jg278(K01809);3932_g(K01207);jg7474(K12373);jg2128(K00966);jg7574(K01183);3931_g(K01443);jg6999(K00326)
zma00511	Other glycan degradation	Metabolism	Glycan biosynthesis and metabolism	5	15	0.000365597	0.01370988	9627_g(K15923);5477_g(K01227);jg6534(K12373);jg8608(K01190);jg7474(K12373)
zma00531	Glycosaminoglycan degradation	Metabolism	Glycan biosynthesis	3	7	0.002781251	0.06953127	jg6534(K12373);3932_g(K01207);jg7474(K12373)

			and metabolism					
zma00604	Glycosphingolipid biosynthesis - ganglio series	Metabolism	Glycan biosynthesis and metabolism	2	5	0.018636705	0.349438221	jg6534(K12373);jg7474(K12373)
zma00460	Cyanoamino acid metabolism	Metabolism	Metabolism of other amino acids	4	26	0.02768905	0.415335756	jg6010(K01501);5473_g(K01501);jg5280(K05349);jg662(K05349)
zma00350	Tyrosine metabolism	Metabolism	Amino acid metabolism	5	41	0.035687677	0.446095966	jg6955(K00817);jg1654(K13953);jg4638(K00545);jg2495(K00451);jg6028(K13953)
zma00910	Nitrogen metabolism	Metabolism	Energy metabolism	4	31	0.049048543	0.512424996	jg6010(K01501);5473_g(K01501);jg2126(K01673);jg611(K01673)
zma00051	Fructose and mannose metabolism	Metabolism	Carbohydrate metabolism	5	46	0.054658666	0.512424996	jg5328(K17497);jg278(K01809);jg2128(K00966);jg5782(K18338);jg1525(K19355)
zma00270	Cysteine and methionine metabolism	Metabolism	Amino acid metabolism	6	63	0.063153342	0.52627785	jg709(K01738);jg9381(K00558);jg5260(K17989);5982_g(K01738);jg7092(K01611);8848_g(K00928)
zma00603	Glycosphingolipid biosynthesis - globo and isoglobo series	Metabolism	Glycan biosynthesis and metabolism	2	11	0.085845244	0.643839333	jg6534(K12373);jg7474(K12373)
zma03410	Base excision repair	Genetic Information Processing	Replication and repair	3	25	0.101293453	0.690637183	jg5684(K03660);3820_g(K03660);8196_g(K01247)
zma00965	Betalain biosynthesis	Metabolism	Biosynthesis of other secondary metabolites	2	14	0.130182534	0.813640838	jg2504(K15777);jg4638(K00545)
zma00300	Lysine biosynthesis	Metabolism	Amino acid metabolism	2	16	0.162075402	0.875928021	jg3275(K05824);8848_g(K00928)
zma00500	Starch and sucrose metabolism	Metabolism	Carbohydrate metabolism	5	67	0.185013249	0.875928021	jg5280(K05349);jg7125(K01193);jg662(K05349);4610_g(K01199);jg5217(K01210)
zma00730	Thiamine metabolism	Metabolism	Metabolism of cofactors and vitamins	2	18	0.195189729	0.875928021	jg1561(K01078);jg6072(K00788)
zma00261	Monobactam biosynthesis	Metabolism	Biosynthesis of other secondary metabolites	1	6	0.243325944	0.875928021	8848_g(K00928)

zma00940	Phenylpropanoid biosynthesis	Metabolism	Biosynthesis of other secondary metabolites	2	21	0.246161047	0.875928021	jg5280(K05349);jg662(K05349)
zma03030	DNA replication	Genetic Information Processing	Replication and repair	3	41	0.284042081	0.875928021	jg6614(K10739);158_g(K02540);jg4643(K02210)
zma00790	Folate biosynthesis	Metabolism	Metabolism of cofactors and vitamins	2	24	0.297590205	0.875928021	jg2953(K01113);jg562(K01724)
zma03440	Homologous recombination	Genetic Information Processing	Replication and repair	2	24	0.297590205	0.875928021	jg6614(K10739);jg4173(K10867)
zma00920	Sulfur metabolism	Metabolism	Energy metabolism	2	25	0.314659488	0.875928021	jg709(K01738);5982_g(K01738)
zma00563	Glycosylphosphatidylinositol (GPI)-anchor biosynthesis	Metabolism	Glycan biosynthesis and metabolism	2	26	0.331639943	0.875928021	jg8190(K03861);jg6847(K05292)
zma00750	Vitamin B6 metabolism	Metabolism	Metabolism of cofactors and vitamins	1	9	0.34196819	0.875928021	jg755(K00868)
zma00960	Tropane, piperidine and pyridine alkaloid biosynthesis	Metabolism	Biosynthesis of other secondary metabolites	1	9	0.34196819	0.875928021	jg6955(K00817)
zma03450	Non-homologous end-joining	Genetic Information Processing	Replication and repair	1	10	0.371928702	0.875928021	jg6138(K10981)
zma00010	Glycolysis / Gluconeogenesis	Metabolism	Carbohydrate metabolism	3	48	0.372621633	0.875928021	jg4120(K00627);jg1654(K13953);jg6028(K13953)
zma00600	Sphingolipid metabolism	Metabolism	Lipid metabolism	2	29	0.381792065	0.875928021	jg8875(K04709);jg8608(K01190)
zma03430	Mismatch repair	Genetic Information Processing	Replication and repair	2	29	0.381792065	0.875928021	jg6614(K10739);jg8598(K08736)
zma00100	Steroid biosynthesis	Metabolism	Lipid metabolism	2	30	0.398173136	0.875928021	jg60(K01852);jg5708(K07748)
zma00590	Arachidonic acid metabolism	Metabolism	Lipid metabolism	1	11	0.400537048	0.875928021	4591_g(K08726)

zma00740	Riboflavin metabolism	Metabolism	Metabolism of cofactors and vitamins	1	11	0.400537048	0.875928021	jg1561(K01078)
zma04122	Sulfur relay system	Genetic Information Processing	Folding, sorting and degradation	1	11	0.400537048	0.875928021	jg2248(K14168)
zma00071	Fatty acid degradation	Metabolism	Lipid metabolism	2	31	0.414354969	0.875928021	jg1654(K13953);jg6028(K13953)
zma00240	Pyrimidine metabolism	Metabolism	Nucleotide metabolism	2	31	0.414354969	0.875928021	jg7152(K00943);jg756(K01520)
zma00310	Lysine degradation	Metabolism	Amino acid metabolism	2	31	0.414354969	0.875928021	jg8019(K00474);jg4373(K06101)
zma00020	Citrate cycle (TCA cycle)	Metabolism	Carbohydrate metabolism	2	32	0.430321909	0.875928021	jg4120(K00627);jg2171(K00164)
zma00330	Arginine and proline metabolism	Metabolism	Amino acid metabolism	3	54	0.447061741	0.875928021	2809_g(K01469);jg7092(K01611);jg1495(K00318)
zma00340	Histidine metabolism	Metabolism	Amino acid metabolism	1	13	0.4539365	0.875928021	jg6955(K00817)
zma04146	Peroxisome	Cellular Processes	Transport and catabolism	4	77	0.465867159	0.875928021	jg9288(K13341);jg6433(K13337);jg1797(K01640);4591_g(K08726)
zma00513	Various types of N-glycan biosynthesis	Metabolism	Glycan biosynthesis and metabolism	2	35	0.476803068	0.875928021	jg6534(K12373);jg7474(K12373)
zma00072	Synthesis and degradation of ketone bodies	Metabolism	Lipid metabolism	1	14	0.478840652	0.875928021	jg1797(K01640)
zma04130	SNARE interactions in vesicular transport	Genetic Information Processing	Folding, sorting and degradation	1	15	0.502618956	0.876033733	jg731(K08497)
zma00650	Butanoate metabolism	Metabolism	Carbohydrate metabolism	2	38	0.520945496	0.876033733	5992_g(K01907);jg1797(K01640)
zma00260	Glycine, serine and threonine metabolism	Metabolism	Amino acid metabolism	3	61	0.529208849	0.876033733	jg5260(K17989);jg478(K00108);8848_g(K00928)
zma00040	Pentose and glucuronate interconversions	Metabolism	Carbohydrate metabolism	2	39	0.535105896	0.876033733	jg6417(K01728);jg44(K17818)
zma00290	Valine, leucine and isoleucine biosynthesis	Metabolism	Amino acid metabolism	1	17	0.546997568	0.876033733	jg5260(K17989)
zma00052	Galactose metabolism	Metabolism	Carbohydrate metabolism	2	40	0.54898114	0.876033733	jg7125(K01193);jg8608(K01190)

zma00061	Fatty acid biosynthesis	Metabolism	Lipid metabolism	1	19	0.587449613	0.886758508	jg4721(K00667)
zma00670	One carbon pool by folate	Metabolism	Metabolism of cofactors and vitamins	1	19	0.587449613	0.886758508	jg1344(K00297)
zma00860	Porphyrin and chlorophyll metabolism	Metabolism	Metabolism of cofactors and vitamins	1	21	0.624319575	0.886758508	jg5432(K08100)
zma00280	Valine, leucine and isoleucine degradation	Metabolism	Amino acid metabolism	2	46	0.626108492	0.886758508	5992_g(K01907);jg1797(K01640)
zma03013	RNA transport	Genetic Information Processing	Translation	5	118	0.629594288	0.886758508	jg5402(K14311);jg4843(K14307);6785_g(K14004);jg2201(K09291);jg159(K03259)
zma03420	Nucleotide excision repair	Genetic Information Processing	Replication and repair	2	47	0.637935586	0.886758508	jg6614(K10739);jg5290(K10570)
zma00400	Phenylalanine, tyrosine and tryptophan biosynthesis	Metabolism	Amino acid metabolism	1	23	0.657921974	0.886758508	jg6955(K00817)
zma00480	Glutathione metabolism	Metabolism	Metabolism of other amino acids	2	49	0.660716534	0.886758508	2809_g(K01469);jg2932(K00799)
zma03008	Ribosome biogenesis in eukaryotes	Genetic Information Processing	Translation	3	74	0.662113019	0.886758508	jg3516(K13288);jg3477(K14552);jg1207(K11129)
zma00770	Pantothenate and CoA biosynthesis	Metabolism	Metabolism of cofactors and vitamins	1	25	0.688543952	0.905978884	jg87(K00859)
zma02010	ABC transporters	Environmental Information Processing	Membrane transport	1	26	0.702820112	0.90881911	jg9293(K05658)
zma03040	Spliceosome	Genetic Information Processing	Transcription	4	107	0.726410139	0.912138039	jg8267(K12831);jg7015(K12832);jg5594(K12662);jg1404(K12627)
zma00900	Terpenoid backbone biosynthesis	Metabolism	Metabolism of terpenoids and polyketides	1	29	0.741872272	0.912138039	jg7587(K05906)
zma04136	Autophagy - other	Cellular Processes	Transport and catabolism	1	29	0.741872272	0.912138039	jg8009(K07203)

zma04141	Protein processing in endoplasmic reticulum	Genetic Information Processing	Folding, sorting and degradation	3	90	0.785984489	0.939601603	jg9347(K14002);6785_g(K14004);jg7195(K09584)
zma00380	Tryptophan metabolism	Metabolism	Amino acid metabolism	2	64	0.796893078	0.939601603	jg6010(K01501);5473_g(K01501)
zma03020	RNA polymerase	Genetic Information Processing	Transcription	1	35	0.805362129	0.939601603	8790_g(K03019)
zma00360	Phenylalanine metabolism	Metabolism	Amino acid metabolism	1	36	0.814321389	0.939601603	jg6955(K00817)
zma04120	Ubiquitin mediated proteolysis	Genetic Information Processing	Folding, sorting and degradation	2	72	0.847881603	0.962572201	jg7019(K03362);jg5290(K10570)
zma03050	Proteasome	Genetic Information Processing	Folding, sorting and degradation	1	43	0.866569446	0.962572201	jg7503(K03039)
zma00970	Aminoacyl-tRNA biosynthesis	Genetic Information Processing	Translation	1	44	0.872732129	0.962572201	3091_g(K01886)
zma00620	Pyruvate metabolism	Metabolism	Carbohydrate metabolism	1	50	0.904211747	0.982838856	jg4120(K00627)
zma00630	Glyoxylate and dicarboxylate metabolism	Metabolism	Carbohydrate metabolism	1	59	0.937540712	0.98757491	jg1965(K01637)
zma00230	Purine metabolism	Metabolism	Nucleotide metabolism	1	61	0.943215758	0.98757491	jg1924(K01522)
zma03015	mRNA surveillance pathway	Genetic Information Processing	Translation	1	65	0.953077538	0.98757491	1498_g(K14405)
zma03018	RNA degradation	Genetic Information Processing	Folding, sorting and degradation	1	69	0.961239579	0.98757491	jg1404(K12627)
zma04144	Endocytosis	Cellular Processes	Transport and catabolism	1	91	0.986531753	0.988688946	9586_g(K18467)
zma03010	Ribosome	Genetic Information Processing	Translation	2	137	0.988688946	0.988688946	jg7243(K02899);6926_g(K02910)
